# Nanotubular TiO_*x*_N_*y*_-Supported Ir Single Atoms and Clusters as Thin-Film Electrocatalysts for Oxygen Evolution in Acid Media

**DOI:** 10.1021/acs.chemmater.3c00125

**Published:** 2023-03-09

**Authors:** Luka Suhadolnik, Marjan Bele, Miha Čekada, Primož Jovanovič, Nik Maselj, Anja Lončar, Goran Dražić, Martin Šala, Nejc Hodnik, Janez Kovač, Tiziano Montini, Michele Melchionna, Paolo Fornasiero

**Affiliations:** † Department of Chemical and Pharmaceutical Sciences, CNR-ICCOM Trieste and INSTM Trieste Research Units, University of Trieste, via L. Giorgieri 1, 34127 Trieste, Italy; ‡ Department of Materials Chemistry, National Institute of Chemistry, Hajdrihova 19, SI-1000 Ljubljana, Slovenia; § Department of Thin Films and Surfaces, Jožef Stefan Institute, Jamova 39, SI-1000 Ljubljana, Slovenia; α Faculty of Chemistry and Chemical Technology, University of Ljubljana, Večna pot 113, SI-1000 Ljubljana, Slovenia; β University of Nova Gorica, Vipavska 13, SI-5000 Nova Gorica, Slovenia; ∥ Department of Analytical Chemistry, National Institute of Chemistry, Hajdrihova 19, SI-1000 Ljubljana, Slovenia; ⊥ Jožef Stefan International Postgraduate School, Jamova 39, SI-1000 Ljubljana, Slovenia; # Department of Surface Engineering, Jožef Stefan Institute, Jamova 39, SI-1000 Ljubljana, Slovenia

## Abstract

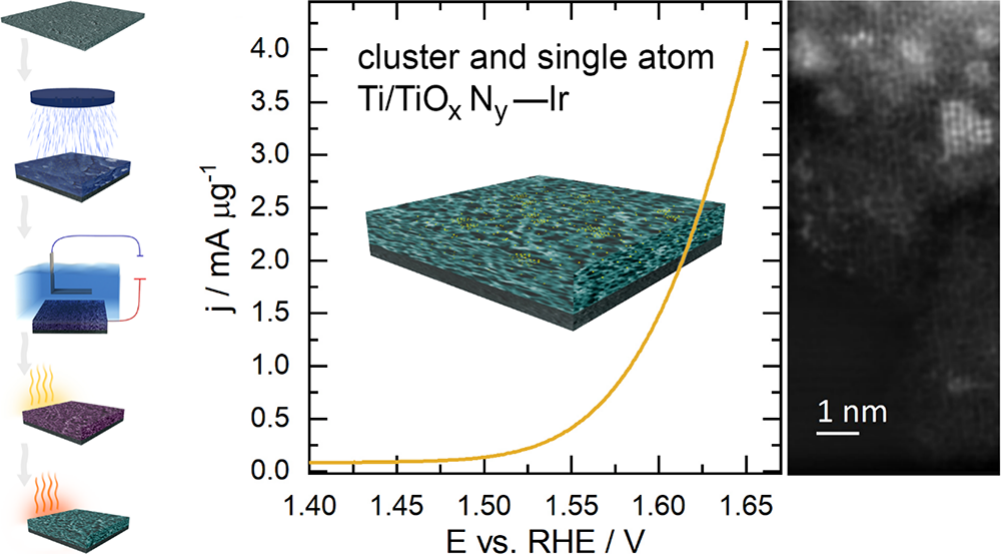

A versatile approach to the production of cluster- and single atom-based thin-film electrode composites is presented. The developed TiO_*x*_N_*y*_–Ir catalyst was prepared from sputtered Ti–Ir alloy constituted of 0.8 ± 0.2 at % Ir in α-Ti solid solution. The Ti–Ir solid solution on the Ti metal foil substrate was anodically oxidized to form amorphous TiO_2_–Ir and later subjected to heat treatment in air and in ammonia to prepare the final catalyst. Detailed morphological, structural, compositional, and electrochemical characterization revealed a nanoporous film with Ir single atoms and clusters that are present throughout the entire film thickness and concentrated at the Ti/TiO_*x*_N_*y*_–Ir interface as a result of the anodic oxidation mechanism. The developed TiO_*x*_N_*y*_–Ir catalyst exhibits very high oxygen evolution reaction activity in 0.1 M HClO_4_, reaching 1460 A g^–1^_Ir_ at 1.6 V vs reference hydrogen electrode. The new preparation concept of single atom- and cluster-based thin-film catalysts has wide potential applications in electrocatalysis and beyond. In the present paper, a detailed description of the new and unique method and a high-performance thin film catalyst are provided along with directions for the future development of high-performance cluster and single-atom catalysts prepared from solid solutions.

## 1 Introduction

The development of efficient, stable, and cost-effective electrocatalysts for the oxygen evolution reaction (OER) is crucial to enable our transition to a sustainable hydrogen economy.^1−5^

The state-of-the-art catalysts for the OER under acidic conditions are rare and expensive noble-metal oxides of iridium and ruthenium,^6,7^ among which IrO_2_ exhibits the best compromise between activity and stability.^8^ Two effective strategies to enhance its catalytic activity have been recently developed constituting of the preparation of a heterogeneous interface between Ir nanoclusters and IrO_2_^9^ or creating an ultrasmall Ir sphere shell around the IrO_2_ core.^10^ However, in previous years, there was considerable research devoted to the development of new catalysts with reduced noble-metal content. Popular approaches focused on the synthesis of catalysts with core–shell morphologies and minimal Ir content and/or dispersing the noble-metal nanoparticles on a high-surface-area support or mixing them with less expensive oxides of earth-abundant elements.^11−13^ Examples include reduction of Ir loading by combining core–shell Ir/metal (Fe, Co, or Ni) nitride morphologies,^11^ dispersing the active IrO_*x*_ phase on the TiN–Ti support,^14^ uniformly dispersing IrO_*x*_ nanoparticles on M-SnO_2_ (M = Nb, Ta, or Sb) supports,^15^ and immobilizing Ir nanoparticles on a conductive indium tin oxide support.^12^ Another example of the latter approach was also investigated in our previous studies, which have demonstrated the potential of TiO_*x*_N_*y*_ prepared by anodic oxidation and annealing in ammonia as support for OER catalysts in acid.^16,17^ TiO_*x*_N_*y*_ is one of the most promising support materials due to its high conductivity and stability (in combination with Ir) in the extremely harsh conditions of OER operation. The beneficial activity and stability synergy observed between TiO_*x*_N_*y*_ and Ir is called “strong metal–support interaction (SMSI)”, a well-known phenomenon in heterogeneous catalysts.^18^ However, the SMSI effect in electrocatalysis, in particular for OER, is much less known and explored. The main reason is that supports favoring the SMSI are ceramic materials that are usually semiconductive. Recently, a few studies have shown this effect on Pt-based systems,^19,20^ whereas our research has shown that SMSI is indeed possible for an Ir-based system,^21^ which was later confirmed by experimental data and DFT calculations in studies focused on the development of nanotubular TiO_*x*_N_*y*_–Ir OER catalysts.^16,17^

The SMSI effect is significantly enhanced in the case of single-atom catalysts (SACs), which provide exceptional atom utilization efficiency.^22,23^ It is well known that catalytic performance is controlled by the accessible surface area and intrinsic activity per active site, which has been shown to be maximized with the utilization of SACs.^24−26^ However, the synthesis of SACs for OER that do not contain carbon is still in its infancy, with not many developed preparation strategies for the Ir SACs,^27^ in particular in acidic media. Examples of synthetic strategies include an in situ cryogenic–photochemical reduction method (for Ir single-atom sites on NiFe oxyhydroxides),^22^ electrochemical methods (for Ir SAC on a nickel-iron sulfide nanosheet array substrate),^25^ co-electrodeposition methods (Ir SAC on ultrathin NiCo_2_O_4_ nanosheets),^28^ chemical reduction method (Ir SAC on CoO_*x*_ amorphous nanosheets with abundant surface absorbed oxygen;^29^ Ir SAC incorporated in Co-based hydroxide nanosheets^30^), and thermal decomposition method (Ir SAC on the NiO matrix).^31^ It is crucial to design closely defined new material systems in which the Ir content can be minimized and the OER performance in acid can be tuned with regard to the catalyst’s composition, morphology, and structure down to the atomic level.

In this regard, an alternative method to Ir SAC and single-atom and cluster catalyst (SACC) preparation involving the anodic oxidation of solid solutions is for the first time presented in the present article. The anodic oxidation process enables the synthesis of ordered high-surface-area oxide nanostructured films on a metal substrate. It is a relatively straightforward, versatile, and low-cost synthetic technique for the preparation of immobilized highly porous electrocatalyst thin films that constitute electrodes without further manipulation. Anodic oxidation of a wide range of materials has been showcased^32−35^ including anodic oxidation of a few alloys.^36^ Anodization of noble metal-containing Ti alloys results in the oxide nanotube layers that are self-decorated with noble metal nanoparticles.^37^ The reported TiO_2_ material includes Pt nanoparticles of 4–5 nm in diameter and not Pt SACC since the starting Ti–Pt alloy had a Pt content of 0.2 at %, which is more than the maximum amount of Pt that still assures the formation of only α-Ti solid solution and the formation of SACC.^38^ Despite the large number of articles in the field of anodic oxidation, no one has yet prepared a SACC. In addition, the rare anodic oxidation of alloys has never been used to synthesize a noble metal-containing electrocatalyst. What has been studied instead is the decoration of an anodic TiO_2_ nanotube layer with Ir nanoparticles^39^ and with Ir single atoms.^40^

In the present work, we focused on the development of TiO_*x*_N_*y*_-supported Ir SACC for the oxygen evolution reaction. A novel thin-film electrode material was prepared from α-Ti solid solution, which is completely different from preparing the TiO_*x*_N_*y*_–Ir catalyst from pure titanium and deposition of Ir in the form of nanoparticles in the last synthesis step as recently reported.^16,17^ We outlined the key conditions of TiO_*x*_N_*y*_–Ir catalyst preparation to maximize the OER performance with the use of a record-low amount of Ir in the Ir-based OER catalysts. In this regard, anodization conditions as well as two different heat treatments were thoroughly optimized. By modifying α-Ti solid solution, we found that the specific surface area of the Ti–Ir thin film is increased with the anodic oxidation process, whereas heat treatments increase the mechanical stability and electronic conductivity of the novel Ir SACC electrode. Through the study of the TiO_*x*_N_*y*_–Ir catalyst and its synthetic intermediates, we demonstrated why the Ir SACCs are formed and under what synthetic conditions they show the best OER performance in acid. We achieved exceptional Ir atom utilization efficiency resulting in a mass electrocatalytic activity of ∼450 A g^–1^_Ir_ at 1.55 V (vs RHE) and a Tafel slope of 89 mV dec^–1^. The new synthetic method of Ir SACC on a high-surface-area support can be extended to other SACCs for various other electrocatalytic applications and beyond. Its main advantages are the ability to prepare SACC on various conductive substrates, excellent control over the synthesis parameters and thus over the properties of the prepared thin films, the homogeneity of the prepared SACC, high specific surface area and strong attachment of SACC, and last but not least, the possibility of preparing a large number of different materials.

## 2 Materials and Methods

### 2.1 Cleaning of Ti Substrates and Triode Sputtering of Ti–Ir

Ti metal foils (200 μm thick, 99.8%, Baoji Lyne Metals Co., Ltd.) were cleaned in acetone in an ultrasonic bath, rinsed with ethanol, and dried under a stream of nitrogen to remove organic contaminants. A Ti–Ir sputtering target was prepared from titanium target (60 mm diameter × 9 mm thickness, 99.99%, PI-KEM) and Ir wire (1.0 mm diameter × 50 mm length, 99.9%, Goodfellow), which was cut into six pieces and incorporated into the Ti target at six positions shown in Figure S1. All Ir wires were inserted inside the area where the impinging argon ion flux is at its maximum, i.e., within a circle with a diameter of 20 mm. The deposition was performed in the triode sputtering system Balzers Sputron. After evacuation, the samples were heated to 120 °C until the base pressure of 5 × 10^–6^ mbar was reached. During the coating phase, the plasma is formed between the tantalum filament and the anode ring surrounding the target (50 V, 50 A). Sputtering is sustained by applying 1700 V to the target at the current of 0.6 A, yielding a deposition rate of around 10 nm/min. The sputtering time was adjusted to deposit approximately 20, 200, and 1000 nm-thick Ti–Ir films. Sputtered substrates were denoted as Ti–Ir_20, Ti–Ir_200, and Ti–Ir_1000, respectively.

### 2.2 Anodization of Sputtered Ti–Ir Films and Ti Metal Foils

Substrates with and without Ir were subjected to potentiostatic anodization in a two-electrode electrochemical cell (Figure S2) using a platinum counter electrode. Substrates were first cut into 15 × 15 mm squares and then cleaned with acetone and ethanol. The area of substrates exposed to the electrolyte was fixed at approximately 0.785 cm^2^; the part of a substrate that was exposed to the electrolyte had a diameter of approximately 10 mm. Anodization was performed in an electrolyte consisting of 0.3 wt % NH_4_F (99.99%, Sigma-Aldrich) and 2 vol % deionized water in ethylene glycol (99.5%, Carlo Erba Reagents). The anodization voltage was varied between 10 and 120 V, and the anodization time was varied between 5 s and 5 h. The selected anodization voltage was kept constant during anodization. The anodized samples were washed with deionized water and dried under nitrogen stream.

### 2.3 Post-Treatment of Anodized Films

Anodized samples were first annealed at 450 °C for 15 min to 60 h in air. The second annealing was performed in an ammonia atmosphere at 700 °C for 5 min to 2 h. The flow of ammonia gas was varied between 50 and 300 cm^3^ min^–1^.

### 2.4 Materials Characterization Techniques

#### 2.4.1 SEM and SEM–EDXS Analysis

The surface morphology of all samples was examined by means of SEM using a Zeiss FE-SEM SUPRA TM 35 VP (Carl Zeiss, Oberkochen, Germany) field-emission scanning electron microscope equipped with an energy-dispersive X-ray spectrometer (EDXS) SDD EDX Ultim Max 100 (Oxford Instruments, Oxford, UK). The operating voltage was set to 7 kV for both analyses.

#### 2.4.2 XRD

The samples were characterized with X-ray diffraction using a D5000 Bruker AXS diffractometer with Cu–Kα radiation (λ = 1.5406 Å). The diffractograms were measured in the 2θ angular range between 20° and 100° with a step size of 0.04° and a collection time of 1 s. The phase identification was performed with the X’Pert HighScore Plus program using the International Centre for Diffraction Data (ICDD) PDF-4+ 2021 database.^41^

#### 2.4.3 XPS

The surface composition of Ti–Ir and TiO_*x*_N_*y*_–Ir films was analyzed with X-ray photoelectron spectroscopy (XPS) on a PHI-TFA XPS spectrometer (Physical Electronics Inc.) equipped with an Al-monochromatic source of X-rays. The high-energy resolution XPS spectra were recorded on the surface of samples as well as at a depth of 4 or 40 nm, i.e., after the XPS depth profile. Carbon was not taken into account since it was assumed to be present due to contamination.

#### 2.4.4 ToF-SIMS

Composition of the TiO_*x*_N_*y*_–Ir sample was evaluated by time-of-flight secondary ion mass spectrometry (ToF-SIMS). The mass spectra of positive and negative secondary ions emitted from the surface were acquired by a ToF-SIMS 5 instrument (ION TOF) using a Bi^+^ ion beam of 30 keV for spectrum excitation and a Cs^+^ ion beam at 2 keV for ion sputtering during depth profile analyses. The SIMS spectra were collected during depth profile analyses from the surface to the depth of 400 nm.

#### 2.4.5 FIB

Ti–Ir and TiO_*x*_N_*y*_–Ir lamellas for (scanning) transmission electron microscopy ((S)TEM) were prepared using a FEI Helios Nanolab 650, FEI, Hillsboro, OR, USA, focused ion beam (FIB). During the preparation of the Ti–Ir lamella (Figure S3), it was protected with an approximately 300 nm-thick electron-deposited Pt layer and an additional approximately 2.5 μm-thick ion-deposited Pt layer, which were deposited on top of each other at the selected ion acceleration voltages/beam currents of 20 kV/1.6 nA and 30 kV/0.4 nA, respectively. During the preparation of the TiO_*x*_N_*y*_–Ir lamella, it was protected with an approximately 300 nm-thick electron-deposited C layer and an additional approximately 2.5 μm-thick ion-deposited C layer since deposition of Pt results in Pt nanoparticles inside the porous TiO_*x*_N_*y*_–Ir film, rendering the analysis of Ir in TiO_*x*_N_*y*_–Ir impossible. Both lamellas were extracted with gallium ions at 30 kV/21 nA and transferred with a OmniProbe 200 micromanipulator to the FIB lift-out grids. For the Ti–Ir lamella, an EM-tech Cu grid was used, whereas an EM-tech Mo grid was used for the TiO_*x*_N_*y*_–Ir lamella. In the last step, the final thinning and polishing of lamellas were performed at 1 kV/100 pA for 1 min on each side, enabling the removal of the amorphous residue and gallium artifacts and reaching the desired thickness of <20 nm, enabling atomic resolution in STEM.

#### 2.4.6 TEM Techniques

TEM imaging was performed in a Cs-corrected transmission electron microscope (CF-ARM Jeol 200) equipped with an SSD JEOL EDX spectrometer and a GATAN Quantum ER dual-electron energy loss spectroscopy (EELS) spectrometer. An operational voltage of 80 kV was employed. The images were taken in STEM mode (BF and HAADF) at 6C and 3 cm effective camera length. EDXS analysis was performed at probe size 2C and 8 cm effective camera length. Besides the TEM analysis of Ti–Ir and TiO_*x*_N_*y*_–Ir lamellas, the TEM analysis of a scratched TiO_*x*_N_*y*_–Ir catalyst from the Ti substrate was also performed to completely get rid of the possible Pt contamination.

#### 2.4.7 ICP-MS

The iridium content was determined according to the following protocol. The samples were cut so that only the treated (anodized and/or heat-treated) area of the sample was submerged in 8 mL of boiling aqua regia (3:1 HCl:HNO_3_ v/v, concentrated) and subsequently sonicated for 10 min. In the case of the Ti–Ir alloy samples, the exact sample area was determined before these samples were placed in aqua regia. During sonication in hot aqua regia, all the surface coating containing Ir in the Ti–Ir and TiO_*x*_N_*y*_–Ir samples was etched away and dissolved into aqua regia. This was subsequently diluted 100-fold with 2% HNO_3_, and the Ir concentration was measured with an ICP-MS instrument (Agilent 7900x). The net mass of Ir in the sample was then calculated from the concentrations/dilutions.

#### 2.4.8 LA-ICP-MS

The instrumental setup used in this work for laser ablation ICP-MS (LA-ICP-MS) measurements was comprised of a laser ablation system (193 nm ArF* excimer; Analyte G2 Teledyne Photon Machines Inc., Bozeman, MT). The LA system was equipped with a standard active two-volume ablation cell (HelEx II). The LA unit was coupled to a quadrupole ICP-MS instrument (Agilent 7900x, Agilent Technologies, Santa Clara, CA). Ablation parameters were as follows: laser fluence, 5.0 J cm^–2^; repetition rate, 20 Hz; beam diameter, 35 μm (square mask); dosage, 10; total acquisition time for ICP-MS acquisition, 0.5 s (with corresponding dwell time for specific nuclide ^193^Ir, 0.4998 s). Other parameters were based on model predictions for fastest possible mapping times, avoidance of aliasing, minimal blur, and maximal S/N ratios.^42^ For the quantification, a flattened iridium wire was used as a standard (99.9%, Merck) and ablation volumes were measured with an optical profilometer Zygo (Zegage PRO HR, USA). Data processing and image analysis were performed using the software packages HDIP (Teledyne Photon Machines Inc., Bozeman, MT) and ImageJ.

#### 2.4.9 XANES

X-ray absorption spectroscopy (XAS) experiments at the Ir L_3_ edge were performed at the SAMBA beamline of synchrotron SOLEIL.^43^ Measurements were performed in fluorescence mode with a 35 pixel HPGe detector (Mirion/Canberra) in conjunction with DxMap DSP (XIA), while ionization chambers were filled with nitrogen at 1 bar (7.5% absorption at 12 keV). Measurements of reference materials were performed in transmission. To refine the XANES spectra, the data were analyzed using the DEMETER suite (Athena software to extract and normalize signal). Considering the poor signal-to-noise ratio derived from the very low Ir content in the samples, at least 20 spectra were averaged to obtain the data presented. Due to the very low Ir loading in the samples, the acquisition of the EXAFS spectra with acceptable signal-to-noise ratio was not possible, also after acquisition of a large number of spectra.

### 2.5 Electrochemical Measurements

The OER performance was evaluated in the same single-compartment cell (Figure S2) as was used for the anodization of the samples but in a three-electrode configuration. All the prepared materials were used as a working electrode, which was positioned at the bottom of the cell with the catalyst surface facing upward. A reversible hydrogen electrode (RHE) was used as a reference electrode, and a graphite rod was used as a counter electrode. The supporting electrolyte was Ar-saturated 0.1 M HClO_4_ aqueous solution. The potential was controlled with a potentiostat (SP-300, Biologic). The potential was first cycled between 0.05 and 1.65 V vs a reversible hydrogen electrode (RHE) for 100 cycles at 300 mV s^–1^ to obtain a stable cyclic voltammogram (CV). After the pretreatment, the OER activity was evaluated under the same conditions but with a scan rate of 20 mV s^–1^. The resistance of the electrolyte was recorded by electrochemical impedance spectroscopy (EIS) before each measurement, and the iR compensation of 85% was applied. To determine OER charge transfer resistance (*R*_ct_), electrochemical impedance spectroscopy measurements (EIS) were performed in the same electrochemical setup by measuring the EIS spectra (Nyquist plots) at 1.6 V vs RHE in the frequency range from 5000 to 5 Hz. The EIS response was further analyzed based on a simplified equivalent circuit taken from Watzele et al.^44^ The circuit parameters were obtained by the Z view program fitting the points to a semicircle. An ST-CV stability test was performed by cycling the voltage between 0.05 and 1.65 V at 300 mV/s for 335 cycles. After the degradation protocol, LSV was performed under the same conditions prior to the degradation protocol.

Determination of the specific surface area of the TiO_*x*_N_*y*_–Ir catalyst was done electrochemically with the measurement of the double layer capacitance of TiO_*x*_N_*y*_–Ir and AO-TiO_*x*_N_*y*_–Ir reference materials. The latter was prepared with anodization in the fluoride-free electrolyte at 60 V for 5 min. Both materials were first cycled between 0.05 and 1.65 V vs a reversible hydrogen electrode (RHE) for 100 cycles at 300 mV s^–1^, after which open circuit potential (OCP) was determined. The *C*_dl_ of the catalysts was determined by measuring the capacitive current associated with double-layer charging from the scan-rate dependence of cyclic voltammetry stripping. CV curves were measured around open circuit potential (OCP) at scan rates of 2, 4, 6, 8, 10, and 20 mV s^–1^. The *C*_dl_ was estimated by plotting the average of the anodic (*j*_a_) and cathodic (*j*_c_) current density against the scan rate, in which the slope is *C*_dl_. The average double layer capacitive current density (*j*_avg_) is given by the equation *j*_avg_ = (*j*_a_ + |*j*_c_|)/2 = *C*_dl_ × ν, where ν is the scan rate. The specific surface area was determined by multiplying the roughness factor *R*_f_ and the real surface area of a smooth electrode, *S* (ECSA = *R*_f_ × *S*).^45^ The latter is equal to the geometric area of the electrode, whereas *R*_f_ is estimated from the ratio of double-layer capacitance *C*_dl_ for the catalyst in focus and the corresponding smooth electrode.

IrO_2_ benchmark material (Alfa Aesar) was measured in a three-electrode configuration glassy cell with a thin film of IrO_2_ powder deposited on a glassy carbon RDE, which was used as a working electrode and HydroFlex reversible hydrogen electrode (Gaskatel GmbH), and carbon rod as reference and counter electrodes, respectively. Thin films were prepared by drop-casting 20 μL of IrO_2_ ink, prepared by dispersion of nanoparticles in water and 2-propanol in 7:1 ratio with pH adjusted to 11 by 1 M KOH solution. Additionally, 5% Nafion stock solution (Aldrich) was added to the ink so that the amount of Nafion was 25 wt % of the solid content in the suspension. The electrochemical protocol consisted of five fast cycles (100 mV s^–1^) between 0.05 and 1.45 V and subsequent activity measurement by executing three cycles between 1.2 and 1.6 V with a scan rate of 5 mV s^–1^.

### 2.6 Optical Profilometry Measurements of the Ti–Ir Sputtering Target

Topography of the worn target shown in Figure S4 was evaluated by the Bruker Dektak XT profiler. The scanning parameters were as follows: stylus diameter, 2.5 μm; scanning area, 2 × 2 mm^2^; line spacing, 20 μm; horizontal resolution, 1.3 μm; vertical resolution, 20 nm.

## 3 Results and Discussion

### 3.1 Influence of Synthesis Conditions on TiO_*x*_N_*y*_–Ir Preparation

**Figure 1:**
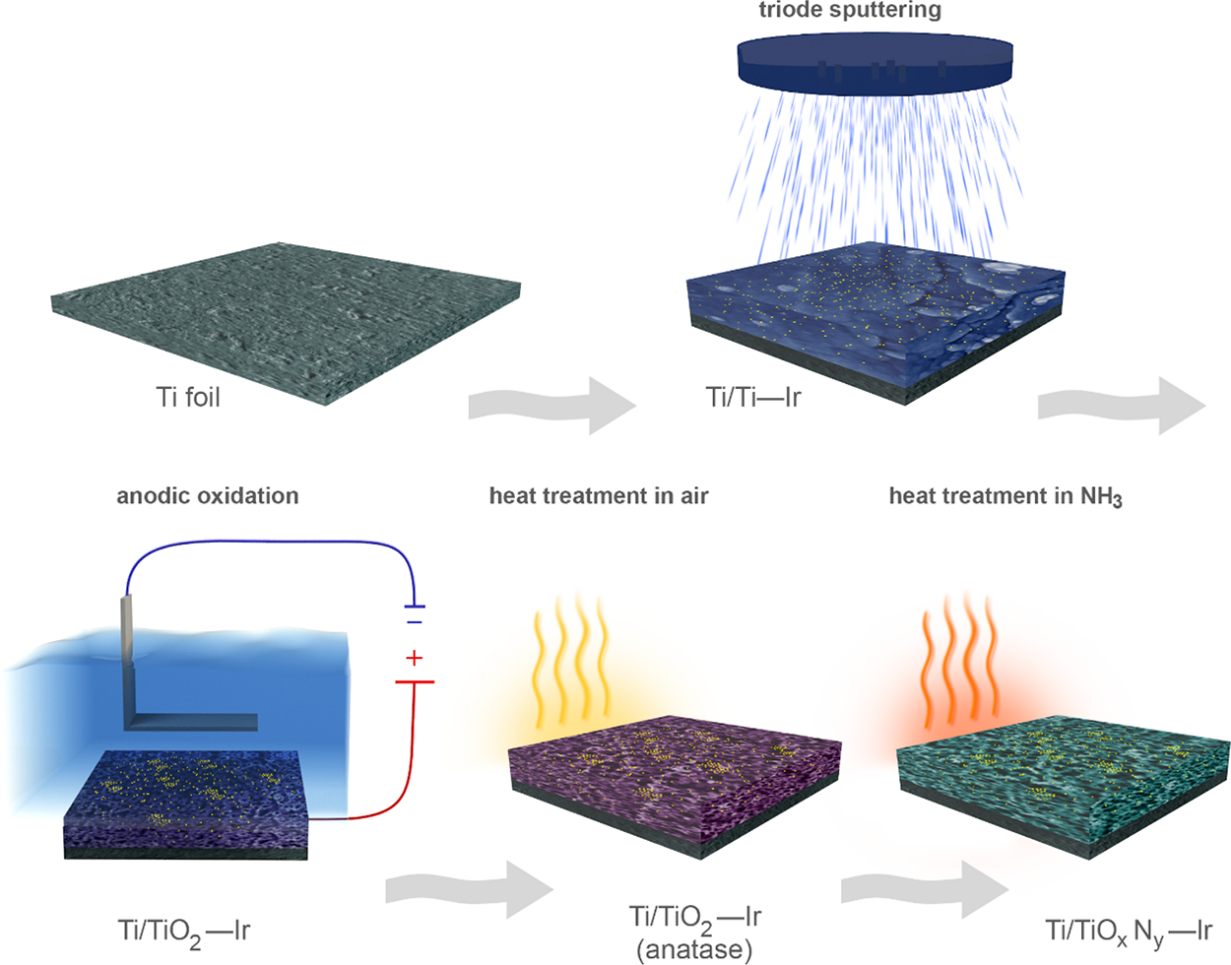
Sketch of the synthetic procedure of TiO_*x*_N*_y_–*Ir catalyst preparation.

The TiO_*x*_N_*y*_–Ir catalyst was prepared in an immobilized form following the procedure shown in Figure 1. The synthetic conditions for TiO_*x*_N_*y*_–Ir (anodization electrolyte aging, anodization voltage and time, annealing temperatures, and amount of deposited Ir) were thoroughly optimized. The catalyst was prepared by applying a new synthetic concept consisting of the use of the triode sputtering method for the thin-film preparation that was in the next step exposed to the anodic oxidation. This strategy was never used before for the synthesis of Ir SACC. More specifically, for TiO_*x*_N_*y*_–Ir preparation, the sputtering is carried out simultaneously with Ti and Ir (the sputtering target is prepared from Ti and Ir, detailed description of its preparation is found in Section 2, and the exact positions where Ir wire is inserted into the Ti target are shown in Figure S1) onto the Ti foil substrate.

To optimize the TiO_*x*_N_*y*_–Ir film morphology, chemical composition, and most importantly, the electrocatalytic activity, the synthetic conditions were thoroughly investigated. The sputtered Ti–Ir film was anodized at voltages ranging from 10 to 120 V and anodization times ranging from 5 s to 5 h. Freshly prepared and aged electrolytes with 0.3 wt % NH_4_F, 2 vol % H_2_O, and ethylene glycol were alternatively used and compared. The duration of air annealing performed at 450 °C was varied from 15 min to 60 h, whereas the duration of ammonia annealing at 700 °C was varied from 5 min to 2 h, while different ammonia flow rates (50, 100, or 300 cm^3^ min^–1^) were explored.

The preliminary results obtained in the course of optimization of TiO_*x*_N_*y*_–Ir catalyst synthesis showed important trends that were taken into account when selecting the optimal synthesis parameters for the preparation of the catalyst for further advanced detailed analysis. In detail, several relevant parameters influence the morphology, chemical composition, and electrocatalytic activity of TiO_*x*_N_*y*_–Ir, namely, (i) the anodization voltage and time, (ii) the anodization electrolyte age, (iii) the annealing time at 450 °C in air, and (iv) the ammonia flow rate and the annealing time at 700 °C in an ammonia atmosphere. The most efficient catalyst is prepared when anodization of the approximately 200 nm-thick Ti–Ir film is performed for 3 to 8 min at 60 V (Figures S5 and S6). The anodization process is followed by annealing of the catalyst in air at 450 °C for 1 h and annealing in ammonia at 700 °C for 15 min at the NH_3_ flow rate of 300 cm^3^ min^–1^. Such conditions ensure a complete anodization of the Ti–Ir film, high Ir content, creation of highly porous TiO_*x*_N_*y*_–Ir morphology, and an appropriate N/O ratio in TiO_*x*_N_*y*_–Ir for optimal conductivity and catalytic properties. Optimization of TiO_*x*_N_*y*_–Ir catalyst synthesis is briefly described in Section 2.1, whereas thorough description of the influence of many synthetic conditions can be found in the Supporting Information.

#### 3.1.1 Characterization of Ti–Ir and Influence of Its Film Thickness

Ti–Ir alloy with the most suitable thickness was chosen based on the mass electrocatalytic activity of the final catalysts. Since our goal was to prepare a single atom-based TiO_*x*_N_*y*_–Ir catalyst, the Ir amount inserted into the Ti sputtering target was selected according to the appearance of α-Ti solid solution in the Ti–Ir phase diagram, which is the only stable phase up to 1 at % Ir.^46^ If the Ir content is increased above 1 at %, an intermetallic phase Ti_3_Ir is also formed up to the Ir content of 25 at % when a single-phase region of Ti_3_Ir starts. Increasing the Ir amount in the starting Ti–Ir alloy would therefore result in the formation of Ti_3_Ir, thus reducing the amount of Ir single atoms in the alloy and significantly changing the starting alloy for anodization, preventing the formation SACC as has been shown in the case of Ti–Pt anodization where Pt nanoparticles were formed.^37^ As determined with XPS, SEM–EDXS, and STEM–EDXS (Figure S7) measurements (Table S1), there is approximately 0.8 ± 0.2 at % Ir in the Ti–Ir alloy if the presence of other nonmetal elements (oxygen, carbon, and nitrogen) is disregarded. XRD diffractograms (Figures S8 and S9) of Ti–Ir alloy show distinct peaks related to the hexagonal metal titanium foil substrate as well as three additional peaks (labeled with a solid diamond in Figure S8, bottom) that are related to sputtered α-Ti solid solution with very small grain size (Figure S10).^47,48^ Samples with approximately 20 nm-, approximately 200 nm-, and approximately 1000 nm-thick Ti–Ir films were therefore prepared by means of triode sputtering, anodized, and annealed in air and ammonia to yield the final TiO_*x*_N_*y*_–Ir_20, TiO_*x*_N_*y*_–Ir_200, and TiO_*x*_N_*y*_–Ir_1000 catalysts. The number at the end of the sample name denotes the approximate starting Ti–Ir film thickness. The correlation between thickness and mass electrocatalytic activity was then explored to allow choosing of the most suitable film thickness, with the sample TiO_*x*_N_*y*_–Ir_200 exhibiting the highest activity among the three samples (Figure S11). We rationalized the significantly lower mass electrocatalytic activity of the thickest catalyst on the basis of its high Ir content (approximately 4.1 μg cm^–1^ as determined with ICP-MS, which is more than six times higher than in TiO_*x*_N_*y*_–Ir_200), which is in a great extent inaccessible under the selected anodization conditions, as it remains in the Ti–Ir film below the anodized sample. On the other hand, the observed much lower activity of the thinnest film (TiO_*x*_N_*y*_–Ir_20) (Figure S11) originates from the too low amount of Ir (approximately 0.079 μg cm^–1^ as determined with ICP-MS) after the anodization at 60 V for 1 min (Table S2). Such a poor electrochemical performance of TiO_*x*_N_*y*_–Ir_20 is also in line with its surface morphology (Figure S12) where it is possible to observe anodized and non-anodized regions. Anodized regions show typical TiO_2_–Ir morphology, which has not yet been etched away due to very short anodization time. Extending the anodization time to just a few more minutes results in the nanostructured film covering the entire catalyst surface, while the upper film containing Ir would already be fully etched away. Regardless of the film thickness, the average TiO_2_–Ir etching rate is greater than 300 nm h^–1^ and lower than 1000 nm h^–1^ as determined by the analysis of Ti–Ir films of all thicknesses that were anodized for extended times (1, 3, or 5 h; Figures S13 and S14). The morphology of Ti–Ir alloy of all three different thicknesses is additionally shown in Figure S15. Based on these findings, we concluded that 20 and 1000 nm-thick Ti–Ir films are not the appropriate starting materials for TiO_*x*_N_*y*_–Ir electrocatalyst synthesis due to too low or too high Ir content, respectively, and we focus the study on TiO_*x*_N_*y*_–Ir_200.

#### 3.1.2 Influence of Ti–Ir Anodization Time and Voltage

The influence of the anodization time on the anodized film morphology was determined at 60 V, and it is shown by means of SEM (Figure S16). The influence of anodization voltage on the film morphology was also investigated (Figure S17). Both figures show amorphous TiO_2_–Ir nanotubes, which were in the next steps converted to Ir-incorporated anatase and later into titanium oxynitride. Both anodization time and voltage have a similar effect on the anodized film morphology and Ir content in the amorphous TiO_2_. SEM–EDXS analysis indicates that increasing the anodization time or voltage decreases the Ir content in the anodized film (this trend is shown in Table S3) and increases the film thickness. At the same time, the Ti content is decreased and F and O content is increased. As far as Ir is concerned, the quantification is not accurate due to the very low Ir content with respect to the intrinsic limits of quantification of the technique. Nevertheless, EDXS is helpful in defining the general trends.

The most notable effect of increasing anodization time or voltage is the more pronounced etching of TiO_2_–Ir, resulting in an Ir-richer bottom part of the anodized film than the top as confirmed with in-depth EDXS analysis throughout the entire thickness of the anodized film with Ir. More specifically, it turns out that the Ir content at the bottom of the approximately 380 nm-thick film is about 2.0 wt % Ir, significantly higher than that at the top (∼1.1 wt % Ir), while the Ir content in the substrate below the delaminated anodized film is not detectable with this technique, implying very low levels of Ir (Figure S18 and Table S4). The difference in the thickness of the Ti–Ir film and TiO_2_–Ir film formed with anodization is explained with the mechanism of the anodic oxidation process.^49^ During anodization, the Ti–Ir film is oxidized and the TiO_2_–Ir film is simultaneously etched by fluorine ions, resulting in the formation of a thicker nanoporous TiO_2_–Ir film.

The changes in the surface roughness of the anodized films can be readily noticed. Samples anodized at voltages ranging from 10 to 30 V for 5 min possess less defined nanostructured morphology with smaller pores (Figure S17). Samples anodized for 2 min or more at 60 V are fully covered with a high-density porous structure, whereas films anodized for shorter times at this anodization voltage show regions with very low pore density (Figure S16). Increasing the anodization time slightly increases the surface roughness, which is the most significant feature for the sample anodized at 60 V for 8 min (Figure S16h). This is the longest anodization time under these conditions (Ti–Ir film thickness, anodization voltage, and electrolyte age and composition), which gives electrocatalytically active films. Upon further extending the anodization time, all TiO_2_–Ir is etched away and the film morphology gradually changes to high-aspect-ratio nanotubes with highly defined and ordered tube openings (Figure S14). The morphology of anodized Ti–Ir significantly differs from anodized Ti (Figure S13).

#### 3.1.3 Influence of Post-Treatments of Anodized Ti–Ir

Finally, we explored the modification of the materials with post-anodization annealing treatments. Annealing performed at 450 °C in air is needed to convert the amorphous anodized film to anatase TiO_2_ and improve its mechanical stability to tolerate the second heat treatment done at higher temperatures (700 °C) in NH_3_. Such a second heat treatment converts the anatase TiO_2_ to an electrically conductive TiO_*x*_N_*y*_ structure.^16,50^ The duration of both annealing processes was optimized with morphological characterization and electrocatalytic activity measurements of the prepared films. Results showed that the optimal annealing time in air is 1 h since reducing the annealing time (15 and 30 min) resulted in a decreased electrocatalytic activity, whereas extending annealing times (24 and 60 h) resulted in film delamination during the heat treatment.

Nitridation in NH_3_ at 700 °C considerably affects the TiO_2_–Ir film because it influences (i) the anodized Ti–Ir film morphology (Figures S19 and S20), (ii) the Ir content (Table S5), and (iii) the N/O ratio determining the support electronic conductivity. The nitridation time and NH_3_ flow rate were accurately optimized, and in general, given that anodized Ti–Ir films are very thin, a shorter nitridation time was required as compared to conventional protocols found in the literature and also reported in our recent studies on TiO_*x*_N_*y*_ catalyst supports.^16,17^ The too long nitridation time results in a loss of the nanostructured morphology (Figure S21) and a significant reduction of the amount of Ir in the sample. Best nitridation conditions allowed complete retaining of the nanostructured morphology, with N replacing a part of O atoms in the TiO_2_ structure depending on the anodization procedure. Thicker films anodized at higher voltages and longer times can be nitridated for longer time at higher flow rate in comparison to samples anodized at low anodization voltages (below 30 V for 5 min) or short anodization times (less than 2 min at 60 V).

### 3.2 Full Characterization of TiO_*x*_N_*y*_–Ir

The most efficient TiO_*x*_N_*y*_–Ir catalyst selected in Section 2.1 was characterized by means of various techniques. The TiO_*x*_N_*y*_–Ir catalyst was prepared from approximately 200 nm-thick Ti–Ir alloy with anodization at 60 V for 5 min. The anodization electrolyte was freshly prepared and allowed to age for three different time durations (5, 25, or 170 min) before anodizing the final samples so that a comparison could be made also on the basis of differently aged electrolytes and to understand the electrolyte effect. These were annealed in air at 450 °C for 1 h followed by annealing in NH_3_ at 700 °C for 15 min at the NH_3_ flow rate of 300 cm^3^ min^–1^. SEM, EDXS, and XRD analyses were performed in each stage of catalyst preparation. Additionally, ICP-MS, XPS, ToF-SIMS, XANES, and STEM analyses of Ti–Ir_200 and TiO_*x*_N_*y*_–Ir were carried out. Electrochemical analysis of each stage of catalyst preparation was also performed, and it is described in Section 2.3 together with the analysis of the composition and morphology of samples after electrochemical treatments.

**Figure 2:**
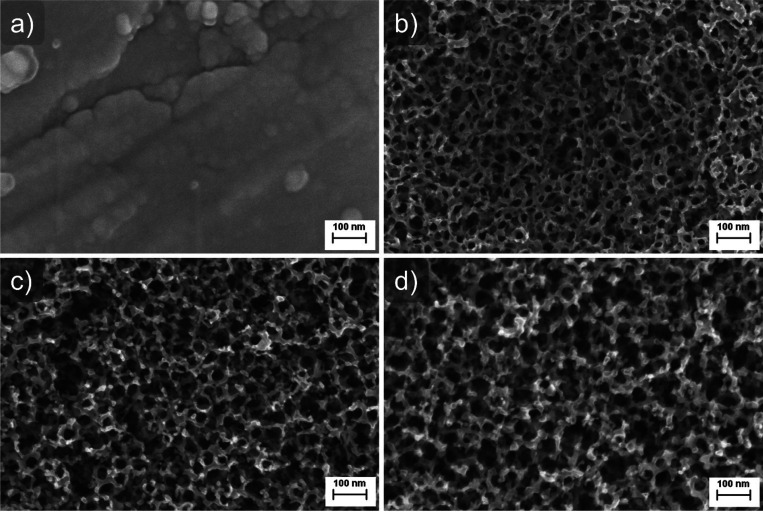
Top surface morphology of the TiO_*x*_N_*y*_–Ir catalyst in each stage of its preparation: (a) sputtered approximately 200 nm-thick Ti–Ir film, (b) anodized Ti–Ir film at 60 V for 5 min, (c) air-annealed anodized Ti–Ir film, and (d) ammonia-annealed final TiO_*x*_N_*y*_–Ir catalyst.

Figure 2 shows the top surface morphology of the TiO_*x*_N_*y*_–Ir catalyst in each stage of its preparation recorded with SEM analysis. It can be seen that the approximately 200 nm-thick Ti–Ir film is homogeneous and is covering the entire Ti substrate (Figure 2a). During anodization, a porous nanostructured film is grown, which shows nanopore openings similar to the top morphology of vertically aligned TiO_2_ nanotubes (Figure 2b).^51,52^ Annealing in air increases the surface roughness of the anodized film (Figure 2c), whereas nitridation introduces small pores into the film top surface and further increases the surface roughness (Figure 2d). The TiO_*x*_N_*y*_–Ir film thickness (determined by means of SEM analysis of a detached part of one sample (Figure S22)) was calculated to be about 380 nm. Additionally, the film is not made of well-defined nanotubes but of poorly defined pore structures (Figure S22b).

**Figure 3:**
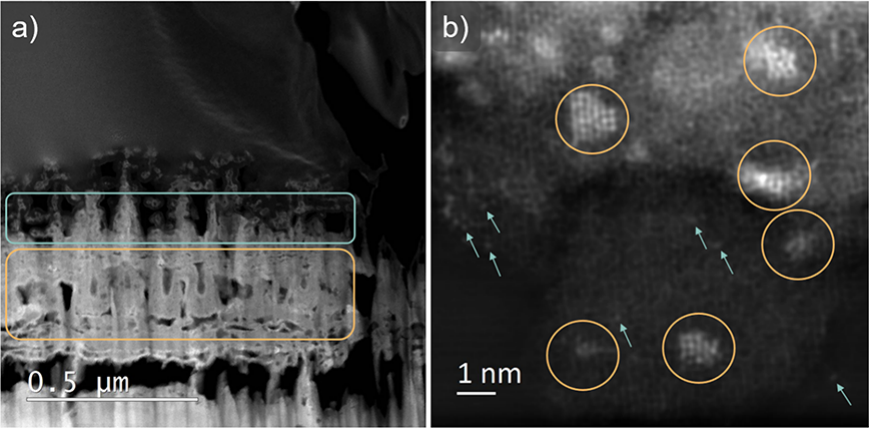
STEM analysis of (a) the cross-sectioned FIB lamella of the TiO_*x*_N_*y*_–Ir catalyst and (b) TiO_*x*_N_*y*_–Ir catalyst scratched on a TEM grid. The region with smaller pores is marked with an orange rectangle, whereas a blue rectangle marks the region with larger pores in panel (a). The Ir nanoclusters are marked with orange circles, whereas blue arrows point to Ir single atoms in panel (b). The TiO_*x*_N_*y*_–Ir catalyst was prepared from the approximately 200 nm-thick Ti–Ir film anodized at 60 V for 5 min, air-annealed at 450 °C for 1 h, and ammonia-annealed at 700 °C for 15 min at the ammonia flow rate of 300 cm^3^ min^–1^.

STEM analysis of the TiO_*x*_N_*y*_–Ir lamella reveals that the catalyst has porous morphology with many differently oriented nanotubes (Figure 3a). The pores are present throughout the entire film thickness; however, two types of porous regions of the TiO_*x*_N_*y*_–Ir lamella are present, one with larger macropores and one more compact with smaller mesopores. Interconnected mesopores of TiO_*x*_N_*y*_ support are known to be responsible for the relatively large specific surface area and improved mass transport.^3,53−55^ The regions with larger pores have a higher nitrogen content than those with smaller pores, where higher amounts of oxygen are found (Figure S23). The EDXS mapping also shows more or less homogeneous distribution of Ir, which is not in line with the SEM–EDXS measurement of the catalyst cross section where more Ir is present at the Ti/TiO_*x*_N_*y*_–Ir interface. The reason for this discrepancy is the result of Pt contamination of the TiO_*x*_N_*y*_–Ir FIB lamella. In fact, although the FIB lamella was carefully prepared with C protection layers on a Mo grid, Pt was likely introduced in the pores of the catalyst since it was used to mount the lamella on the Mo grid. Since L and M Ir and Pt lines are partially overlapping, a detailed analysis of the catalyst containing both Pt and Ir is impossible. Consistently, a strong Ir EDXS signal was observed in the pure Pt layer (Figure S23). EDXS analysis of the part of the sample with higher concentration of nanoclusters confirmed that the clusters are ascribed to Ir (Figure S24). Nevertheless, due to the presence of Pt in the TiO_*x*_N_*y*_–Ir lamella, the same sample was also scratched on a TEM grid and analyzed to get the accurate information about the size and shape of Ir in the final catalyst. TEM analysis shown in Figure 3b revealed approximately 1 nm large Ir clusters and some Ir single atoms on a TiO_*x*_N_*y*_ support. The amount of Ir is much higher in the form of nanoclusters. The existence of Ir single atoms is additionally confirmed with HAADF-STEM images shown in Figure S25.

The chemical composition of the TiO_*x*_N_*y*_–Ir catalyst was determined with ICP–MS, SEM–EDXS, STEM–EDXS, and XPS, as shown in Table 1. Additionally, the chemical composition of the synthetic intermediates is shown in Table S1. The N/O ratio of the final TiO_*x*_N_*y*_–Ir catalyst depends on the nitridation time and ammonia flow rate. XPS elemental composition of TiO_*x*_N_*y*_–Ir was measured on the surface and in the subsurface region (depth of approximately 40 nm). It can be seen that the surface composition is enriched in O with respect to composition in the subsurface region where N is relatively increased. This is due to the surface oxidation of the TiO_*x*_N_*y*_–Ir sample during storage in the air, which was already reported before. It was shown that surface oxidation does not lower the support electrical conductivity.^21^ The N/O ratio on the surface is approximately 0.41 and increases to approximately 1.31 at the TiO_*x*_N_*y*_–Ir depth of 40 nm. At the same time, the (N + O)/Ti ratio is decreased from approximately 2.64 to 1.38 at the depth of 40 nm, implying that on the surface, titanium is oxidized.

**Table 1:** Chemical Composition of the TiO_*x*_N_*y*_–Ir Catalyst Determined with ICP-MS, XPS, SEM–EDXS, and STEM–EDXS

	Ti	O	N	Ir
ICP-MS (μg cm^–2^)				0.64
XPS (wt %) (surface)	53.1	33.3	11.8	1.7
XPS (at %) (surface)	27.4	51.5	20.9	0.22
XPS (wt %) (depth, 40 nm)	68.2	13.6	15.6	2.6
XPS (at %) (depth, 40 nm)	41.9	25.0	32.8	0.40
SEM–EDXS (wt %)	72.0	15.5	11.3	1.1
SEM–EDXS (at %)	45.7	29.5	24.6	0.2
STEM–EDXS (wt %)	75.4	12.1	12.5	
STEM–EDXS (at %)	48.9	23.4	27.7	

**Figure 4:**
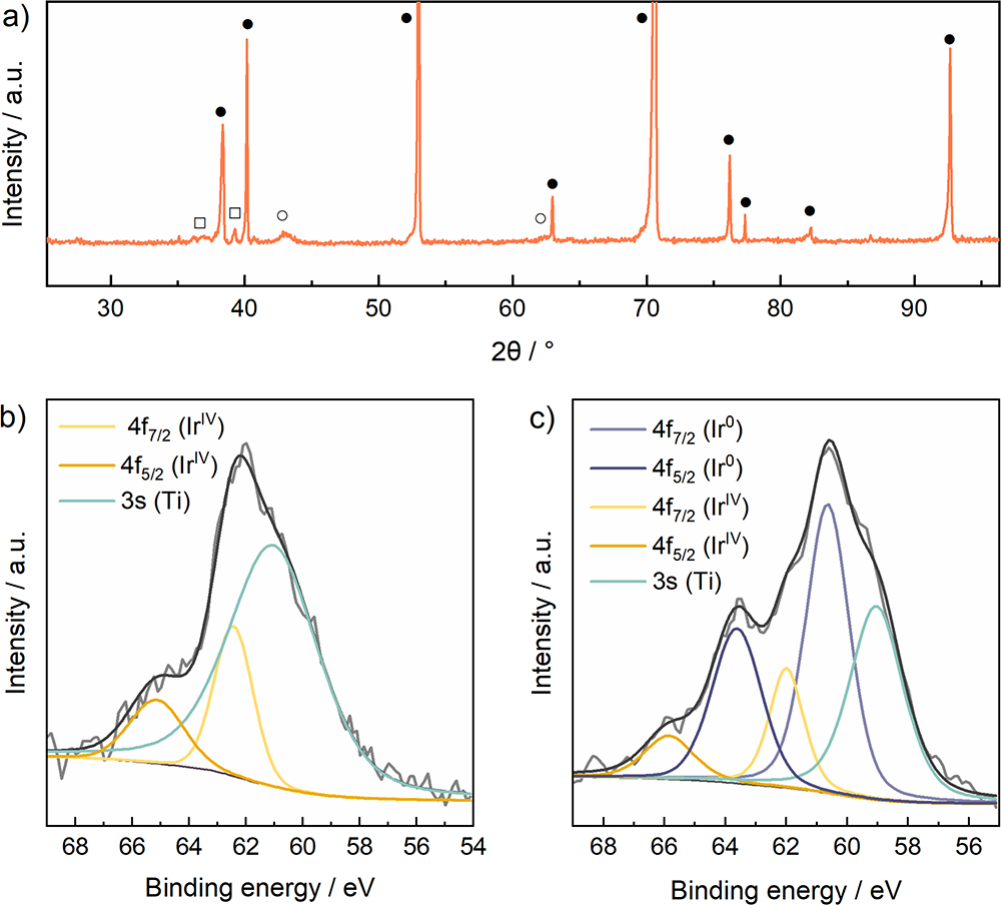
(a) XRD diffractogram of the TiO_*x*_N_*y*_–Ir catalyst showing peaks (solid circle) related to the hexagonal metal titanium foil substrate and peaks related to the tetragonal titanium nitride (Ti_2_N) (open square) and cubic titanium oxide nitride (open circle). XPS analysis of the TiO_*x*_N_*y*_–Ir sample showing the Ir 4f spectra (b) from the surface and (c) at the depth of approximately 40 nm.

The XRD diffractogram of TiO_*x*_N_*y*_–Ir (Figure 4a) shows diffraction peaks related to tetragonal titanium nitride (Ti_2_N) (open square) at 2θ = 36.3° (200) and 39.3° (111) angles (PDF 01-085-8809),^41^ and cubic titanium oxide nitride (open circle) at 43.1° (200) angle (PDF 01-084-4872).^41^ It also shows distinct peaks (solid circle) related to the hexagonal metal titanium foil substrate due to the very small thickness of the TiO_*x*_N_*y*_–Ir film. Interestingly, the titanium foil peaks in Figure 4a are significantly lowered if compared to the titanium foil peaks observed in the case of all synthetic intermediates of TiO_*x*_N_*y*_–Ir preparation. A detailed description of these diffractograms is available in the Supporting Information. Due to very low Ir loading, no diffractogram shows peaks related to Ir phases.

XPS analysis was performed to determine the composition (results shown in Table 1) and the oxidation states of the elements in the TiO_*x*_N_*y*_–Ir film. A high-energy resolution XPS spectrum of the TiO_*x*_N_*y*_–Ir sample was also recorded at the depth of approximately 40 nm after the XPS depth profile measurement. It has to be noted that the XPS spectrum of Ir 4f is overlapping with the Ti 3s spectrum, which is why the precise binding energy of the Ir 4f_7/2_ peak is difficult to measure and the oxidation state of Ir is difficult to determine. Figure 4b,c shows the XPS spectra of Ir 4f on the surface and at the depth of approximately 40 nm of the TiO_*x*_N_*y*_–Ir sample, whereas the surface and subsurface spectra of Ti 2p, O 1s, and N 1 s are shown in Figure S26. The XPS analysis on the surface of the TiO_*x*_N_*y*_–Ir sample shows that Ir is mainly oxidized having the 4f_7/2_ peak at 62.2 eV and Ti is mainly in the form of TiO_2_. At the depth of approximately 40 nm, Ir is mainly in the metallic form Ir(0) as shown in Figure 4c. In the subsurface region, Ti forms mainly Ti–N (63%) and Ti–NO bonds, and to a smaller extent, it is present as Ti(IV) related to TiO_2_ species. The depth profile of the elements in TiO_*x*_N_*y*_–Ir was determined with the XPS depth profile measured from the surface to the depth of approximately 40 nm (Figure S27). In the depth profile measurement, only Ti, O, and N elements were considered since the concentration of Ir was too low. Due to the low sensitivity of the XPS depth profile, also the time-of-flight secondary ion mass spectrometry (ToF-SIMS) depth profile was measured from the surface to the depth of approximately 400 nm (Figure S28). The ToF-SIMS depth profile shows that Ir is distributed throughout the entire thickness of the TiO_*x*_N_*y*_ film.

XANES spectra of TiO_*x*_N_*y*_–Ir before and after electrochemical activity measurement were acquired and compared to the spectra of Ti–Ir_200 as well as IrO_2_ and Ir foil reference materials (Figure S29). Showing the intense white line typical of Ir edge, the XANES spectra of Ti–Ir_200, TiO_*x*_N_*y*_–Ir, and TiO_*x*_N_*y*_–Ir after the electrochemical activity test have been compared to get information regarding the oxidation state of Ir. The spectra of pristine Ti–Ir_200 resemble those of Ir foil, suggesting that Ir is present in the zero-valent form. A very slight oxidation is observed for TiO_*x*_N_*y*_–Ir, whereas the electrochemical activity test results in partial oxidation of Ir, as depicted by the increase in intensity of the white line (Figure S29). The same conclusion was reached after analyzing the second derivative of the XANES spectra, which showed a shift to high energy (Figure S30).

### 3.3 Analysis of Electrochemical Activity

Based on our experiments described in Section 2.1, the following rationale for electrochemical performance-determining parameters can be drawn. First, an approximately 200 nm-thick Ti–Ir substrate has to be used for anodization to produce the TiO_*x*_N_*y*_–Ir film exhibiting the highest OER mass activity (Figure S11). Second, the final product of the synthesis, the TiO_*x*_N_*y*_–Ir film, is the only one exhibiting high OER electrocatalytic activity in acid medium (Figure S31). The synthetic intermediates of its preparation exhibit only incremental activity due to (i) low surface area and accessibility of Ir (mainly Ti–Ir alloy) and (ii) low electronic conductivity (amorphous and anatase TiO_2_–Ir). Third, at least three anodization parameters (anodization voltage, anodization time, and anodization electrolyte age, which is defined by the total duration of anodizations performed with the same electrolyte) significantly influence the corresponding OER activity. The optimized anodization voltage is 60 V (Figure S3), the optimized anodization time is 3 to 8 min (Figure S2), and the optimized anodization electrolyte is not used for more than approximately 255 min as can be seen from the comparison of samples prepared with an anodization electrolyte that has been used for 5, 25, 170, and 255 min (Figure S32). It has to be noted that the use of a completely fresh anodization electrolyte reduces the upper limit of the optimal anodization time to 5 min. When the anodization is performed at voltages higher than 60 V, the film morphology and composition are significantly changed (Figures S33 and S34 and Table S6) and the electrochemical activity is close to zero. Fourth, since anodized and air-annealed Ti–Ir films are very thin, the optimal nitridation time at 700 °C is only 15 min. However, the ammonia flow rate is increased to 300 cm^3^ min^–1^ to enable a high enough N/O ratio. Nitridation for 5 min results in too low N/O ratio.

**Figure 5:**
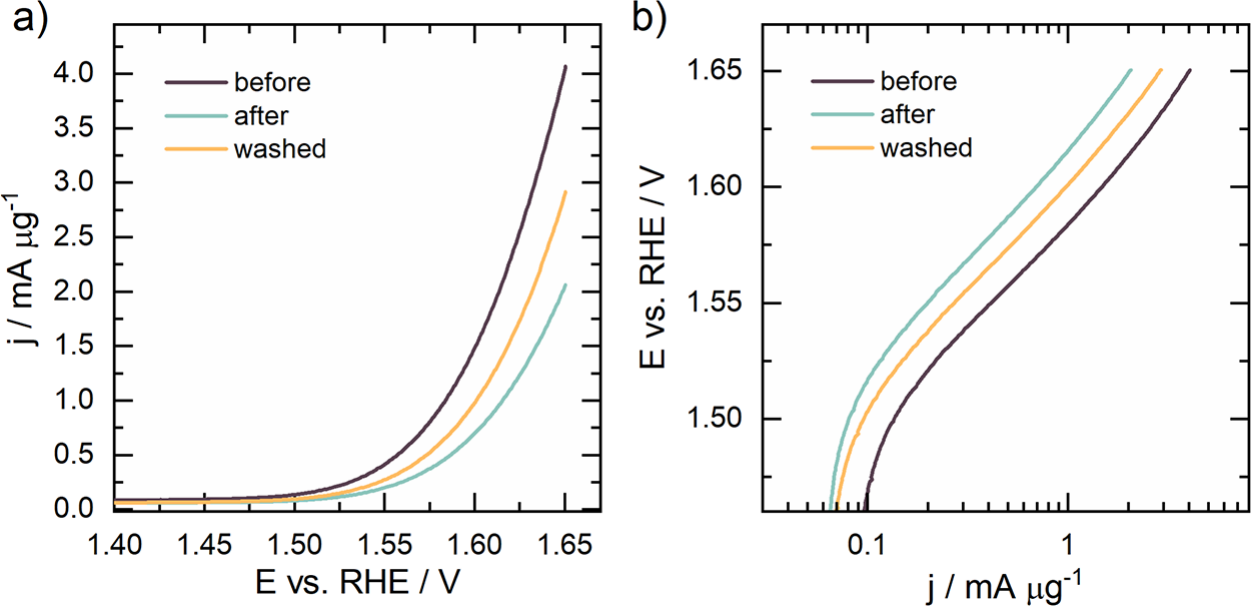
(a) OER performance of the TiO_*x*_N_*y*_–Ir catalyst before (purple line) and after (blue line) prolonged electrochemical cycling (ST-CV). After measuring the electrocatalytic activity after the electrochemical cycling test, the electrolyte was removed to remove oxygen bubbles and then the new electrolyte was added after which the activity was measured again (orange line). All the OER polarization curves are normalized per unit mass of iridium. (b) Tafel plot of OER polarization curves (constructed from panel a). Tafel slope values in the range of 89–95 mV dec^–1^ were determined.

To place the optimized TiO_*x*_N_*y*_–Ir in the context of state-of-the-art OER, the electrocatalytic performance was determined by LSV measurement. The polarization curve before (purple line) and after (blue line) prolonged electrochemical cycling (ST-CV) is shown in Figure 5a. After measuring the electrocatalytic activity following the initial degradation protocol (ST-CV), the electrolyte was replaced with a fresh one to remove oxygen bubbles that evolved during ST-CV. Subsequently, the activity was measured again (orange line).

It can be seen that an exceptionally low Ir amount of approximately 0.5 μg in the 380 nm-thick TiO_*x*_N_*y*_–Ir film (resulting in an Ir loading of 0.64 μg cm^–2^_geom._) with a specific surface area of approximately 20 ± 5 cm^2^ (see the Supporting Information for details on its determination and a comprehensive discussion on why ECSA cannot be determined for our SACC) results in mass activity values of ∼450 A g^–1^_Ir_ at 1.55 V (vs RHE) and ∼4050 A g^–1^_Ir_ at 1.65 V (vs RHE). The developed electrocatalyst greatly outperformed the IrO_2_ benchmark (Figure S11) prepared from commercial IrO_2_ (Alfa Aesar) immobilized to a glassy carbon electrode. The Ir loading in the benchmark was 51 μg cm^–2^_geom._. According to literature comparison, this represents an exceptional performance; however, an exact comparison is rather challenging, since Ir SAC OER catalysts were tested in an alkaline medium and immobilized on chemically very different supports.^22,25,31,56^ Only the study by Yin et al. reports on the acidic OER performance of NiCo_2_O_4_ nanosheet-immobilized Ir single atoms.^28^ Although highly active, the catalyst contained approximately 10.4 μg_Ir_ cm^–2^ and its high activity cannot be only attributed to Ir SA due to the content of Co, which has been proven to be active under OER conditions.^57^ The amount of Ir in all published studies is several times higher than in TiO_*x*_N_*y*_–Ir developed in this study. Our catalyst has even an order of magnitude lower amount of Ir than required for TW scale-up of the proton-exchange membrane water electrolyzer (0.05 mg_Ir_ cm^–2^).^58^ In this regard, the OER activity of TiO_*x*_N_*y*_–Ir is comparable to previously reported values of Ir-based electrocatalysts for acid OER (see Table S7 in the Supporting Information). Therefore, the overall literature comparison strongly suggests that the high mass activity of TiO_*x*_N_*y*_–Ir should be attributed to the high Ir atom utilization efficiency. Further insights, obtained via Tafel analysis, reveal comparable Tafel slope values (89–95 mV dec^–1^, Figure 5b) to the IrO_2_ benchmark (95 mV dec^–1^, Figure S11b), which is slightly higher than typically measured for metallic Ir-based analogues. In the latter case, values between 40 and 60 mV dec^–1^ are obtained,^21,59−61^ which is in line with the second OER step (deprotonation of OH_ad_ leading to the formation of adsorbed oxygen, O_ad_) being the rate-determining step.^62^ The deviation of Tafel slopes for TiO_*x*_N_*y*_–Ir SACC indicates that perhaps not only a single OER mechanism is operating, which however is outside the scope of the current study.^63^ Further characterization was performed by electrochemical impedance spectroscopy measurements (Figure S35) in the OER region (1.6 V) from where the charge transfer resistance (*R*_ct_) of 83.1 Ω was determined. Note that this value is approximately 5-fold higher if adequately compared to a commercial Ir OER composite (see text below Figure S35 for a detailed discussion), which should be ascribed to ultralow iridium loading in the TiO_*x*_N_*y*_–Ir SACC.

Although initially highly active, the performance of TiO_*x*_N_*y*_–Ir declined (Figure 5a, orange line) to approximately 72% of initial OER activity at 1.65 V. This is in relatively good agreement with the analysis of the HClO_4_ electrolyte used for ST-CV test where approximately 70 ng of Ir was determined with ICP-MS analysis. A total of 13% of Ir is lost due to Ir SACC detachment during electrochemical degradation in the TiO_*x*_N_*y*_–Ir catalyst, which is most likely the consequence of the loss of TiO_*x*_N_*y*_–Ir support contact due to minor transient dissolution of TiO_*x*_N_*y*_ during potentiodynamic treatment as observed recently.^64^ The possible solution for an even stronger decrease in electrocatalytic activity was observed before replacing the electrolyte with a fresh one due to the accumulation of oxygen bubbles on the electrode surface. This result implies that the electrochemical cell design (Figure S2) for electrocatalytic stability assessments still requires further improvements, compatibly with the need to perform anodic oxidation of foil substrates.

We observed no detrimental decrease in N/O ratio to result in the decreased electronic conductivity of our SACC catalyst, whereas the Ir amount in the catalyst determined by SEM–EDXS stays the same (Table 2). Last but not least, the SEM analysis of the TiO_*x*_N_*y*_–Ir catalyst before and after the electrocatalytic activity test as well as after the electrocatalytic stability test shows no significant morphological changes to the top surface of the TiO_*x*_N_*y*_–Ir film (Figure S36).

**Table 2:** Chemical Composition of the TiO_*x*_N_*y*_–Ir Catalyst before and after Electrocatalytic Activity and Stability Measurements^b^

	Ti	O	N	Ir	N/O
before activity^a^	69.3	16.8	12.5	1.0	0.85
after activity	68.0	20.5	10.5	1.0	0.59
after ST-CV stability	68.8	20.7	9.8	0.8	0.54

a 0.3 wt % F has also been detected.

b Composition was determined with SEM–EDXS, and it is shown in wt %. The N/O ratio was calculated from at %.

## 4 Conclusions

In summary, a novel approach for the preparation of thin-film electrode composites has been introduced. STEM and XRD characterizations reveal that α-Ti solid solution has been successfully prepared with triode sputtering and later transformed with anodic oxidation and heat treatments to the TiO_*x*_N_*y*_–Ir film with Ir single atoms and clusters. To our best knowledge, the preparation of α-Ti solid solution with triode sputtering and the anodization of so-prepared alloys have not been described before. SEM–EDXS and ToF-SIMS characterizations further reveal that Ir is distributed throughout the entire film thickness, whereas XPS analysis shows that Ir in the subsurface region of the catalyst is mainly in the metallic form Ir(0), whereas the supporting TiO_*x*_N_*y*_ structure consists of Ti nitride, oxynitride, and to a smaller extent, Ti oxide. This support composition gives the catalyst good electronic conductivity, whereas the high surface area is ensured during the anodization process and etching of the Ti–Ir alloy with fluoride ions. The developed TiO_*x*_N_*y*_–Ir SACC catalyst exhibits a high mass electrocatalytic activity for OER in acid, although a record-low Ir amount never reported for the Ir-based OER catalysts before was used. This result suggests a very high Ir atom utilization efficiency of the novel catalyst. Furthermore, no morphological changes of the support and a low decrease in N/O ratio were observed after electrochemical measurements.

Overall, the successful preparation of TiO_*x*_N_*y*_–Ir catalysts with the simultaneous sputtering of Ti and Ir, the anodization of the Ti–Ir alloy, and the two heat treatments is promising since it could be the basis for the incorporation of a number of other catalytic metals (Pt, Co, Pd, etc.) into Ti and other transition metals that can be anodized (e.g., W, Ni, V, Fe, etc.) to form metal cluster- and single atom-incorporated high-surface-area nanostructures for various electrocatalytic and photocatalytic reactions. In other words, anodization and post-treatment of solid solutions could represent a new way of preparation of catalysts with minimal content and very high atom utilization efficiency of rare and expensive metals, thereby offering an alternative to existing technologies of catalyst preparation.

## Supporting Information Available

Dimensions of sputtering target, electrochemical cell design, FIB lamella, topography of worn target, XANES spectra, impedance measurement, additional electrochemical, STEM, XRD, SEM, EDXS, XPS, and ToF-SIMS results, and discussion on ECSA and specific surface area determination (PDF)
