# Supplementary material for: Nanotubular TiOxNy-Supported Ir Single Atoms and Clusters as Thin-Film Electrocatalysts for Oxygen Evolution in Acid Media
Source: Chem Mater. 2023 Mar 9;35(6):2612–23. doi: 10.1021/acs.chemmater.3c00125 (PMC10061659; doi:10.1021/acs.chemmater.3c00125)
Supplement: Supplementary file 1 — cm3c00125_si_001.pdf [file cm3c00125_si_001.pdf]

*Supplementary information*

# Nanotubular $TiO_xN_y$-supported Ir Single Atoms and Clusters as Thin Film Electrocatalysts for Oxygen Evolution in Acid Media

**Luka Suhadolnik,[1,*] Marjan Bele,[2] Miha Čekada,[3] Primož Jovanovič,[2] Nik Maselj,[2,4] Anja Lončar,[2,5] Goran Dražić,[2] Martin Šala,[6] Nejc Hodnik,[2,5,7] Janez Kovač,[8] Tiziano Montini,[1] Michele Melchionna,[1,*] Paolo Fornasiero[1,*]**

[1] Department of Chemical and Pharmaceutical Sciences, University of Trieste, via L. Giorgieri 1, 34127 Trieste, Italy

[2] Department of Materials Chemistry, National Institute of Chemistry, Hajdrihova 19, SI-1000 Ljubljana, Slovenia

[3] Department of Thin Films and Surfaces, Jožef Stefan Institute, Jamova 39, SI-1000 Ljubljana, Slovenia

[4] Faculty of Chemistry and Chemical Technology, University of Ljubljana, Večna pot 113, SI-1000 Ljubljana, Ljubljana

[5] University of Nova Gorica, Vipavska 13, SI-5000 Nova Gorica, Slovenia

[6] Department of Analytical Chemistry, National Institute of Chemistry, Hajdrihova 19, SI-1000 Ljubljana, Slovenia

[7] Jožef Stefan International Postgraduate School, Jamova 39, SI-1000 Ljubljana, Slovenia

[8] Department of Surface Engineering, Jožef Stefan Institute, Jamova 39, SI-1000 Ljubljana, Slovenia

* Corresponding authors: Dr. Luka Suhadolnik, email: luka.suhadolnik@units.it
Dr. Michele Melchionna, email: melchionnam@units.it
Prof. Dr. Paolo Fornasiero, email: pfornasiero@units.it

**Figures:**

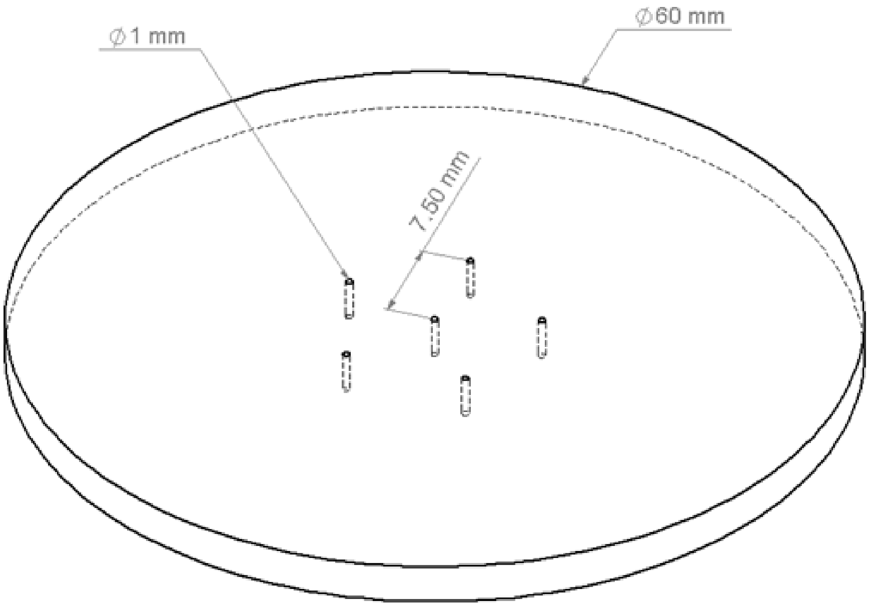


**Figure S1:** Dimensions of Ti sputtering target and positions of Ir wires inserted into the target.

The sputtering target was prepared from Ti target into which 6 holes were drilled and 1 mm Ir wire was inserted. The exact positions of the Ir wires are shown in **Figure S1**.

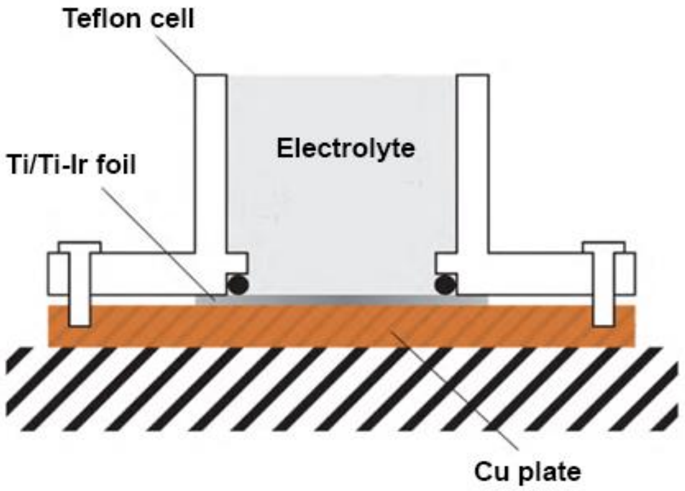


**Figure S2:** Electrochemical cell design that was used for anodization (2-electrode configuration) and electrochemical measurements (3-electrode configuration).

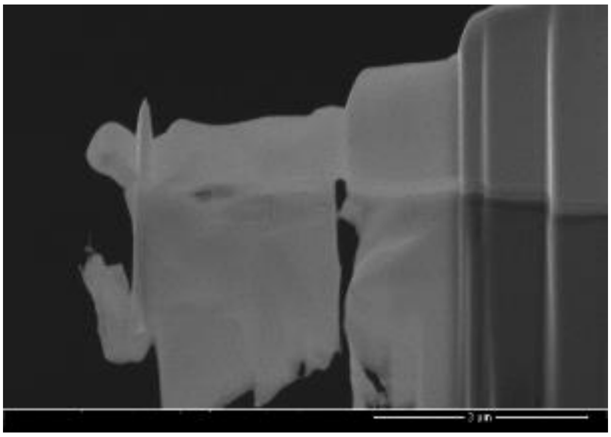

**Figure S3:** FIB lamella of Ti–Ir film on Ti substrate.

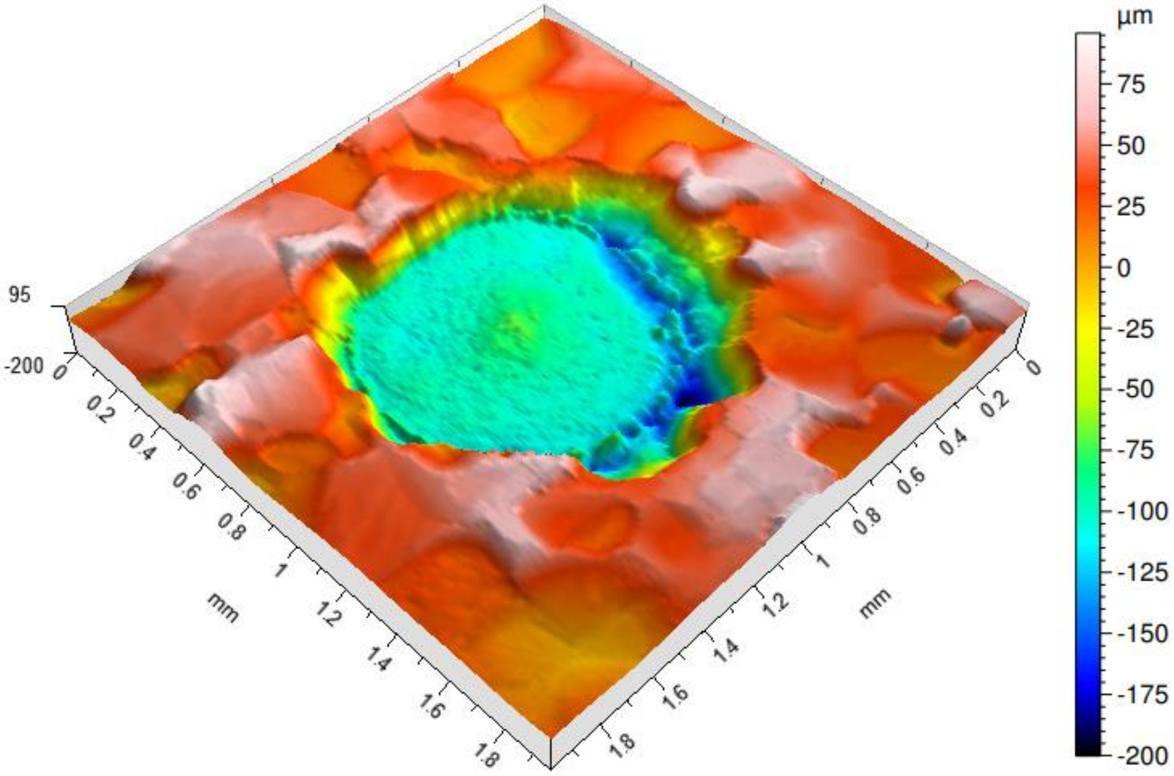


**Figure S4:** Topography of the worn target at the iridium inserts, which are about 150 µm deeper than the surrounding titanium base.

After the depositions we evaluated the profile of the sputtered area of the target. The depth of the worn area was determined by focusing selected points on the target under an optical microscope. The crater size was found to have a diameter of about 40 mm and a maximum depth of 0.47 mm. We took a closer look at the iridium inserts in the target. Before depositions they were levelled to the virgin titanium target. After the depositions we made a profiler scan of the area around the iridium inserts. We found out that the height at the iridium insert was around 150 µm lower than the height of the neighboring titanium base target (**Figure S4**). This

is a consequence of increased sputtering rate of iridium compared to titanium. From these geometries we were able to calculate the volume of the consumed titanium and iridium, and out of this the predicted composition of the film. The obtained result of 1.0 at.% Ir is close to the results given by the other methods.

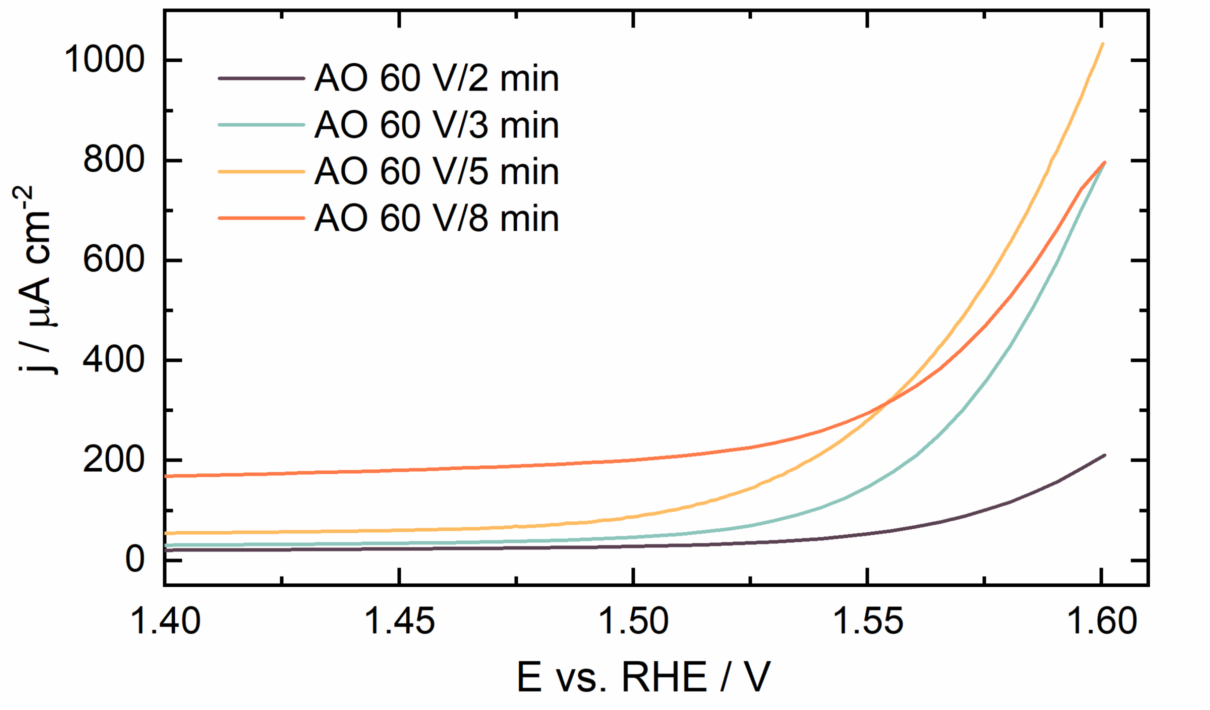


**Figure S5:** OER polarization curves of the catalysts prepared from app. 200 nm-thick Ti–Ir film anodized at 60 V for different time: 2 min (*purple* line), 3 min (*blue* line), 5 min (*orange* line), and 8 min (*red* line). After anodization all samples were annealed at 450 °C in air for 1 h and at 700 °C in $NH_3$ for 15 min.

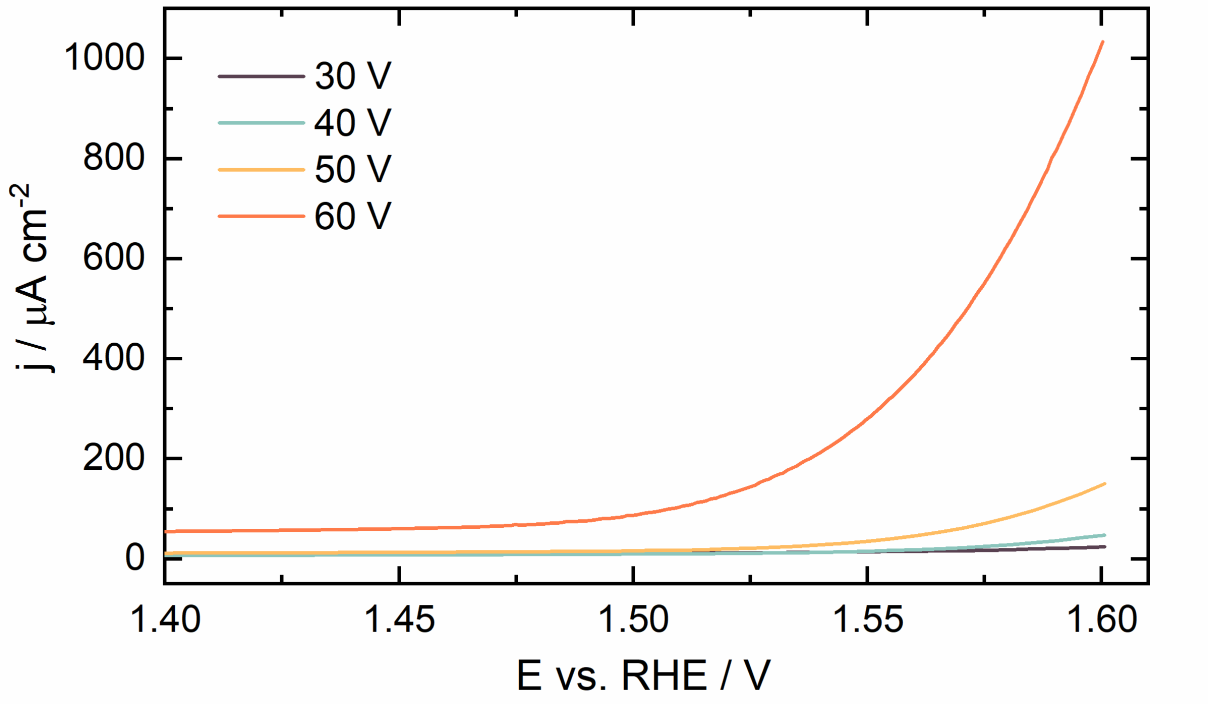


**Figure S6:** OER polarization curves of the catalysts prepared from app. 200 nm-thick Ti–Ir film anodized for 5 min at different voltages: 30 V (*purple* line), 40 V (*blue* line), 50 V (*orange* line), and 60 V (*red* line). After anodization all samples were annealed at 450 °C in air for 1 h and at 700 °C in $NH_3$ for 15 min.

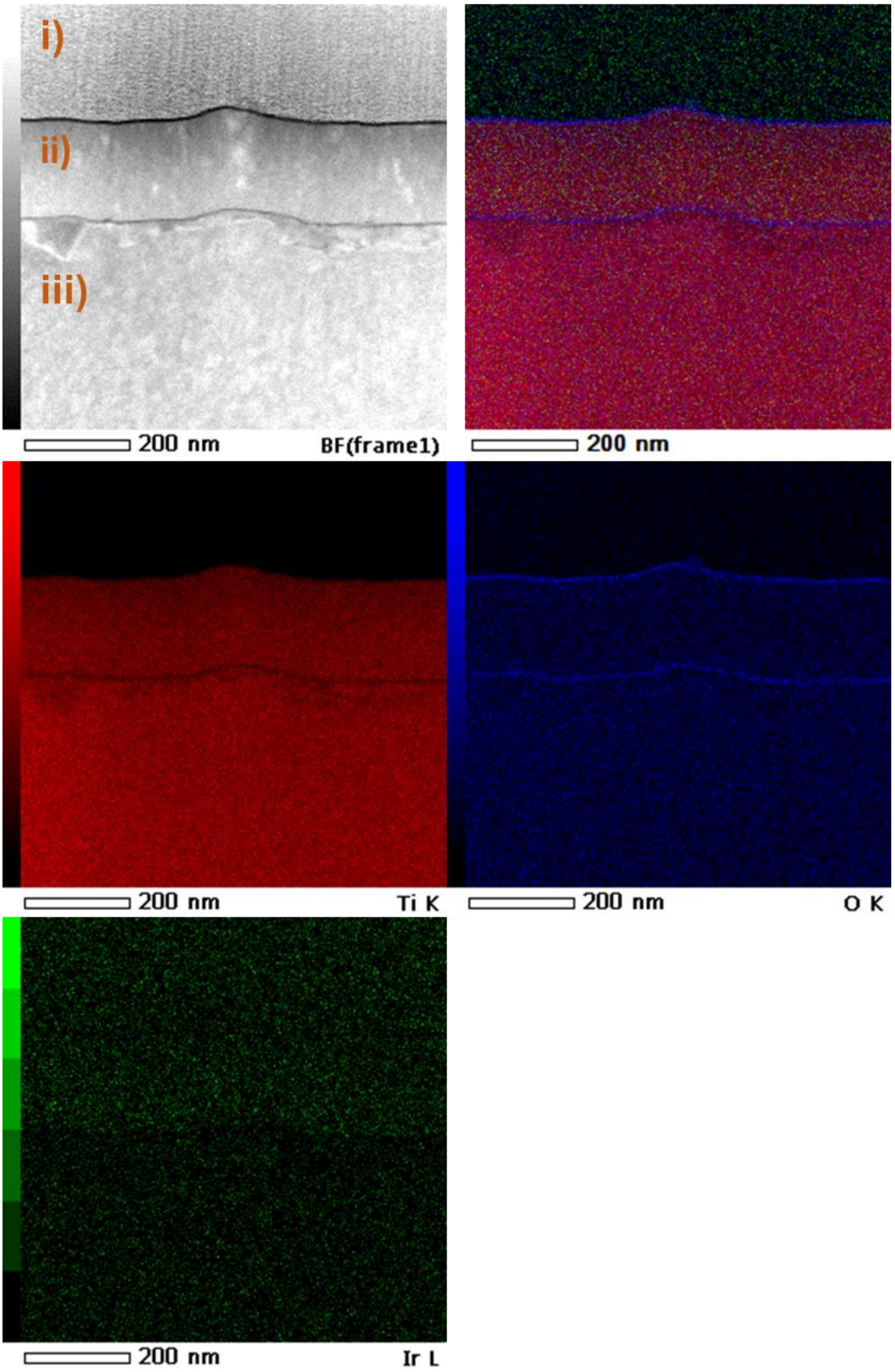


**Figure S7:** STEM energy dispersive X-ray spectroscopy (EDXS) elemental maps of Ti, O, and Ir in the cross-section of Ti–Ir film (ii). Ir signal from the Ti substrate (iii) and Pt protection layer (i) comes due to the overlay of Ir and Pt peaks and the contamination of the sample with Pt during FIB lamella preparation.

The analysis of Ti–Ir lamella with EDXS is problematic due to the presence of Pt and Cu which have similar peak positions as Ir. Nevertheless, according to Ti–Ir phase diagram, the measured Ir content in the sputtered Ti–Ir alloy (**Table S1**) corresponds to the formation of α-Ti solid solution[1] which has single Ir atoms incorporated in the Ti structure. α-Ti may contain up to 1 at.% of Ir. The oxygen present in the Ti–Ir alloy is due to surface oxidation and oxidation during sputtering of Ti that has a high affinity to oxygen.

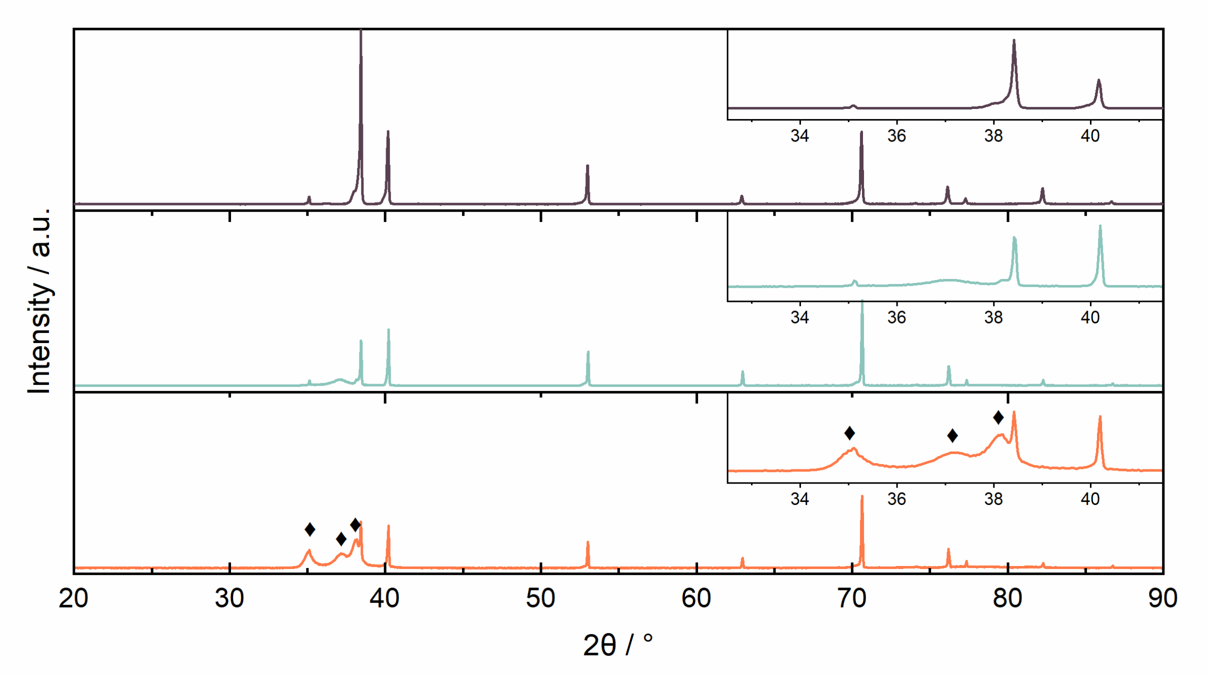


**Figure S8:** XRD diffractogram of (top) Ti foil, (middle) app. 200-nm-thick and (bottom) app. 1000-nm-thick Ti–Ir alloy sputtered on the Ti foil substrate. Due to the very low thickness of the 200-nm- and 1000-nm-thick films, the insets show a close-up of the most important regions in each sample. The peaks related to sputtered Ti–Ir alloy are denoted with ♦.

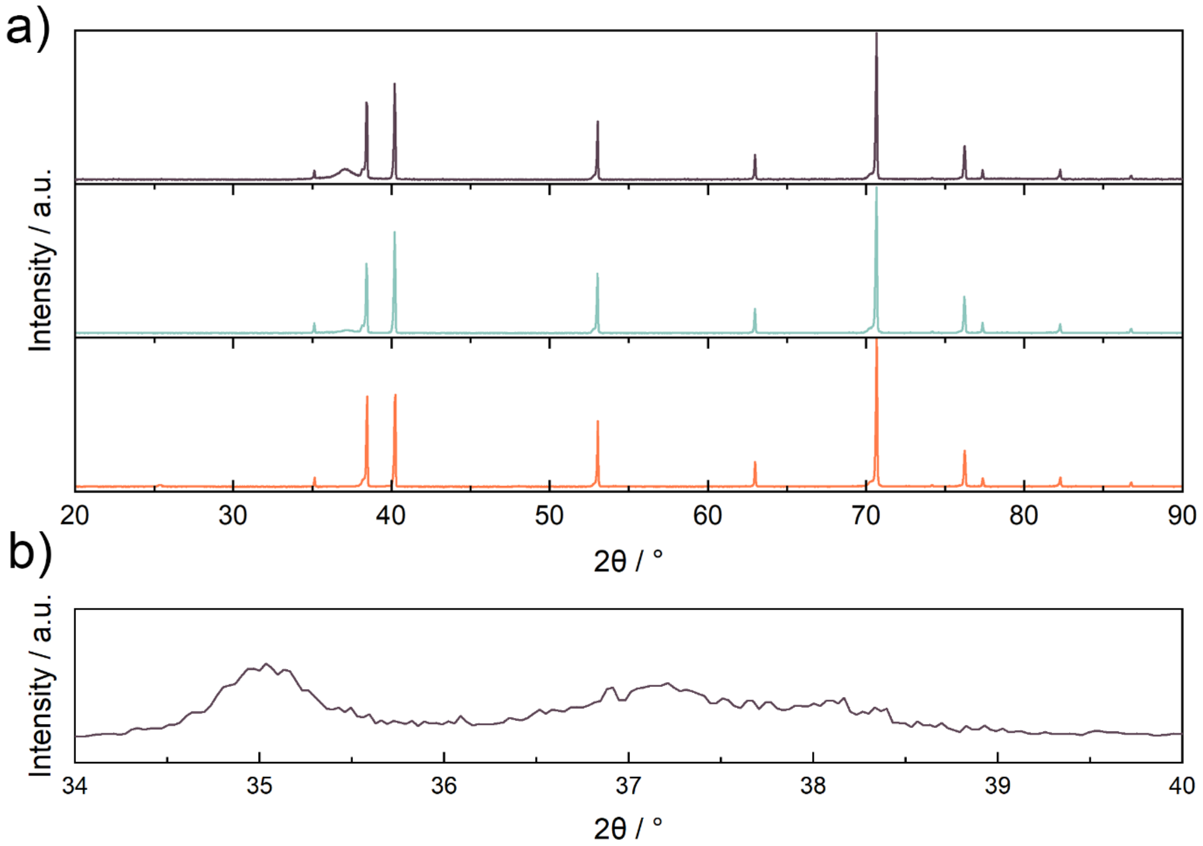


**Figure S9:** (a) XRD diffractograms of synthetic intermediates of $TiO_xN_y$–Ir catalyst preparation: (top) app. 200-nm-thick Ti–Ir alloy on the Ti foil substrate, (middle) anodized Ti–Ir alloy, and (bottom) air-annealed anodized Ti–Ir alloy. (b) XRD diffractogram of Ti–Ir alloy sputtered on Si substrate.

XRD diffractograms of Ti foil, app. 200-nm-thick and app. 1000-nm-thick Ti–Ir alloy sputtered on the Ti foil substrate are shown in **Figure S8.** All diffractograms show distinct peaks related to hexagonal metal titanium foil substrate at 2θ = 35.1° (100), 38.5° (002), 40.2° (101), 53.1° (102), 62.9° (110), 70.8° (103), 76.2° (112), 77.4° (201), 82.5° (004), 86.8° (202) and 92.9° (104) angles (PDF 04-003-2226)[2]. Both samples with Ti–Ir alloy show three additional peaks (labeled with ♦ in **Figure S8** bottom) that are related to sputtered α-Ti solid solution with very small grain size. These three peaks were more precisely identified by the XRD analysis of the app. 1000-nm-thick Ti–Ir film sputtered on Si substrate shown in **Figure S9b**. The additional broad diffraction peaks (♦) appear at 2θ = 35.0°, 37.2° and 38.2° angles. The XRD analysis is in line with the information obtained with XPS, EDXS and STEM therefore confirming the presence of α-Ti solid solution with Ir single atoms incorporated into the Ti lattice. There is no intermetallic phase $Ti_3Ir$. According to available thermodynamic data on Ti–Ir phase diagram[1] α-Ti solid solution has Pearson symbol hP2 and space group P63/mmc. The three peaks related to α-Ti solid solution almost completely disappear after the anodization of the alloy when amorphous $TiO_2$–Ir is formed and the Ti–Ir alloy is oxidized (**Figure S9a, middle**). Due to the amorphous nature of the grown $TiO_2$–Ir oxide, in this stage of the sample preparation only the

XRD diffraction peaks corresponding to the metal titanium substrate are observed. Annealing in air transforms the amorphous film to anatase $TiO_2$–Ir (**Figure S9a, bottom**), whereas annealing in ammonia transforms the latter to the $TiO_xN_y$–Ir (**Figure 9a**). Only the most characteristic XRD diffraction peaks related to these chemical structures are visible due to too low film thickness and the relatively large peaks of titanium substrate. However, the observed changes in the film structure are supported with our recent studies on $TiO_xN_y$–Ir materials prepared differently[2] and with EDXS, XPS, and STEM performed in this study on $TiO_xN_y$–Ir.

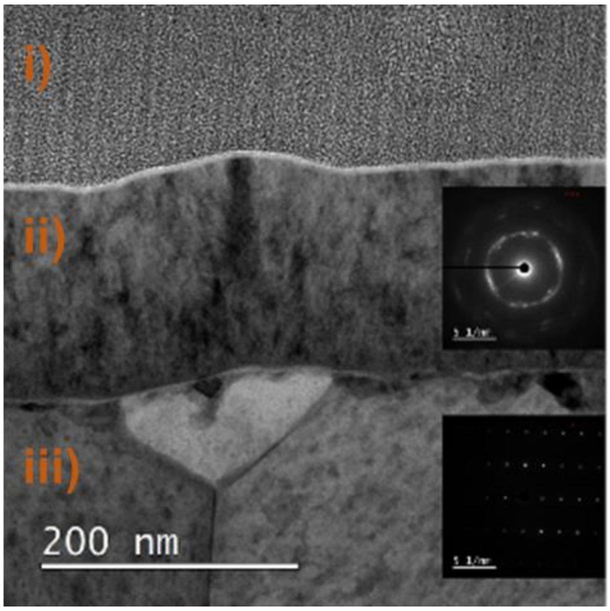


**Figure S10:** STEM analysis of cross-sectioned FIB lamella (**Figure S3**) of Ti–Ir film (ii) on Ti metal substrate (iii). Low magnification STEM-BF image of Ti–Ir film also includes the electron diffraction patterns of Ti–Ir film and Ti foil substrate. (i) is a C protection layer that was used to protect the film during the FIB lamella preparation.

STEM analysis of Ti–Ir lamella reveals the columnar growth of the film which is app. 180 nm thick (**Figure S10)**. The STEM micrograph with the electron diffraction patterns of Ti–Ir film and Ti foil substrate additionally reveals that the Ti–Ir film is textured which is the most evident from the arc observed at two locations in its diffraction pattern. As shown in **Figure S10** there is no arc in the diffraction pattern of Ti substrate. The STEM and EDXS mapping (**Figure S7**) analyses additionally confirm the sputtered film has no $Ti_3Ir$ phase and therefore constitutes of solely Ir single atoms in the Ti lattice. As observed in the STEM image (**Figure S10)** the Ti grain size in the Ti–Ir film is far smaller than in the underlying Ti substrate. The grain size area of the substrate is between 10 and 30 $\mu m^2$,[3] whereas the diameter of the Ti grains in the Ti–Ir film is in the range of 10 to 30 nm as determined with STEM. This is in line with XRD analysis of the Ti–Ir film on Ti foil substrate where the sputtered titanium solid solution with small grains is shown as three additional peaks in the XRD pattern of the underlying Ti foil substrate (**Figure S8**). The EDXS map (**Figure S7)** also shows the thin oxidized layer at the top and the

bottom of the Ti–Ir film which occurs due to the Ti–Ir substrates storage in air and the Ti foils cleaning procedure before sputtering the alloy.

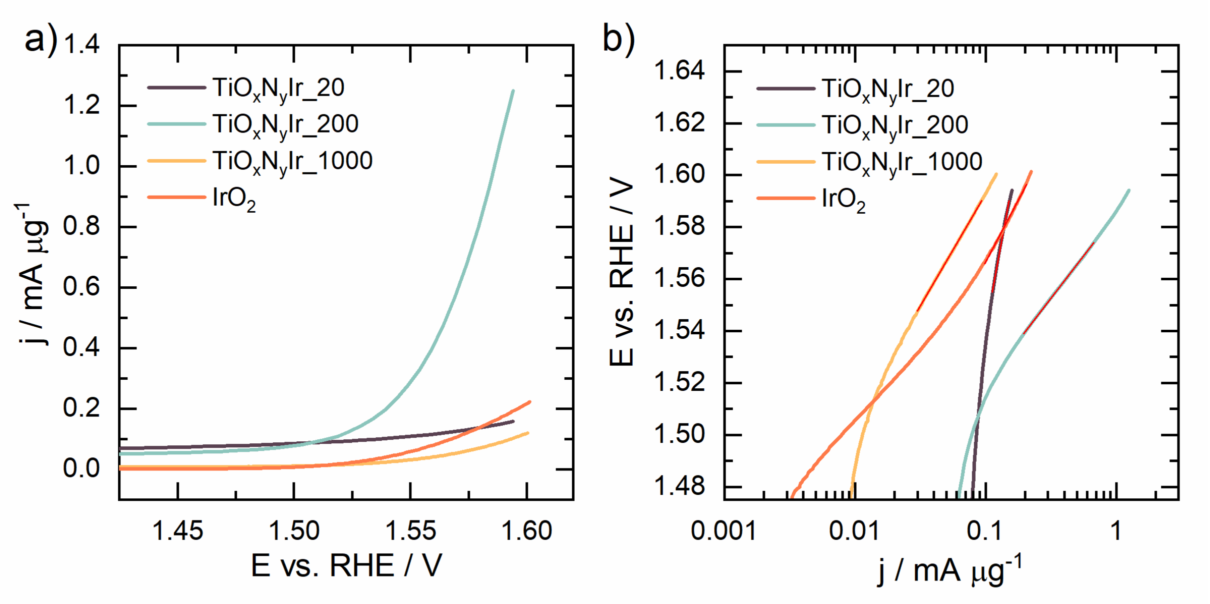


**Figure S11:** a) Electrocatalytic activity of $IrO_2$ benchmark on glassy carbon electrode *(red)* and anodized and post-treated Ti–Ir samples: 1000 nm-thick Ti–Ir film *(orange)* anodized at 60 V for 5 min, 200 nm-thick Ti–Ir film *(blue)* anodized at 60 V for 5 min, and 20 nm-thick Ti–Ir film *(purple)* anodized at 60 V for 1 min. All Ti–Ir samples were annealed at 450 °C for 1 h in air and at 700 °C for 15 min in $NH_3$ (flow rate 50 $cm^3$ $min^{-1}$). The Ir loading of 0.079 µg $cm^{-2}_{geom}$, 0.64 µg $cm^{-2}_{geom}$, 4.1 µg $cm^{-2}_{geom}$, and 51 µg $cm^{-2}_{geom}$ was determined for the $TiO_xN_y$–Ir_20, $TiO_xN_y$–Ir_200, $TiO_xN_y$–Ir_1000, and $IrO_2$ electrode, respectively. Note that a very low amount of Ir in the $TiO_xN_y$–Ir_1000 was catalytically active since it was present in the dense Ti–Ir film below the anodized film and was not available to the electrolyte solution. b) Tafel plot of OER polarization curves (constructed from a).

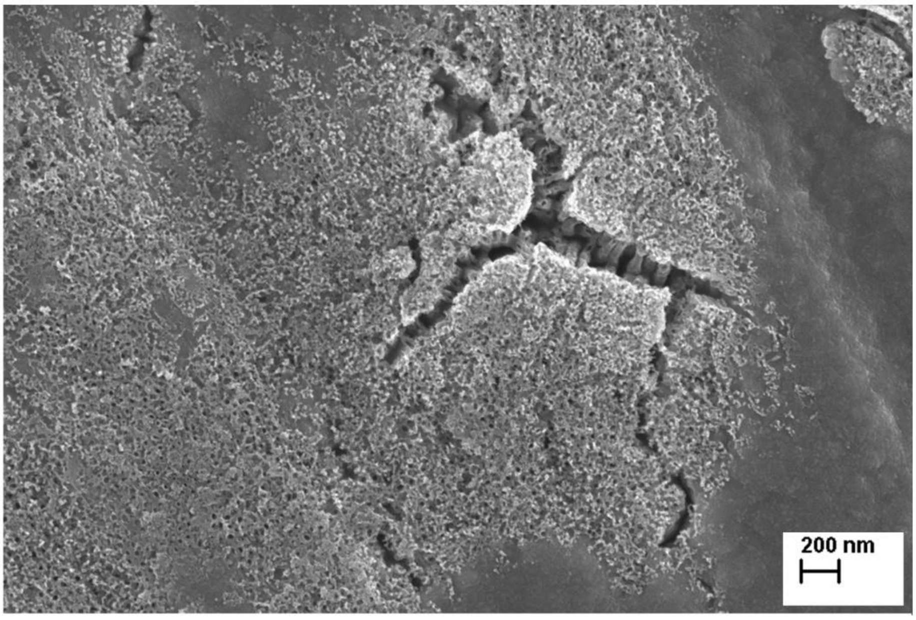


**Figure S12:** Top surface of the $TiO_xN_y$–Ir catalyst prepared with anodization of 20 nm-thick Ti–Ir film at 60 V for 1 min shows anodized and non-anodized regions.

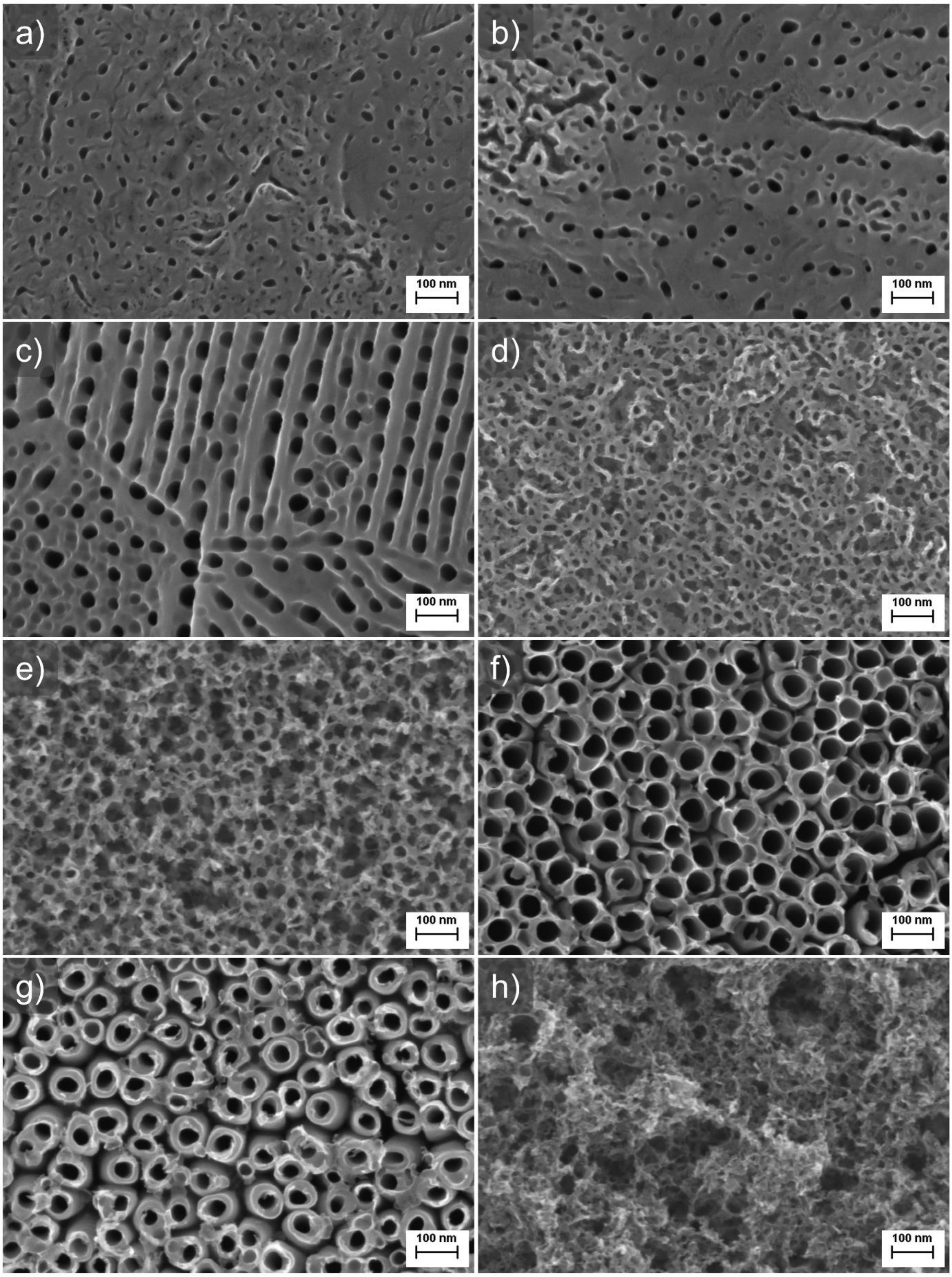


**Figure S13:** Top-view SEM images of anodized Ti and Ti–Ir films at 60 V for 15 s, 5 min and 1 h. All three Ti–Ir film thicknesses were anodized for 1 h, whereas shorter anodization times were only applied to Ti and 200-nm-thick Ti–Ir film. All the samples shown in this Figure were anodized with aged electrolyte. a) Ti anodized for 15 s, b) Ti anodized for 5 min, c) Ti anodized for 1 h, d) 200-nm-thick Ti–Ir anodized for 15 s, e) 200-nm-thick Ti–Ir anodized for 5 min, f) 20-nm-thick Ti–Ir anodized for 1 h, g) 200-nm-thick Ti–Ir anodized for 1 h, and h) 1000-nm-thick Ti–Ir anodized for 1 h.

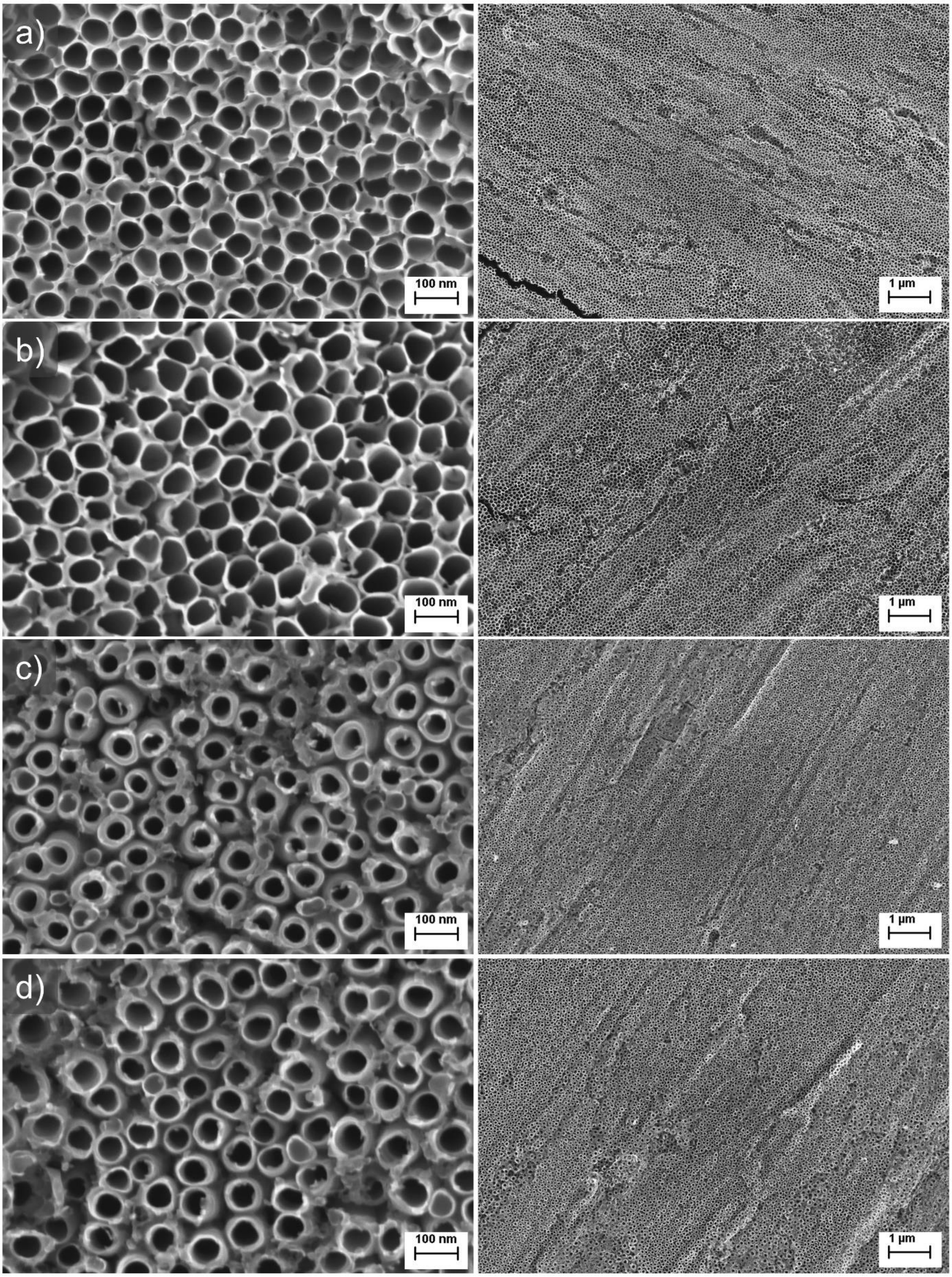
a)
100 nm
1 µm
b)
100 nm
1 µm
c)
100 nm
1 µm
d)
100 nm
1 µm

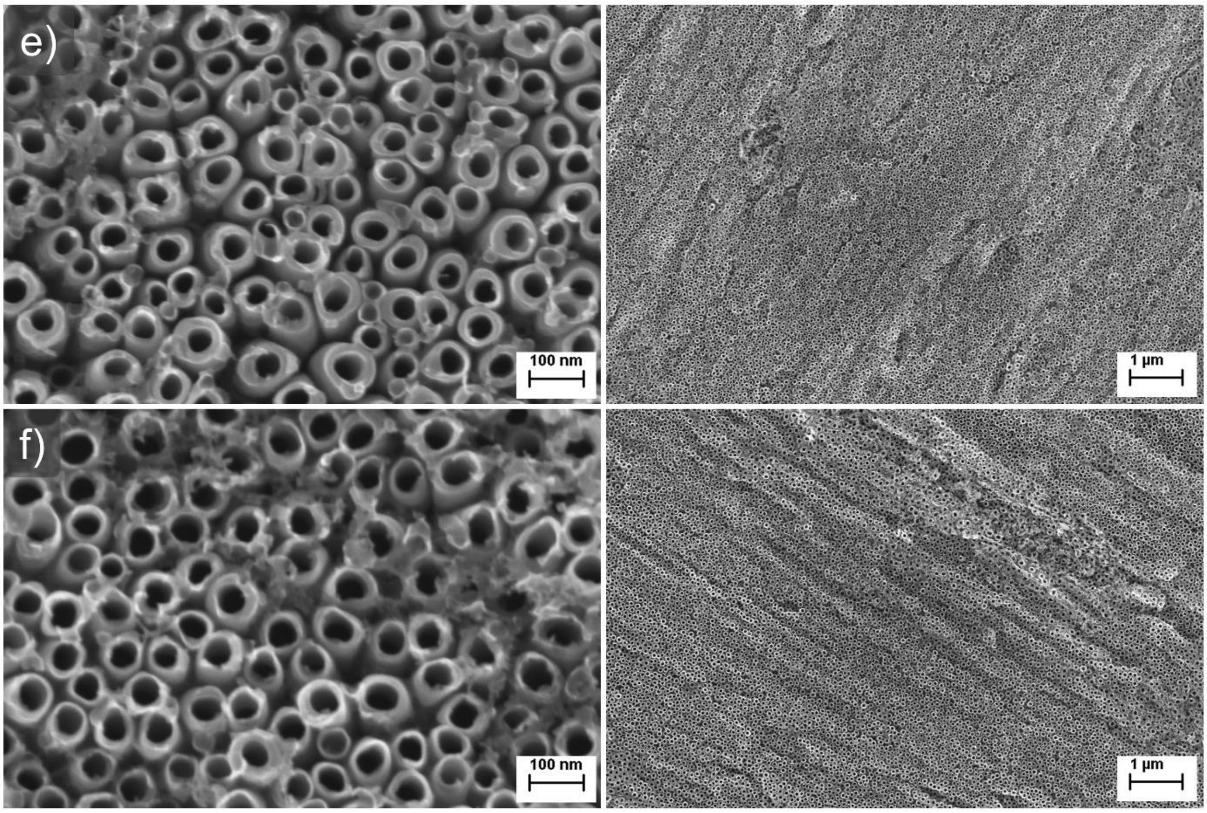


**Figure S14:** SEM micrographs of anodized Ti–Ir films of different thicknesses from a top view at lower and higher magnification. Anodizations were performed at 60 V for 3 and 5 h. a) 20-nm-thick Ti–Ir anodized for 3 h, b) 20-nm-thick Ti–Ir anodized for 5 h, c) 200-nm-thick Ti–Ir anodized for 3 h, d) 200-nm-thick Ti–Ir anodized for 5 h, e) 1000-nm-thick Ti–Ir anodized for 3 h, and f) 1000-nm-thick Ti–Ir anodized for 5 h.

**Figure S13** shows the comparison of surface morphology of anodized Ti–Ir and Ti at 60 V for 15 s, 5 min and 1 h analyzed by SEM. Ti sample anodized for 15 s shows starting compact $TiO_2$ with small pores (initiation layer, **Figure S13a**). In contrast, Ti–Ir anodized for 15 s shows a high-density porous structure (**Figure S13d**). As shown in **Figure S16**, the compact initiation layer can also be formed at the start of Ti–Ir anodization, however, it is not always observed. Ti samples anodized for 5 min (**Figure S13b**) and 1 h (**Figure S13c**) show a similar top surface morphology that has been formed at the start of Ti anodization. The main change observed with increasing Ti anodization time is the increased density and size of $TiO_2$ pores caused by fluoride ions etching. Ti–Ir sample anodized for 5 min is shown in **Figure S13e**. A less defined nanoporous morphology with some clearly visible nanotube tops is observed. Such morphology is characteristic for anodized Ti–Ir samples before ammonia annealing. Increasing the Ti–Ir anodization time to 1 h (**Figure S13f,g**) etches the entire Ti–Ir film away and results in a closely-packed (anodized app. 20-nm-thick Ti–Ir film, see **Figure S13f**) or spaced (anodized app. 200-nm-thick Ti–Ir film, see **Figure S13g**) highly ordered $TiO_2$ nanotubes if the sputtered Ti–Ir film is not too thick. Increasing the Ti–Ir film thickness to app. 1000 nm requires longer

anodization time to fully etch away the entire anodized Ti–Ir film. Anodization of app. 1000-nm-thick Ti–Ir film at 60 V for 1 h results in a nanoporous film of $TiO_2$–Ir (**Figure S13h**) that has not been completely etched away. Anodized Ti–Ir is completely etched away during the anodization of all three Ti–Ir film thicknesses if the anodization time at 60 V is 3 h or more. **Figure S14** shows different Ti–Ir samples anodized at 60 V for 3 and 5 h.

Three-hour-long anodization at 60 V leads to the complete removal of all anodized Ti–Ir in case of anodization of app. 20-nm-thick, app. 200-nm-thick and app. 1000 nm-thick Ti–Ir films (**Figure S14a, c and e**). Increasing the anodization time to 5 h decreases the nanotube wall thickness and increases the nanotube length (**Figure S14b, d and f**). Results show that the spacing between the nanotubes is not influenced by the anodization time, it is rather slightly controlled with the sputtered Ti–Ir film thickness. Spaced nanotubes are observed when the Ti–Ir film thickness is app. 200 nm or more, however, the space between the nanotubes is partially filled up with the smaller-diameter-nanotubes observed in anodized app. 1000-nm-thick Ti–Ir film (**Figure S14e and f**). Highly defined $TiO_2$ nanotube openings are observed due to prolonged anodization of sputtered Ti–Ir film during which the sputtered film is first anodized and then completely etched away due to the excessive etching of the fluoride ions. The $TiO_2$–Ir nucleation layer is formed in the Ti–Ir film instead of the underlying Ti surface thus protecting it at the start of the anodization when the nanotube tops are formed. This approach is similar to the existing strategies used to prepare highly defined $TiO_2$ nanotube openings and spaced $TiO_2$ nanotubes with the anodization of Ti. Ordered tube tops are usually prepared with a very long anodization of Ti or anodization at very high voltages which leads to thinning of the tube walls and the collapse of the top surface of the nanotubular film.[4] Another approach avoids the formation of the disordered $TiO_2$ initiation layers by coating the starting Ti surface with a photoresist that is slowly soluble in the anodization electrolyte. This eliminates the formation of undesired initiation layers and protects the nanotube tops to a certain extent from etching by the fluoride ions.[5] Controlled spacing of self-organized $TiO_2$ nanotubes can be achieved if Ti is anodized in di-ethylene glycol electrolytes with 4 wt.% HF, 0.3 wt.% $NH_4F$ and 1 wt.% water.[6]

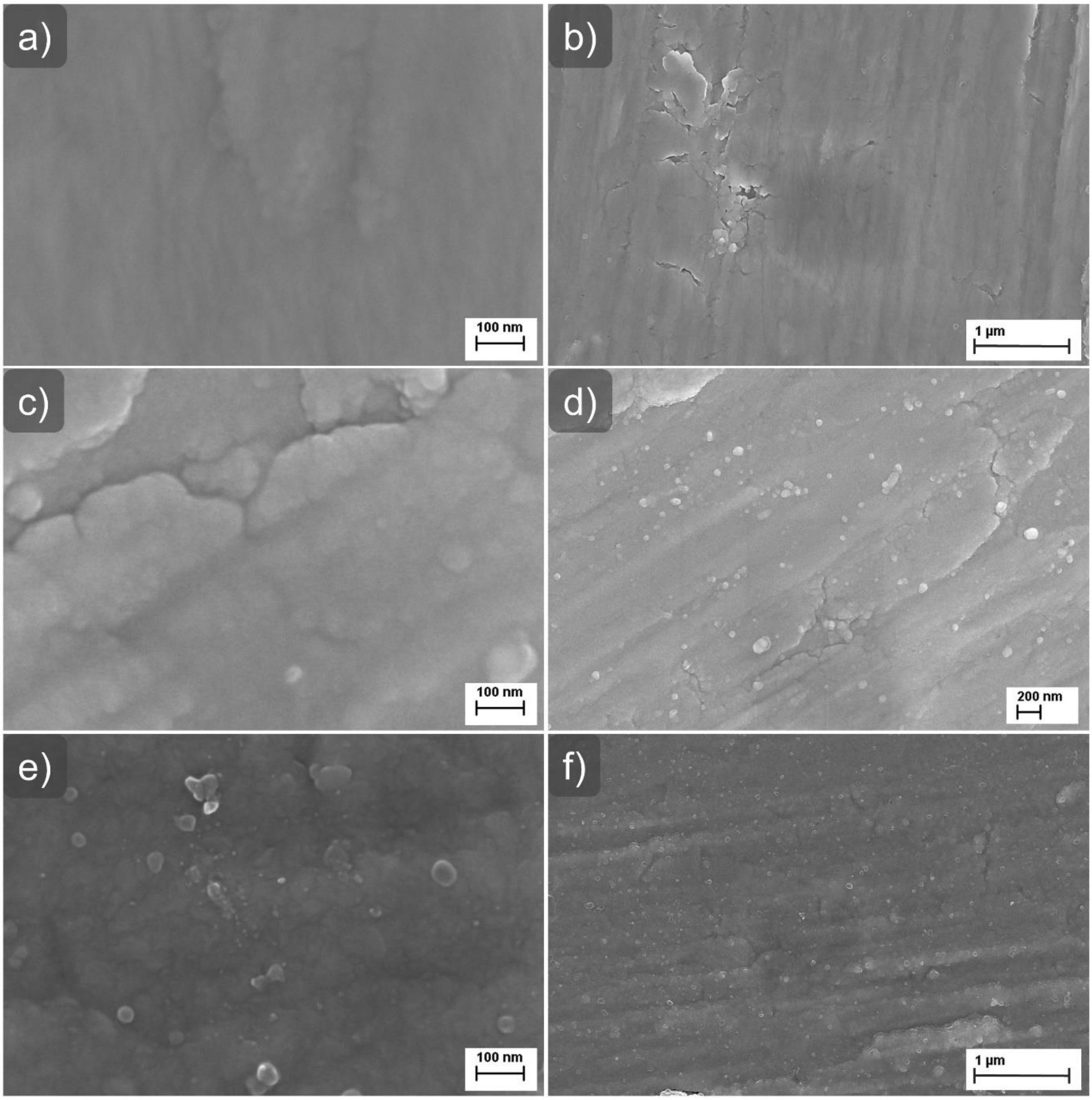


**Figure S15:** The top surface morphology of app. 20-nm-thick (a and b), app. 200-nm-thick (c and d), and app. 1000-nm-thick (e and f) sputtered Ti–Ir films at higher (a, c and e) and lower (b, d and f) magnification.

The top surface morphology of 20-nm-thick, 200-nm-thick, and 1000-nm-thick Ti-Ir alloys is shown in **Figure S15**. Low-magnification views show homogeneous 200-nm-thick and 1000-nm-thick Ti–Ir films on the entire Ti substrate. Low-magnification view of the 20-nm-thick Ti–Ir film shows a few small regions without Ti–Ir grains (**Figure S15b**). The high-magnification views reveal that with increasing the sputtering time, the grain size of the film increased.

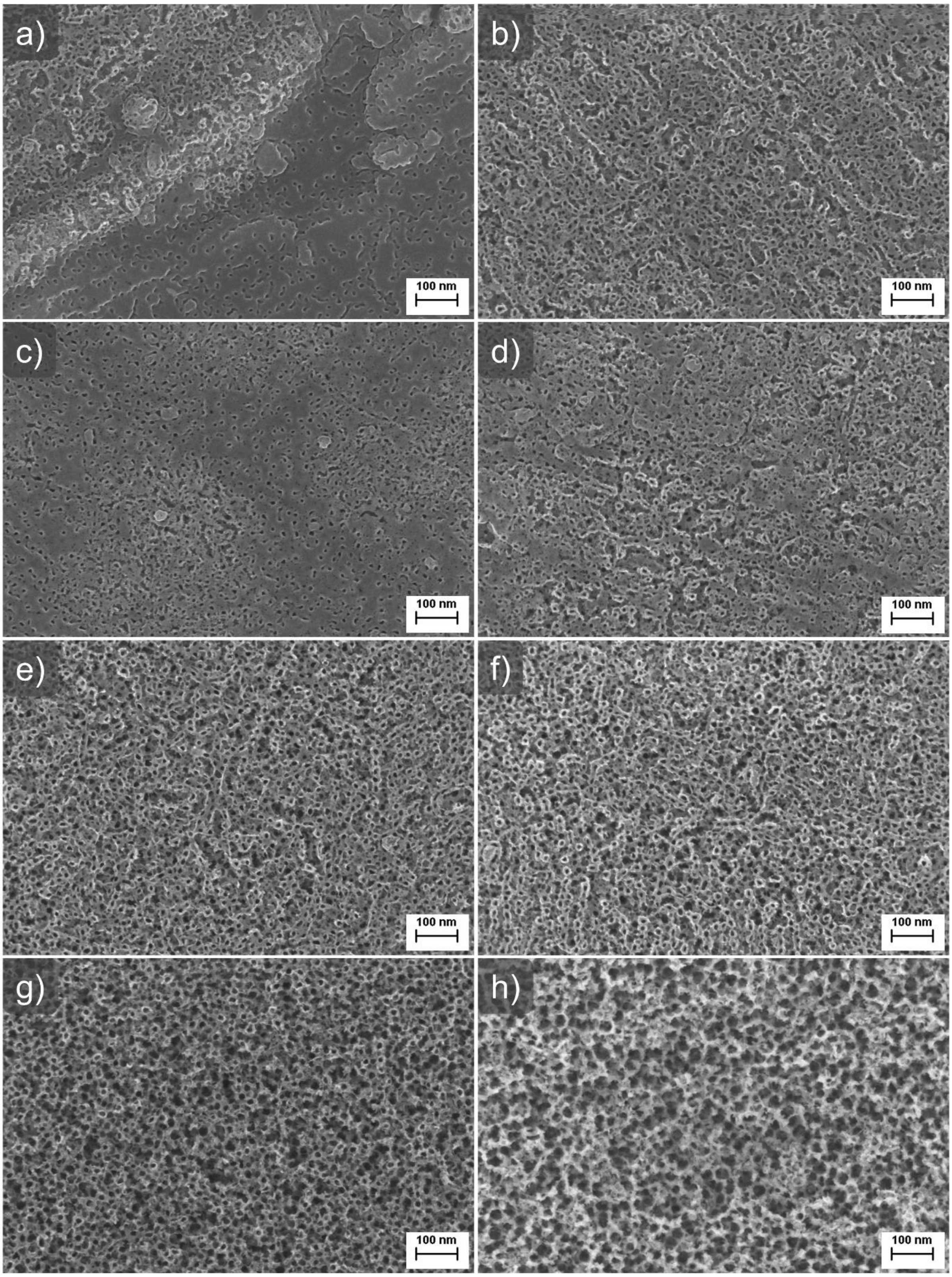


**Figure S16:** The influence of the anodization time on the top surface morphology of anodized 200-nm-thick Ti–Ir films. Anodizations were performed with used electrolyte at 60 V for the following durations: a) 5 s, b) 15 s, c) 30 s, d) 1 min, e) 2 min, f) 3 min, g) 5 min, and h) 8 min.

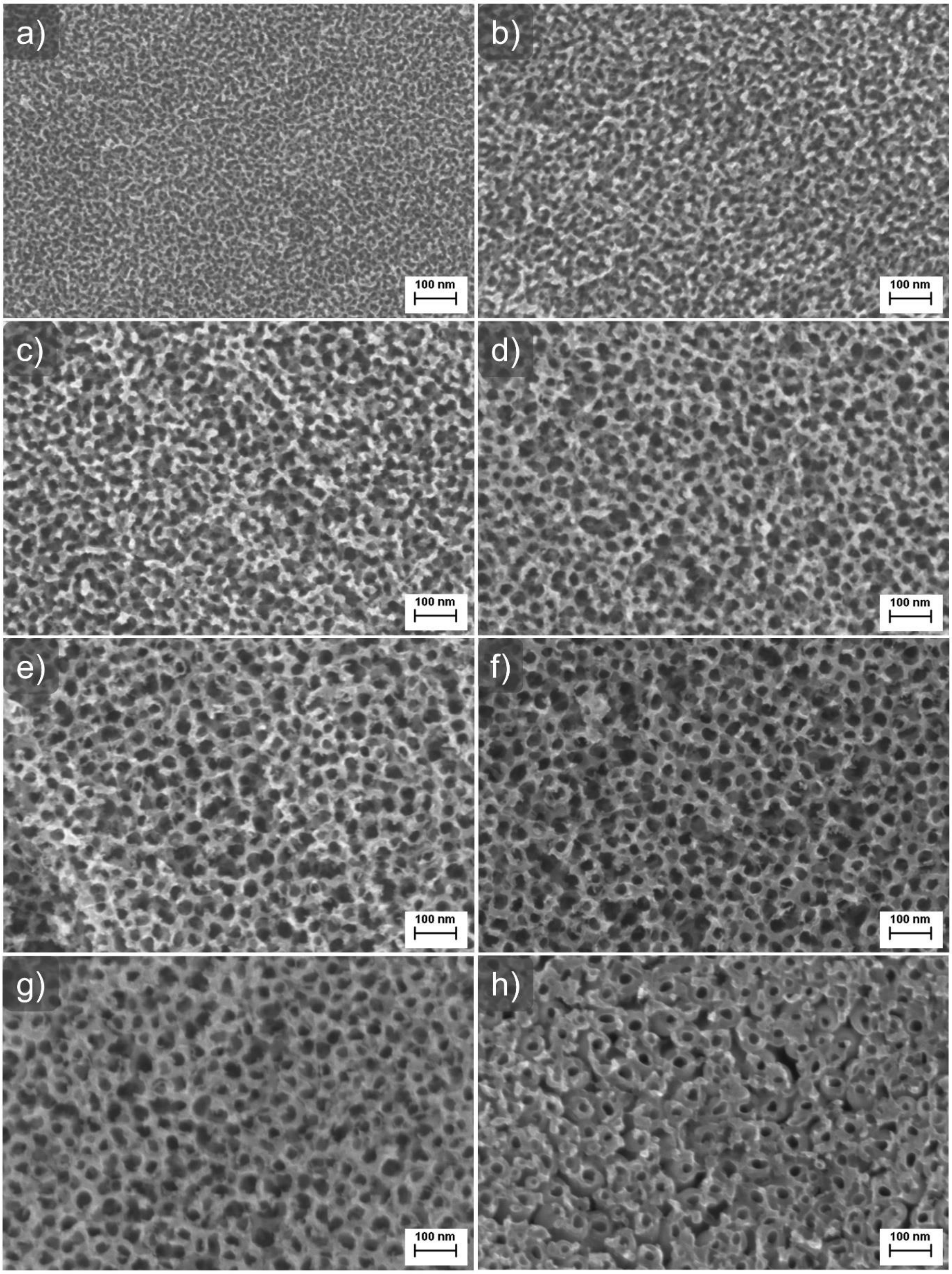
a)
100 nm
b)
100 nm
c)
100 nm
d)
100 nm
e)
100 nm
f)
100 nm
g)
100 nm
h)
100 nm

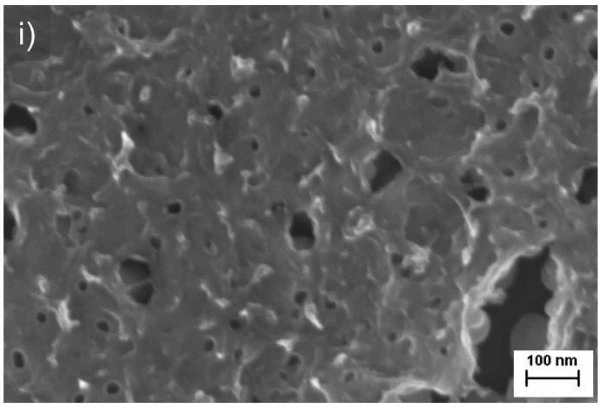


**Figure S17:** The influence of the anodization voltage on the top surface morphology of anodized app. 200-nm-thick Ti–Ir films. Anodizations were performed with fresh electrolyte for 5 min at the following voltages: a) 10 V, b) 20 V, c) 30 V, d) 40 V, e) 50 V, f) 60 V, g) 80 V, h) 100 V, and i) 120 V.

Anodization at 80 V and 100 V results in nanotubular film (**Figure S17**). The film at 80 V has many exploded regions which are shown in **Figure S32**. The nanotubular film prepared at 100 V additionally shows many steps due to many regions with different nanotube length, whereas anodized film observed at 120 V shows no nanotubes and additionally peels-off during anodization.

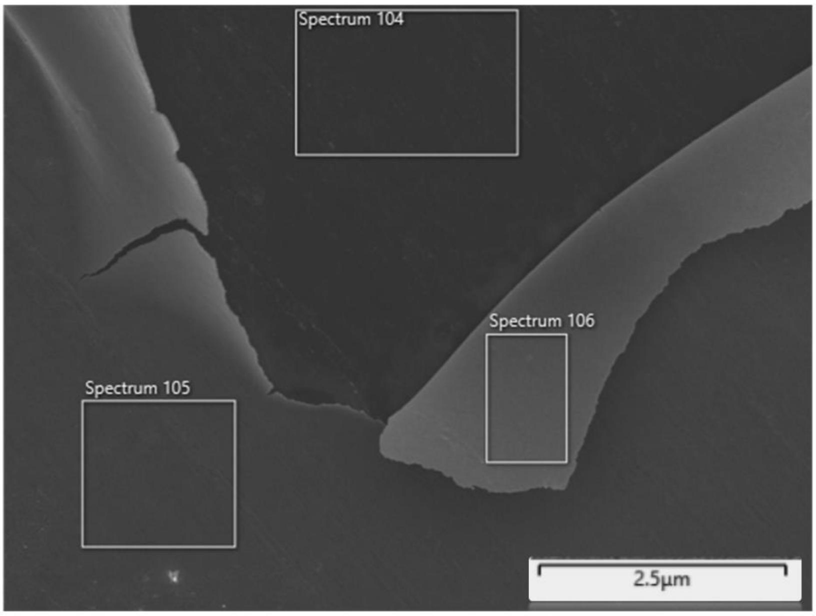


**Figure S18:** Regions where chemical composition of app. 200-nm-thick Ti–Ir film anodized at 60 V for 5 min, annealed in air at 450 °C for 1 h and at 700 °C in $NH_3$ for 15 min shown in **Table S4** was determined. The anodized film peeled-off during ammonia annealing.

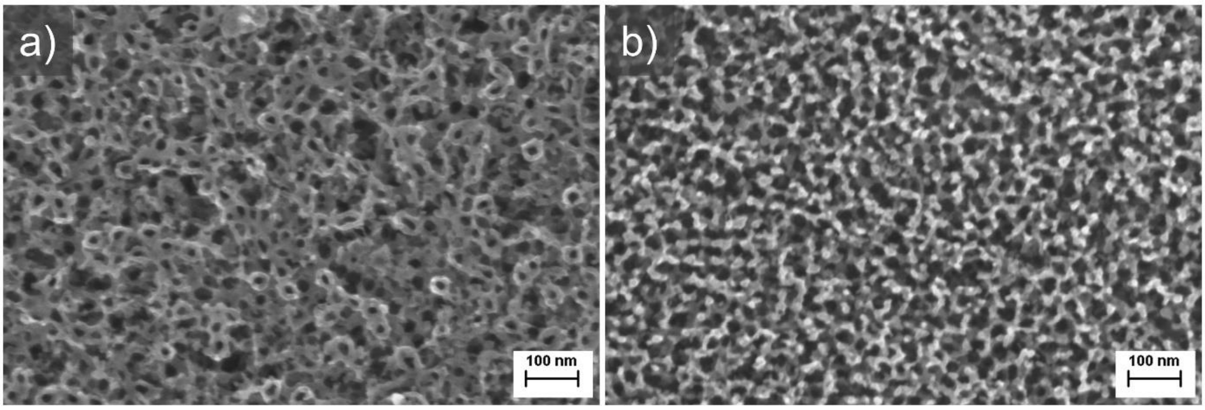


**Figure S19:** Ammonia annealing at 700 °C does not significantly influences the top surface morphology of anodized 200-nm-thick Ti–Ir films when anodization is performed at a) 60 V for 2 min or more or b) for 5 min at 30 V and more.

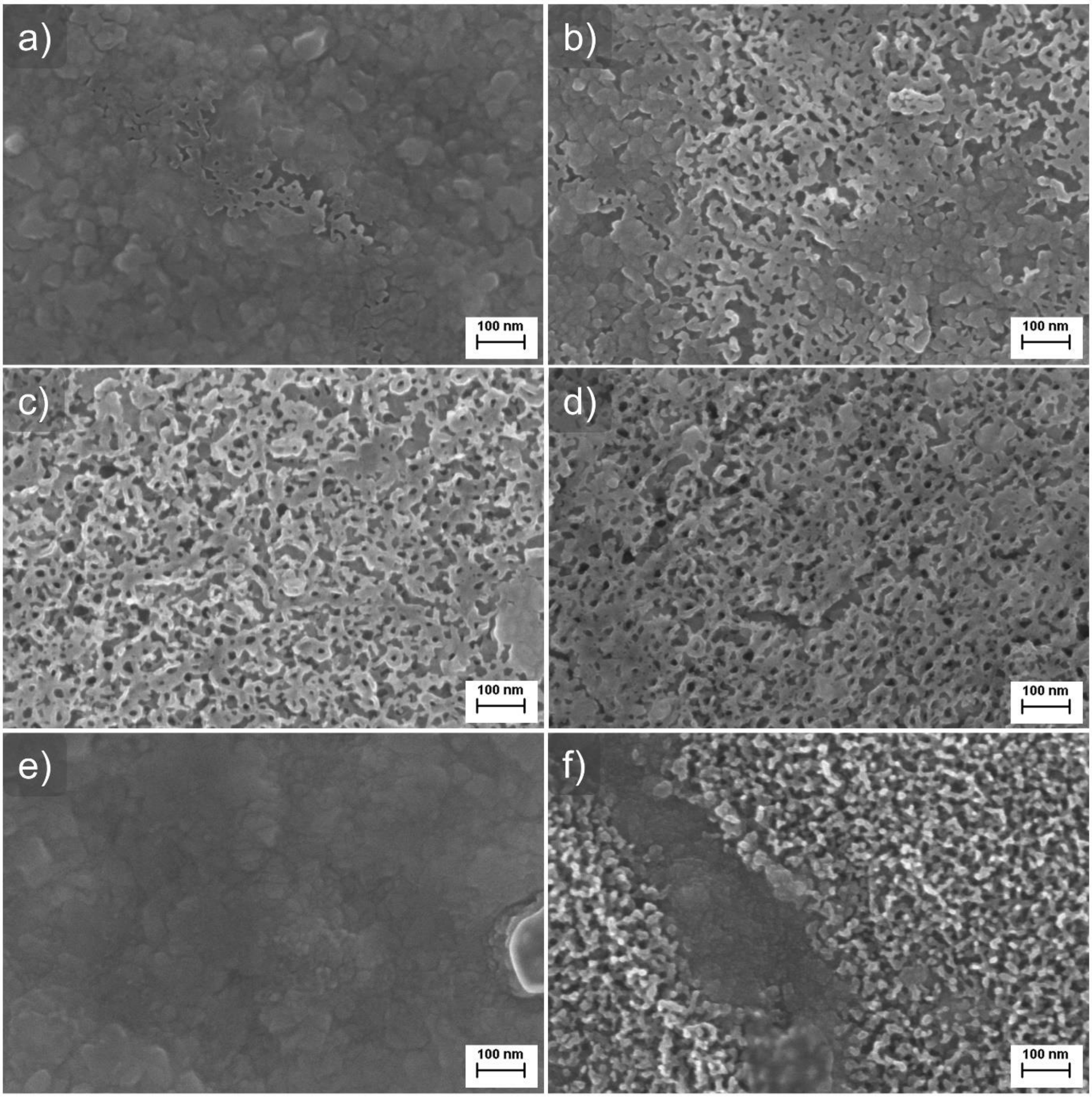


**Figure S20:** The influence of the ammonia annealing time at 700 °C on the top surface morphology of anodized 200-nm-thick Ti–Ir films. Anodizations were performed at the following anodization voltages and time: a) 60 V for 5 s, b) 60 V for 15 s, c) 60 V for 30 s, d) 60 V for 1 min, e) 10 V for 5 min, and f) 20 V for 5 min.

**Figure S19** shows samples which were successfully nitridated at 700 °C for 15 min. These nitridation conditions are optimal for anodized Ti–Ir films with app. film thickness of 400 nm and allow the N/O ratio to be varied with $NH_3$ flow rate. Increasing the flow rate from 50 $cm^3$ $min^{-1}$ to 300 $cm^3$ $min^{-1}$ increases the N/O ratio from app. 0.5 to app. 0.8. Extending the nitridation time to 2 h results in no Ir and nanostructured morphology, whereas shortening the nitridation time to 5 min results in reduction of the amount of nitrogen, a decrease of N/O ratio and consequently in the decreased electronic conductivity of the catalytic film.

If the anodized film thickness is too low, the film colour does not turn into black which is observed for ammonia-annealed films anodized for 2 min or more at 60 V and 30 V or more for 5 min. However, the complete disappearance of the anodized Ti–Ir film during 15-min-long nitridation is only observed for sample anodized at 10 V for 5 min (**Figure S20e**), whereas other samples show significantly changed top surface morphology with regions with the nanostructured film (**Figure S20a-d,f**). All mentioned samples show a significant reduction in Ir amount in the film, which does not occur with samples that show no detrimental change in film morphology (**Table S5**).

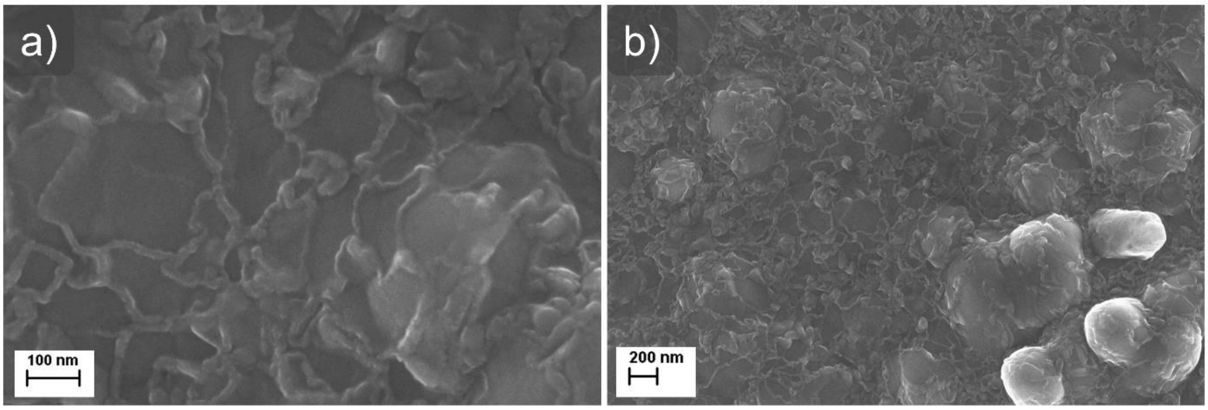


**Figure S21:** SEM micrograph of app. 1000-nm-thick Ti–Ir film anodized at 60 V for 5 min, annealed in air at 450 °C for 1 h and at 700 °C in $NH_3$ for 2 h at a) higher and b) lower magnification. $TiO_2$–Ir morphology has disappeared during annealing in $NH_3$.

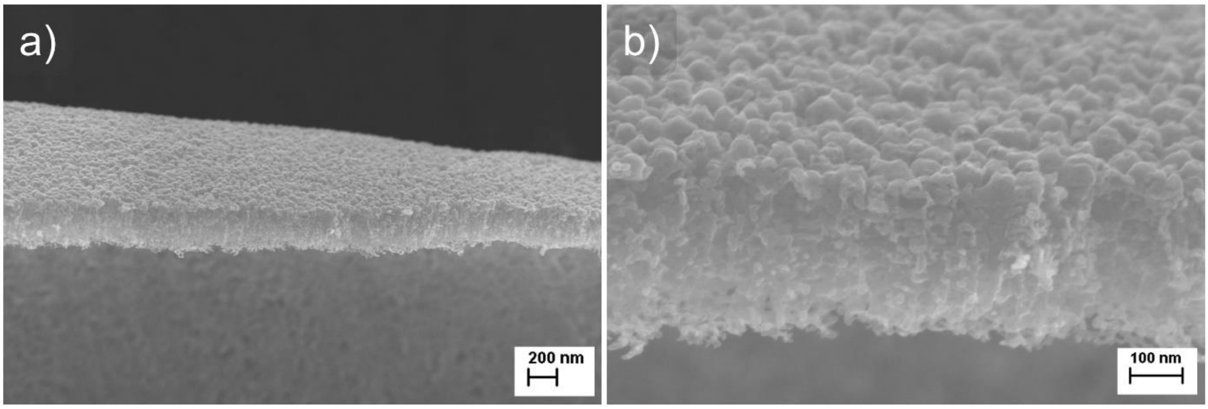


**Figure S22:** The morphology of the delaminated part of $TiO_xN_y$–Ir catalyst prepared at the same conditions as the final optimized $TiO_xN_y$–Ir catalyst: (a) lower magnification of the delaminated part, and (b) higher magnification of the delaminated part. The cross-section shows the thickness of the catalyst to be app. 380 nm. The bottom part of the film (top part of figure b) as well as the cross section show the film is made of less-defined nanotubular structure.

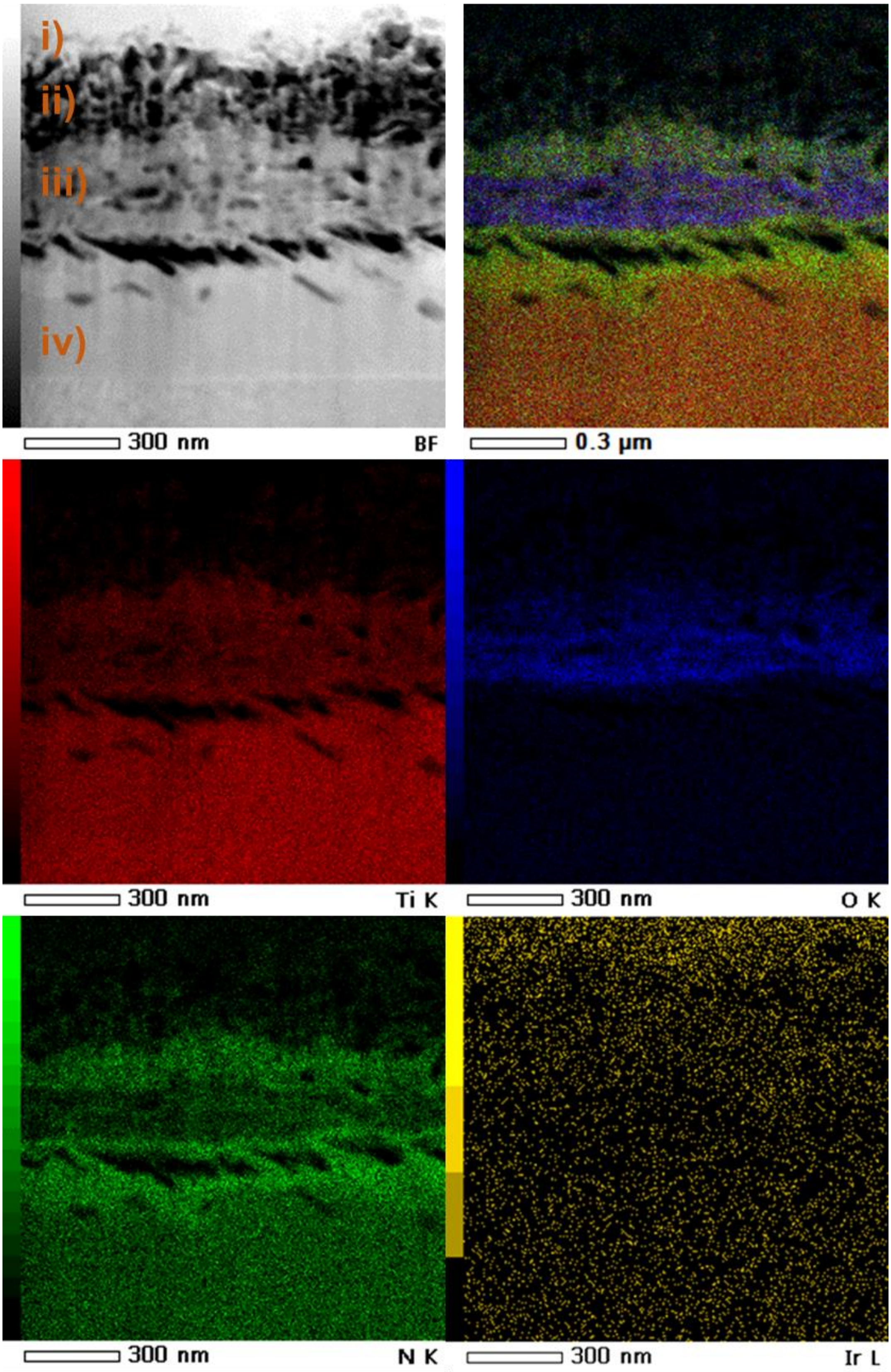


**Figure S23:** STEM energy dispersive X-ray spectroscopy (EDXS) elemental maps of Ti, O, N, and Ir in the cross-section of $TiO_xN_y$–Ir film. In the top left, i) denotes C protective layer, ii) $TiO_xN_y$–Ir film with larger pores, iii) $TiO_xN_y$–Ir film with smaller pores, and iv) Ti substrate.

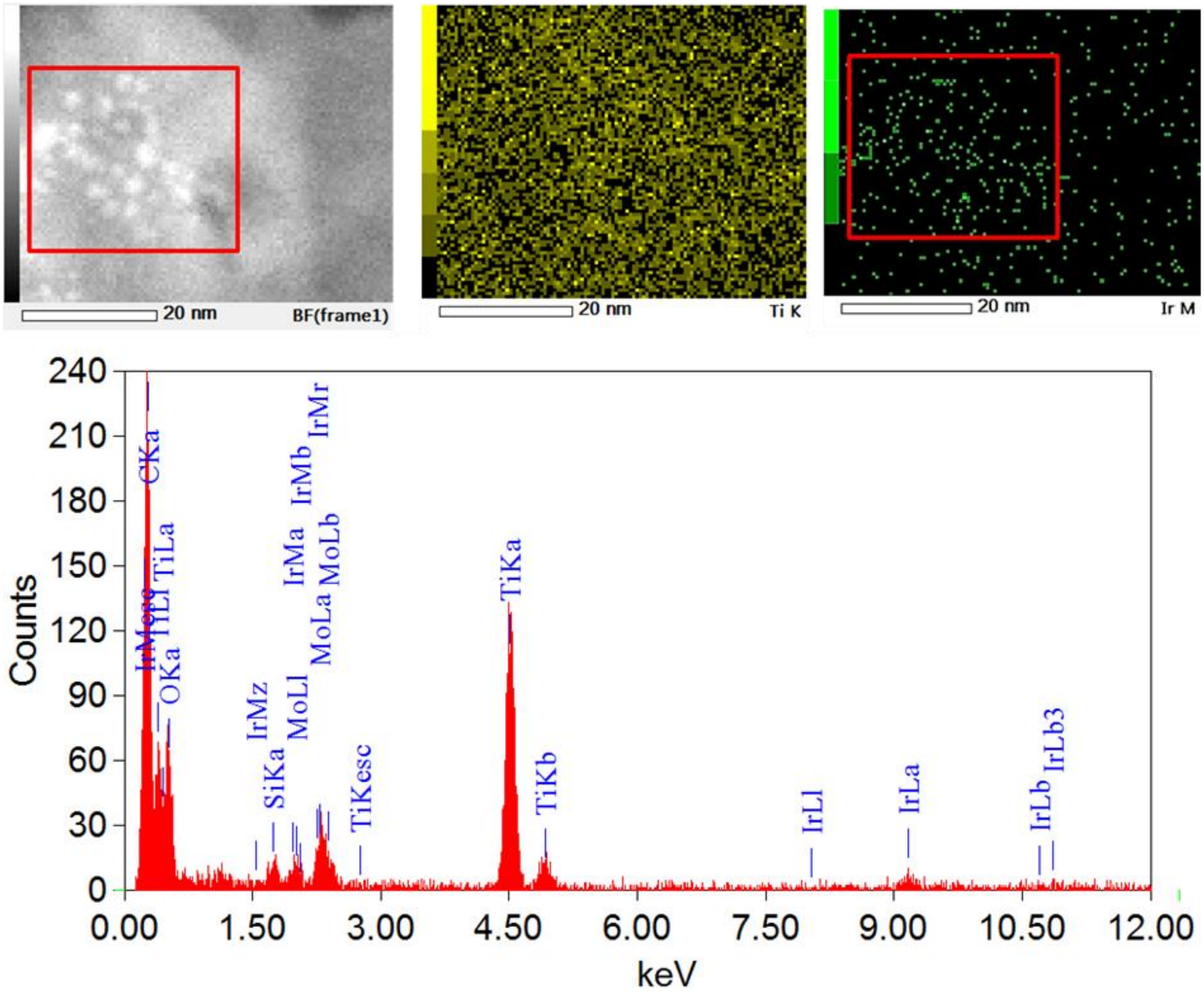


**Figure S24:** High-resolution STEM energy dispersive X-ray spectroscopy (EDXS) analysis of the part of the sample with higher concentration of Ir nano clusters. In the EDXS mapping this area has higher concentration of Ir (1.03 at.% Ir, 41.97 at.% Ti, 46.00 at.% O, 11.00 at.% N). Mo in EDXS spectrum is from FIB lamella holder.

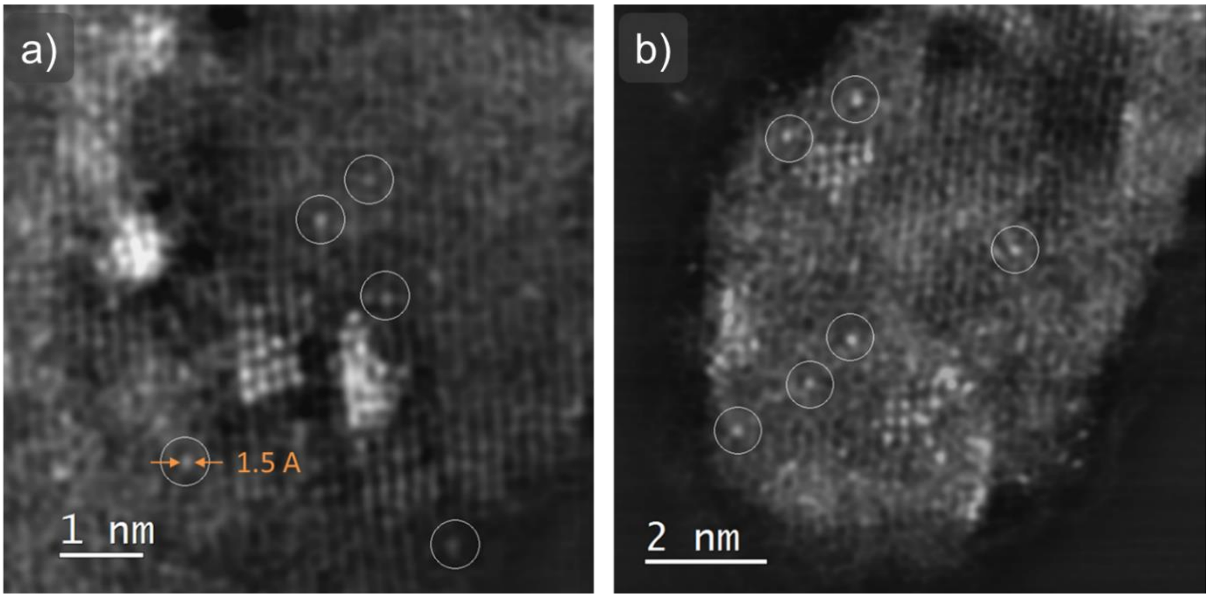


**Figure S25.** HAADF-STEM analysis of $TiO_xN_y$–Ir sample that was scratched on a TEM grid. Ir single atoms can be seen clearly (some of them are encircled with white circles).

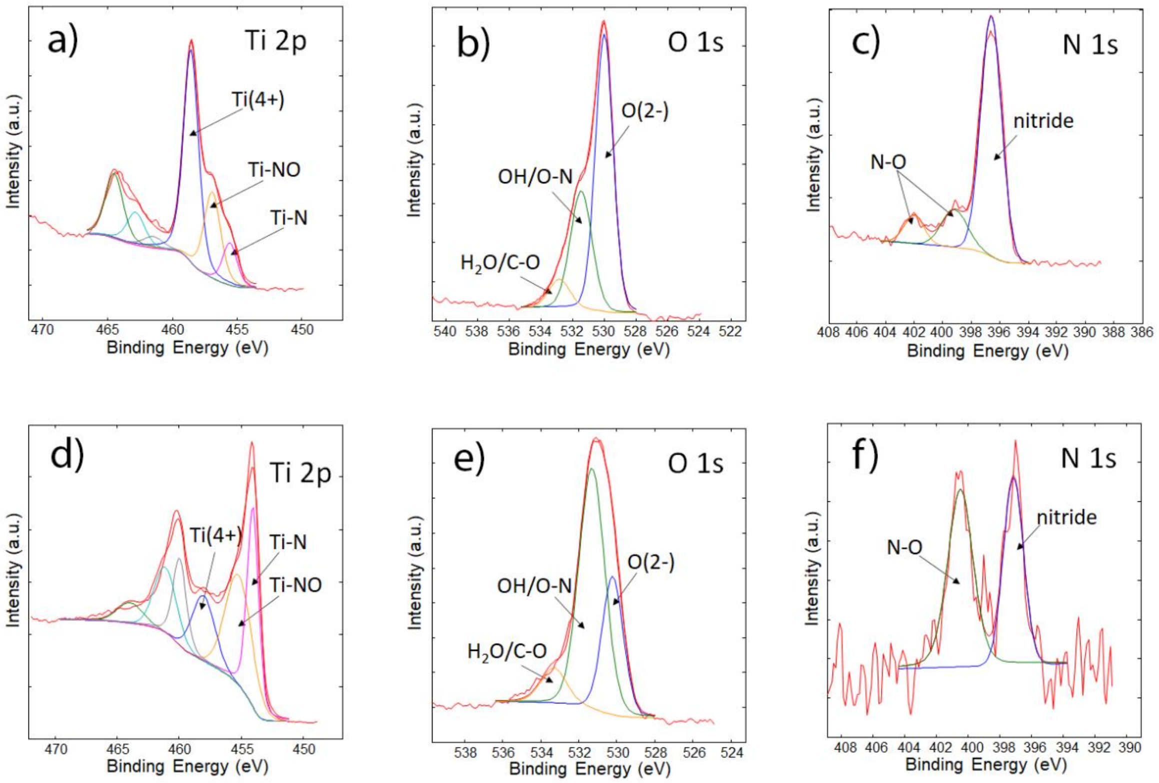


**Figure S26:** Ti 2p, O 1s and N 1s spectra from the surface (a, b and c) and at depth of app. 40 nm (d, e and f) of the $TiO_xN_y$–Ir sample. Spectra measured at depth of app. 40 nm were obtained after XPS depth profile measurement.

**Figure S26** shows XPS spectra of Ti 2p, O 1s and N 1s on the surface and at depth of app. 40 nm. The Ti 2p spectrum consists of peak Ti $2p_{3/2}$ and Ti $2p_{1/2}$ separated by 5.9 eV. On the surface of the $TiO_xN_y$–Ir sample, Ti $2p_{3/2}$ peak is at 458.4 eV which proves the Ti(IV) oxidation state in the $TiO_2$-like structure. In addition to Ti(IV) (60%), peaks at 456.0 eV (12%) and 457.2 eV (28 %) are present which are characteristic for Ti-N and Ti-N-O bonding, respectively. The XPS region of the O1s shows a main peak at 530.0 eV related to $O^{2-}$ anions in the oxide lattice of the $TiO_2$ structure. The additional peaks are attributed to OH– and/or N–O species or oxygen vacancies (a peak at 531.5 eV) and to adsorbed $H_2O$ or C–O based species (a peak at 532.8 eV). The N 1s spectrum consists of a peak at 397 eV related to the nitride and peaks at 399 eV and 402 eV related to oxynitride formation.

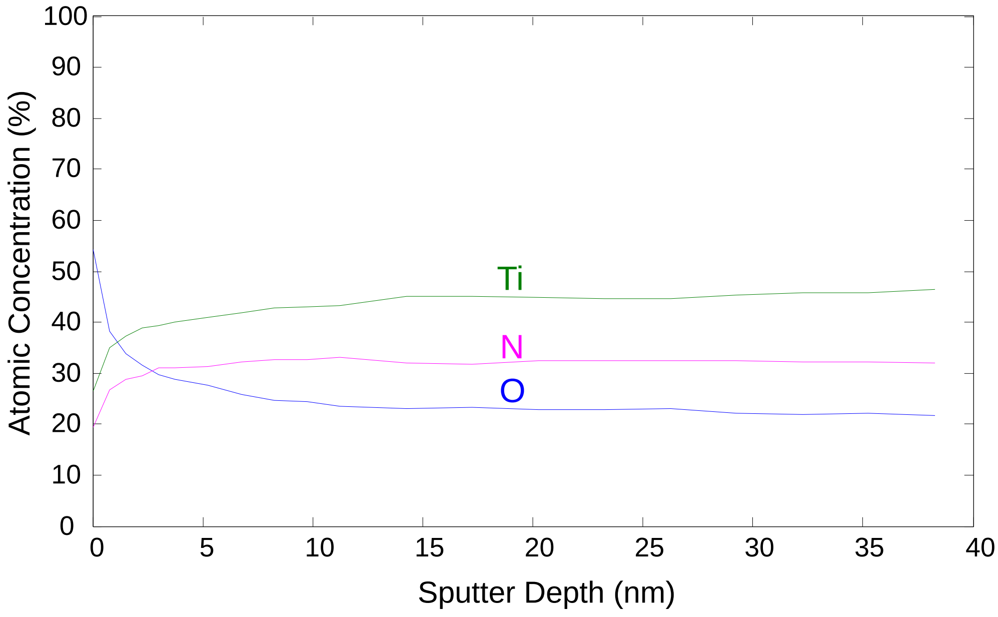


**Figure S27:** XPS depth profile of $TiO_xN_y$–Ir sample. C and Ir (due to low concentration) were not taken into account.

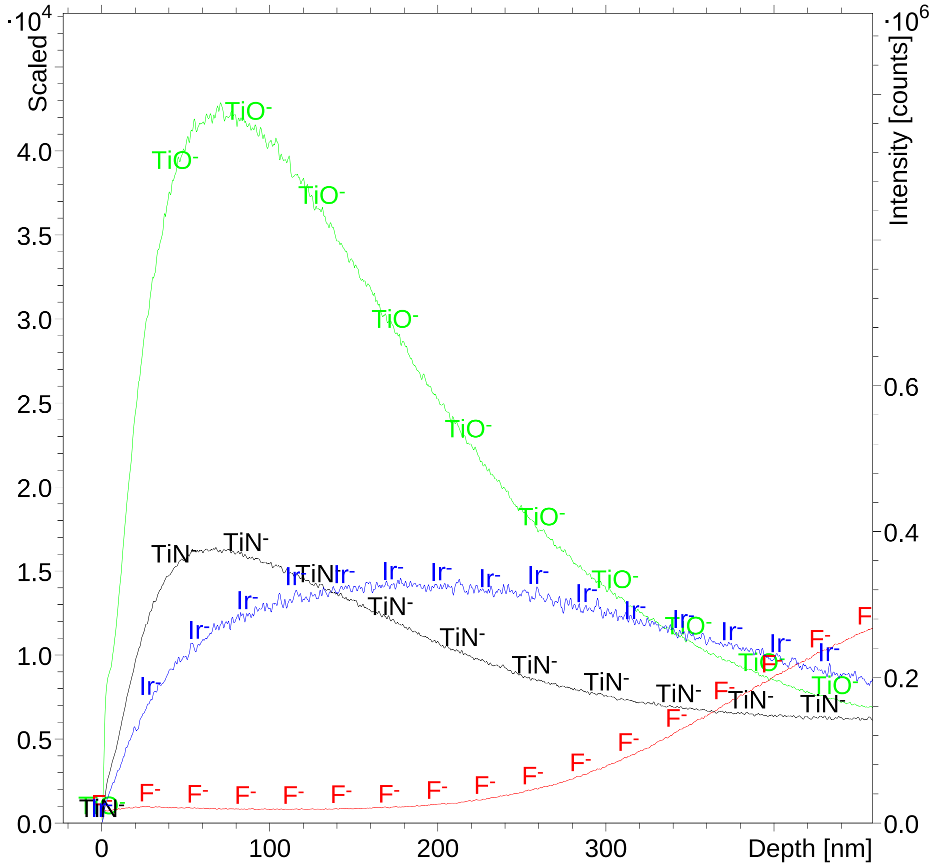


**Figure S28:** ToF-SIMS depth profiles of the app. 200-nm-thick Ti–Ir film anodized at 60 V for 5 min, annealed in air at 450 °C for 1 h and at 700 °C in $NH_3$ for 15 min. Total sputter time of 1800 s corresponds to about 400 nm. SIMS signal of F-ions starts to increase at depth of about 300 nm.

Depth profile of the elements in $TiO_xN_y$–Ir was determined with XPS depth profile measured from the surface to the depth of app. 40 nm (**Figure S27**) and with time-of-flight secondary ion mass spectrometry (ToF–SIMS) measured from the surface to app. 400 nm (**Figure S28**). The advantage of the XPS depth profile is that quantitative concentration curves can be obtained, but the method is of low sensitivity for elements of concentration lower than 1 at.%. The advantage of the ToF–SIMS depth profiling is a fast measurement and higher sensitivity but the quantitative data cannot be obtained.

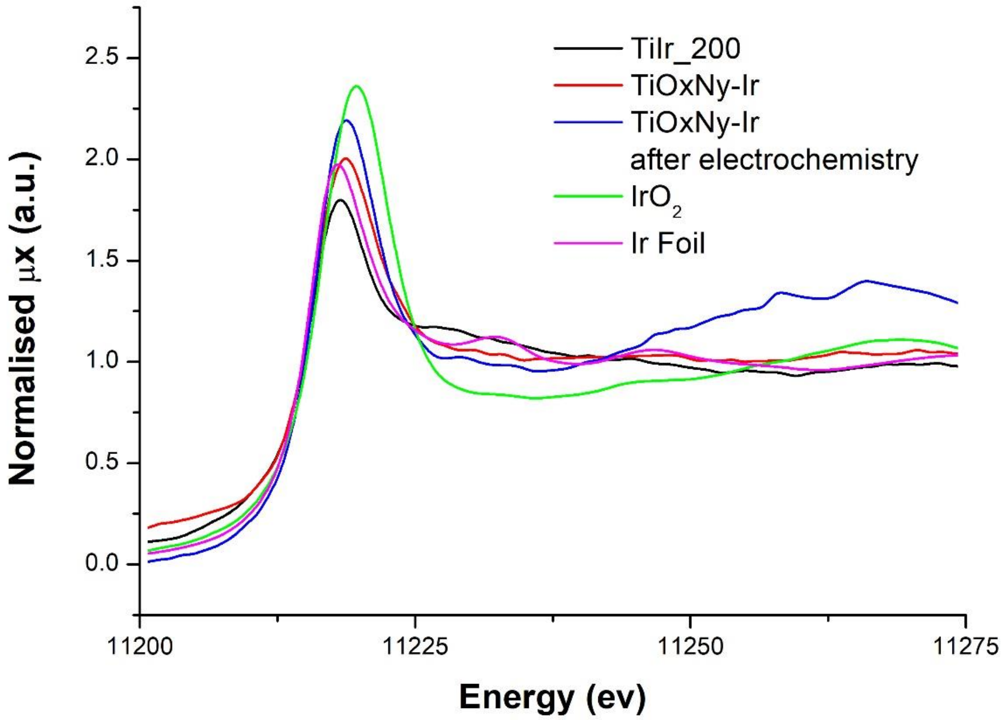


**Figure S29:** XANES spectra of Ti–Ir_200, $TiO_xN_y$–Ir, and $TiO_xN_y$–Ir after electrochemical activity measurement in comparison with referenced materials ($IrO_2$ and Ir foil).

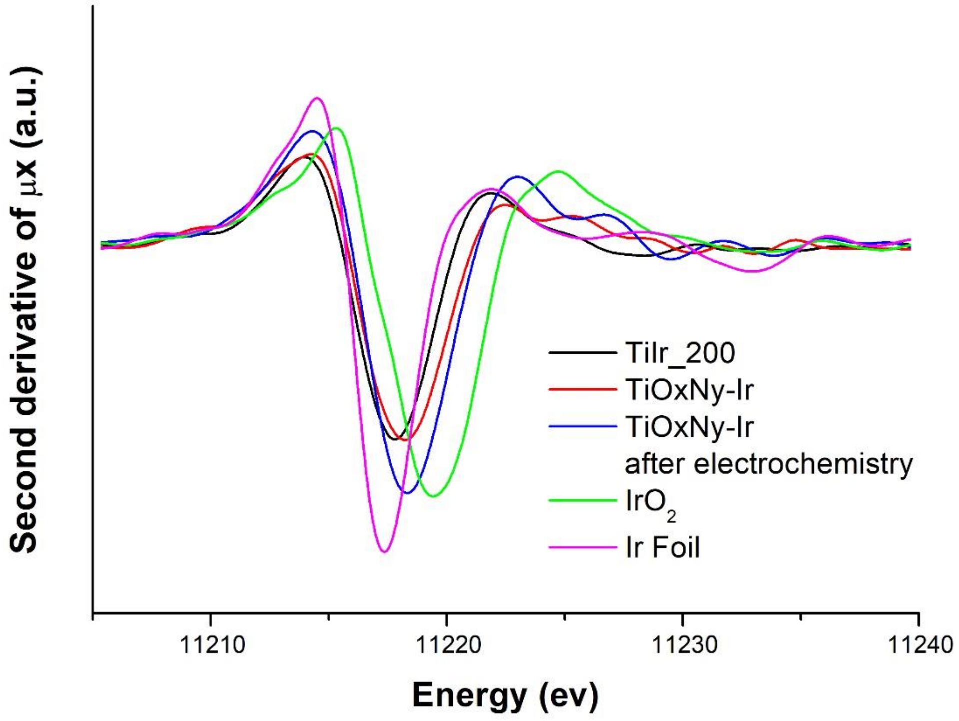


**Figure S30:** Second derivative of XANES spectra of Ti–Ir_200, $TiO_xN_y$–Ir, and $TiO_xN_y$–Ir after electrochemical activity measurement in comparison with referenced materials ($IrO_2$ and Ir foil).

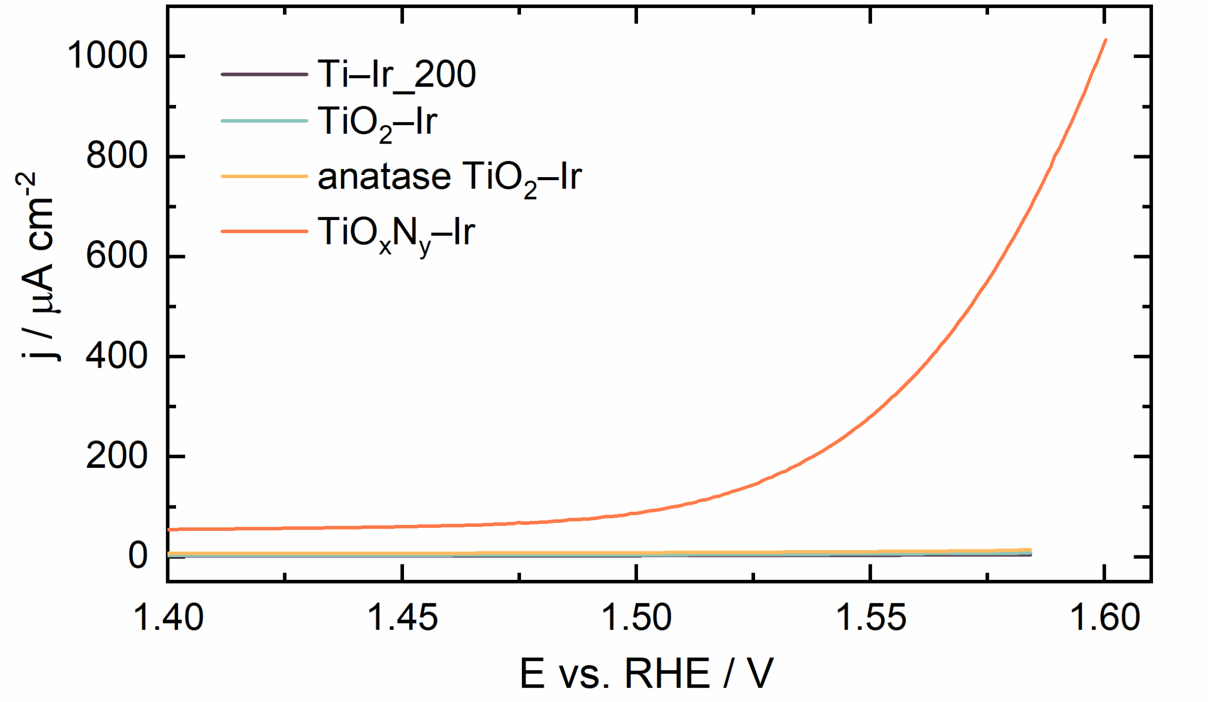


**Figure S31:** Electrocatalytic activity of $TiO_xN_y$–Ir sample in each synthesis step: app. 200-nm-thick Ti–Ir alloy (*purple* line), amorphous $TiO_2$–Ir (*blue* line), anatase $TiO_2$–Ir (*orange* line) and the final $TiO_xN_y$–Ir (*red* line).

Anodization was performed at 60 V for 5 min, annealing in air at 450 °C for 1 h and annealing in ammonia at 700 °C for 15 min in $NH_3$.

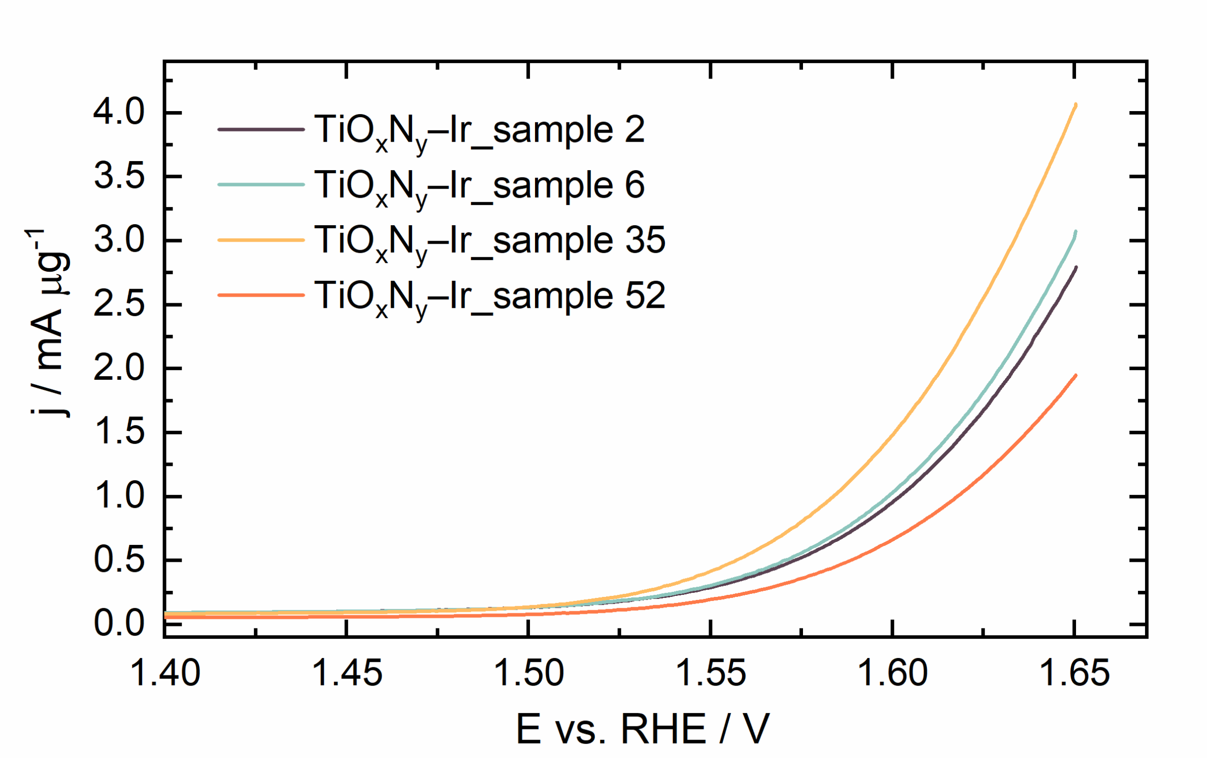


**Figure S32***:* OER polarization curves of the catalysts anodized at 60 V for 5 min with 1-time (*purple* line), 5-times (*blue* line), 34-times (*orange* line), and 51-times (*red* line) used electrolyte. After anodization all the samples were annealed at 450 °C in air for 1 h and at 700 °C in $NH_3$ for 15 min.

The electrocatalytic activity of $TiO_xN_y$–Ir catalysts prepared with anodization electrolyte of different age shows a moderately important effect of the electrolyte age on the catalytic performance. The optimal performance is achieved if anodization electrolyte has not been used for too long which is app. 255 min of anodization. The comparison of electrocatalytic activity of catalyst anodized in 2-times-used (altogether 5 min anodization at 60 V), 6-times-used (altogether 25 min anodization at 60 V) electrolyte, 35-times-used (altogether 170 min anodization at 60 V) and 52-times-used (altogether 255 min anodization at 60 V) electrolyte is shown in **Figure S32**. All the catalysts were anodized at 60 V for 5 min.

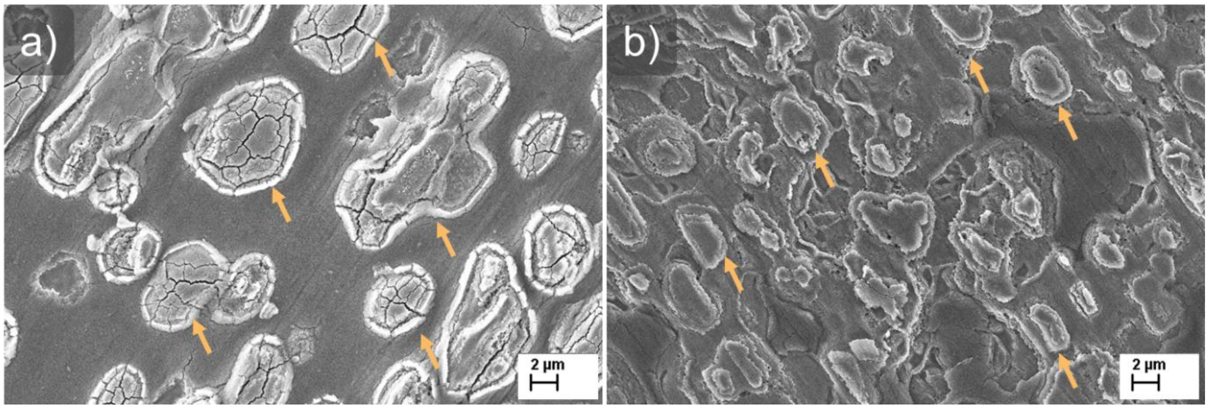


**Figure S33:** "Exploded" regions (orange arrows are pointing to some of them) of anodized films are formed during anodization at high anodization voltages: a) 200-nm-thick Ti–Ir film anodized at 80 V for 5 min, and b) app. 200-nm-thick Ti–Ir film anodized at 100 V for 5 min.

Exploded regions is a phenomenon only observed for films anodized at least at 60 V. It is more pronounced at anodization at 80 V or higher. It is a consequence of sample areas with increased anodization current density which causes the film to "explode" (**Figure S33**) moving the Ir rich film towards the edges while leaving the inner regions without it since the underlying Ti is anodized and bare $TiO_2$ is formed. This is confirmed with the EDXS analysis of the exploded regions showing there is a similar concentration of Ir on the edges of these region as in the starting Ti–Ir alloy. The Ir reach edges are most likely not etched with fluoride ions since their electrical contact with the underlying film and metal is too low.

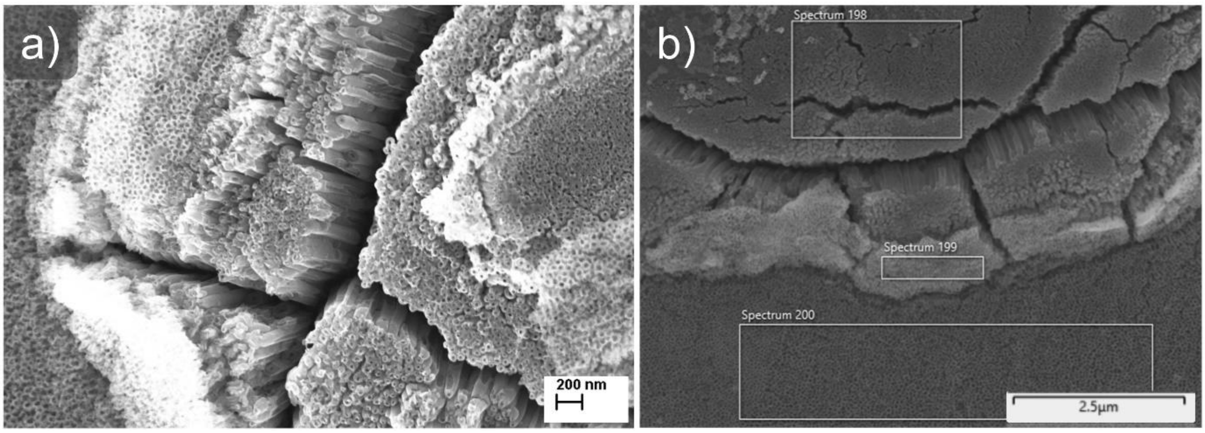


**Figure S34:** a) SEM micrograph of app. 200-nm-thick Ti–Ir film anodized at 80 V for 5 min at higher magnification, and b) three different sample areas analyzed with EDXS (results shown in **Table S6**).

**Figure S34** shows an SEM image of an exploded region at higher magnification which reveals three distinct sample regions with different morphology and composition. The inner part of the exploded region is without Ir and shows a nanotubular film with many cracks. Its thickness is

app. 600 nm. The outer part is rich in Ir and is app. 200 nm thick. The part around the exploded region is made of porous nanotubular film with medium-high Ir content (**Table S6**).

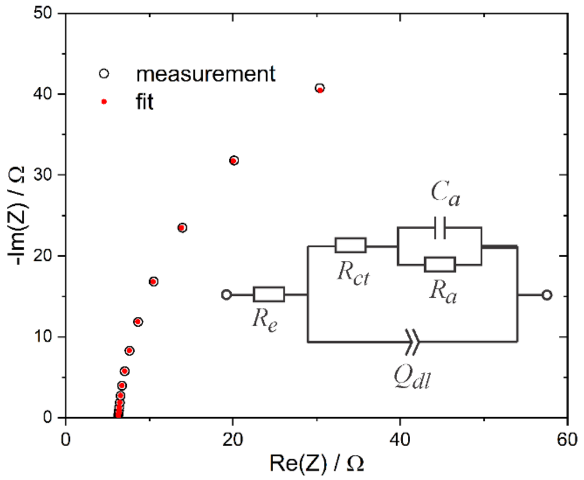


**Figure S35:** Fitted impedance measurement of sample $TiO_xN_y$–Ir_200. The equivalent circuit was taken from reference Watzele et al.[7] The fitted values of elements are the following: $R_e$ = 6.3 Ω, $R_{ct}$ = 83.1 Ω, $R_a$ = 20.3 Ω, $C_a$ = 410 μF, and $Q_{dl}$ = 622 $Ss^{0.95}$.

**Figure S35** shows a typical impedance measurement of sample $TiO_xN_y$–Ir_200 at 1.6 V vs RHE in the frequency range from 5000 to 5 Hz. The measurement was fitted with the equivalent circuit recently proposed by Watzele et al.[7] for analysis of OER catalysts. The values of all elements are given in figure caption. It may be instructive to compare some of these values with the values obtained using the same circuit for a measurement on a standard Ir/C OER catalyst. First we note that the present value of double layer capacitance (in fact double layer constant phase element, $Q_{dl}$), would correspond to a Ir/C catalyst with a total catalyst loading of about 75 µg $cm^{-2}$. Further, at that loading the Ir/Vulcan catalyst would exhibit a $R_{ct}$ of ca. 15 Ω, i.e. about 5.5 less than the present composite. A similar ratio is also found for the so-called adsorption resistance, $R_a$. This comparison shows that the performance of the present composite is about 5-6 less than that for the standard Ir/C catalyst – if the common denominator for comparison is the total conductive surface area giving rise to double layer capacitance. This substantial difference should be ascribed to significantly lower iridium loading for the $TiO_xN_y$–Ir_200 sample.

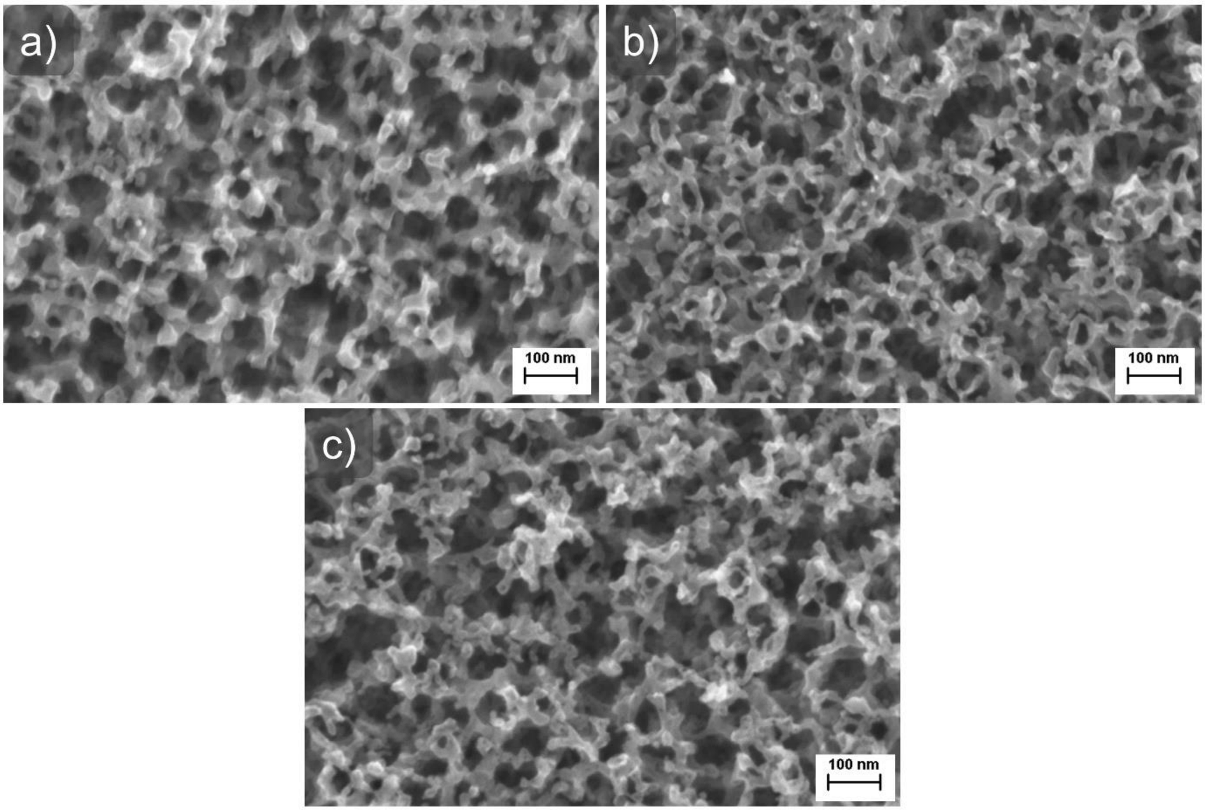


**Figure S36:** SEM micrographs of top surface morphology of the $TiO_xN_y$–Ir catalyst (a) before and (b) after electrocatalytic activity measurement and (c) after electrocatalytic stability measurement.

## Tables:

**Table S1.** Chemical composition of synthetic intermediates of $TiO_xN_y$–Ir catalyst preparation determined with ICP-MS, XPS, SEM–EDXS, and STEM–EDXS. Anodization was performed at 60 V for 5 min in an electrolyte consisting of 0.3 wt.% NH4F and 2 vol.% deionized water in ethylene glycol Annealing in air was performed at 450 °C for 1 h.

| **Ti–Ir_200** | | **200-nm-thick Ti–Ir alloy** | | |
|---|---|---|---|---|
| | **Ti** | **Ir** | | |
| *LA-ICP-MS [μg]* | | 8.87 | | |
| *XPS [wt.%] – depth 5 nm* | 97.3 | 2.7 | | |
| *XPS [at.%] – depth 5 nm* | 99.4 | 0.6 | | |
| *SEM–EDXS [wt.%]* | 96.7 | 3.3 | | |
| *SEM–EDXS [at.%]* | 99.2 | 0.8 | | |
| *STEM–EDXS [wt.%]* | 96.0 | 4.0 | | |
| *STEM–EDXS [at.%]* | 99.0 | 1.0 | | |
| **$TiO_2$–Ir** | | **Anodized Ti–Ir alloy** | | |
| | **Ti** | **O** | **F** | **Ir** |
| *SEM–EDXS [wt.%]* | 64.9 | 26.0 | 8.0 | 1.2 |
| *SEM–EDXS [at.%]* | 41.2 | 46.7 | 11.9 | 0.2 |
| **anatase $TiO_2$–Ir** | | **Air-annealed $TiO_2$–Ir** | | |
| | **Ti** | **O** | **Ir** | |
| *SEM–EDXS [wt.%]* | 67.8 | 30.7 | 1.5 | |
| *SEM–EDXS [at.%]* | 42.3 | 57.4 | 0.2 | |

SEM–EDXS technique was taken into account for each sample, whereas the composition of the sputtered Ti–Ir alloy and $TiO_xN_y$–Ir shown in **Table S1** and **1**, respectively, was additionally determined with ICP–MS, XPS, and STEM–EDXS. XPS analysis of Ti–Ir alloy showed some surface contamination due to the exposure of sputtered samples to ambient air during handling and storage. This is why the XPS analysis of Ti–Ir sputtered film was also performed at depth of 5 nm which is shown in **Table S1**.

The oxygen content is significantly increased during the anodization and further increased during annealing in air at which point the composition of the sample is 57.4 at.% oxygen, 42.3 at.% titanium and 0.2 at.% Ir as determined with SEM–EDXS analysis. The Ti/O ratio does not correspond to $TiO_2$ structure due to the very low thickness of the anodized film if compared to the EDXS analysis depth. The anatase $TiO_2$ structure is confirmed with XRD analysis (**Figure S8 bottom**). As shown in **Table 1** and **Table S1,** the Ir amount in each synthesis step of the $TiO_xN_y$–Ir catalyst preparation differs.

**Table S2:** Chemical composition of $TiO_xN_y$–Ir catalyst prepared with anodization of 20 nm-thick Ti–Ir film at 60 V for 1 min shows no Ir in the film (as determined with EDXS) and app. 0.06 µg Ir as determined with ICP-MS.

| Element | Ti | O | N | Ir |
|---|---|---|---|---|
| **Concentration** | 69.1 | 25.2 | 5.7 | 0.0 |

**Table S3:** Chemical composition of app. 200-nm-thick Ti–Ir film anodized at different voltages for different time. Concentrations are in wt.%. The concentrations were determined with EDXS analysis which detection limit of Ir is app. 0.5 wt.%.

| Anodization conditions | Ti | O | F | Ir |
|---|---|---|---|---|
| **10 V**/5 min | 85.8 | 8.6 | 3.8 | 1.7 |
| **20 V**/5 min | 76.7 | 15.9 | 5.7 | 1.6 |
| **30 V**/5 min | 73.2 | 19.7 | 5.8 | 1.3 |
| **40 V**/5 min | 70.0 | 21.7 | 6.7 | 1.6 |
| **50 V**/5 min | 66.5 | 24.8 | 7.8 | 0.9 |
| **60 V/5 min** | 64.9 | 26.0 | 8.0 | 1.2 |
| **80 V**/5 min | 60.0 | 29.3 | 9.8 | 0.9 |
| **100 V**/5 min | 61.0 | 32.9 | 6.1 | 0.0 |
| 60 V/**8 min** | 58.4 | 30.6 | 10.3 | 0.8 |
| 60 V/**3 min** | 63.5 | 25.7 | 9.6 | 1.3 |
| 60 V/**2 min** | 65.8 | 24.4 | 8.3 | 1.5 |
| 60 V/**1 min** | 66.8 | 19.6 | 12.4 | 1.2 |
| 60 V/**30 s** | 74.3 | 14.9 | 9.3 | 1.5 |
| 60 V/**15 s** | 80.1 | 11.1 | 7.1 | 1.7 |
| 60 V/**5 s** | 82.4 | 10.6 | 5.2 | 1.7 |

EDXS analysis of Ti–Ir film anodized at 60 V for 8 min in aged electrolyte shows only app. 0.8 wt.% Ir which is app. 50 % less than in the samples anodized at 20 V for 5 min or 60 V for 30 s or less. This result is related to **i)** the anodized film thickness and **ii)** the total electric charge passed throughout the circuit in the anodization process which directly influences the $TiO_2$–Ir etching process. In the case of short anodization times or low anodization voltages, Ir from the Ti–Ir substrate is also detected with the EDXS detector due to thin anodized layer if compared to EDXS analysis depth. This happens to a much lesser extent in the case of thicker anodized films prepared with longer anodization times and at higher voltages. It is important to highlight that the Ir that stays in the Ti–Ir film below the anodized film does not contribute to the electrocatalytic activity of such catalysts.

**Table S4:** Chemical composition of app. 200-nm-thick Ti–Ir film anodized at 60 V for 5 min, annealed in air at 450 °C for 1 h and at 700 °C in $NH_3$ for 15 min determined with EDXS analysis and shown in wt.%.

| Spectrum | Ti | O | N | Ir | F |
|---|---|---|---|---|---|
| **104** | 81.2 | 9.4 | 8.9 | 0.0 | 0.5 |
| **105** | 72.0 | 15.5 | 11.3 | 1.1 | 0.0 |
| **106** | 69.3 | 16.7 | 12.1 | 2.0 | 0.0 |

**Table S5:** Chemical composition of app. 200-nm-thick Ti–Ir film anodized at different voltages for different time, annealed in air at 450 °C for 1 h and at 700 °C in $NH_3$ for 15 min. Concentrations are in wt.% and were determined with EDXS (SEM) analysis which detection limit of Ir is app. 0.5 wt.%.

| Sample | Ti | O | N | Ir | F |
|---|---|---|---|---|---|
| **10 V**/5 min, 450 °C/1 h, 700 °C/15 min ($NH_3$) | 72.6 | 17.0 | 9.3 | 0.8 | 0.4 |
| **20 V**/5 min, 450 °C/1 h, 700 °C/15 min ($NH_3$) | 69.1 | 23.1 | 6.5 | 1.3 | 0.0 |
| **30 V**/5 min, 450 °C/1 h, 700 °C/15 min ($NH_3$) | 68.7 | 24.9 | 5.0 | 1.4 | 0.0 |
| **40 V**/5 min, 450 °C/1 h, 700 °C/15 min ($NH_3$) | 68.6 | 24.0 | 6.1 | 1.3 | 0.0 |
| **50 V**/5 min, 450 °C/1 h, 700 °C/15 min ($NH_3$) | 69.7 | 25.0 | 3.9 | 1.4 | 0.0 |
| **60 V/5 min**, 450 °C/1 h, 700 °C/15 min ($NH_3$) | 69.3 | 16.8 | 12.5 | 1.0 | 0.0 |
| 60 V/**8 min**, 450 °C/1 h, 700 °C/15 min ($NH_3$) | 68.7 | 16.6 | 13.7 | 1.0 | 0.0 |
| 60 V/**3 min**, 450 °C/1 h, 700 °C/15 min ($NH_3$) | 70.9 | 18.3 | 9.3 | 1.5 | 0.0 |
| 60 V/**2 min**, 450 °C/1 h, 700 °C/15 min ($NH_3$) | 69.4 | 21.6 | 7.7 | 1.3 | 0.0 |
| 60 V/**1 min**, 450 °C/1 h, 700 °C/15 min ($NH_3$) | 71.0 | 17.6 | 10.4 | 1.0 | 0.0 |
| 60 V/**30 s**, 450 °C/1 h, 700 °C/15 min ($NH_3$) | 73.2 | 18.8 | 6.6 | 0.8 | 0.5 |
| 60 V/**15 s**, 450 °C/1 h, 700 °C/15 min ($NH_3$) | 68.8 | 22.5 | 7.5 | 1.2 | 0.0 |
| 60 V/**5 s**, 450 °C/1 h, 700 °C/15 min ($NH_3$) | 71.6 | 18.3 | 8.7 | 1.1 | 0.4 |

**Table S6:** Chemical composition of sample in **Figure S33** in wt.% as determined with EDXS analysis.

| Spectrum | Ti | O | F | Ir |
|---|---|---|---|---|
| **198** | 61.3 | 32.1 | 6.6 | 0 |
| **199** | 62.0 | 25.6 | 10.2 | 2.2 |
| **200** | 60.2 | 26.9 | 11.4 | 1.5 |

**Table S7**. Comparison of electrochemical performances with Ir-catalysts from literature.

| **Sample** | **A $g^{-1}_{Ir}$ at 1.5 V vs RHE** | **A $g^{-1}_{Ir}$ at 1.55 V vs RHE** | **Tafel slope mV $dec^{-1}$** | **Reference** |
|---|---|---|---|---|
| $TiO_xN_y$–Ir_200 | 140 | 450 | 89 | This work |
| $IrO_2$-AA | 34 | 157 | 67.6 | [8] |
| Ir | | 140-300 | | [9] |

| | | | | |
|---|---|---|---|---|
| Ir/V | | 460 | | 9 |
| Ir/$TiO_2$ | | 70 | | 9 |
| $IrO_x$ | | 689 | | 10 |
| IrNi | | 3353 | | 11 |
| IrCo | | 2327 | | 11 |
| Ir bulk | | | 63 | 12 |
| Ir NP | | | 64 | 12 |
| Ir network (IrCo : 1:55) | | 1850* | | 13 |
| $IrO_2$/TiN | | <250* | | 14 |
| $IrO_x$/ATO | ~ 20* | | 56.8−59.4 | 15 |
| Ir/ATO (11.0 wt.%) | 185 | | 45 | 16 |
| IrRu@Te | 590 | | | 17 |
| $Ir_{SAC}$/ITO | ~ 90 | | 46 | 18 |

*Determined from graphs

**Discussion on ECSA and specific surface area determination of $TiO_xN_y$–Ir catalyst**

Typically, two electrochemical methods for the estimation of ECSA are employed in the literature for Ir-based catalysts. The first one is based on the integration of Ir redox peak between 0.6 and 1.1 V where the corresponding charge is directly correlated to the number of active sites.[19] The second approach calculates ECSA by multiplying roughness factor $R_f$ and real surface area of a smooth electrode S (ECSA= $R_f \times S$).[20] The latter is equal to the geometric area of the electrode, whereas $R_f$ is estimated from the ratio of double-layer capacitance $C_{dl}$ for the catalyst in focus and the corresponding smooth electrode. The $C_{dl}$ of the catalyst is determined by measuring the capacitive current associated with double-layer charging from the scan-rate dependence of cyclic voltammetry stripping. The $C_{dl}$ is estimated by plotting either the anodic or the cathodic current density or the average of the anodic and cathodic current densities against the scan rate.[21]

We note that in our particular case, the usage of any of the reported methods to determine ECSA of Ir SACC is not appropriate due to high capacitive current of $TiO_xN_y$ support and extremely low Ir loading (app. 0.64 µg $cm^{-2}$). The targeted Ir redox peak is therefore not present in the last cycle of the electrochemical activation of our $TiO_xN_y$–Ir SACC catalyst (**Figure S37**). Besides, the reference values of coloumbic charge of Ir and $C_{dl}$ of the corresponding smooth electrode have to be taken from the literature which is questionable since there is no data on the materials studied herein.

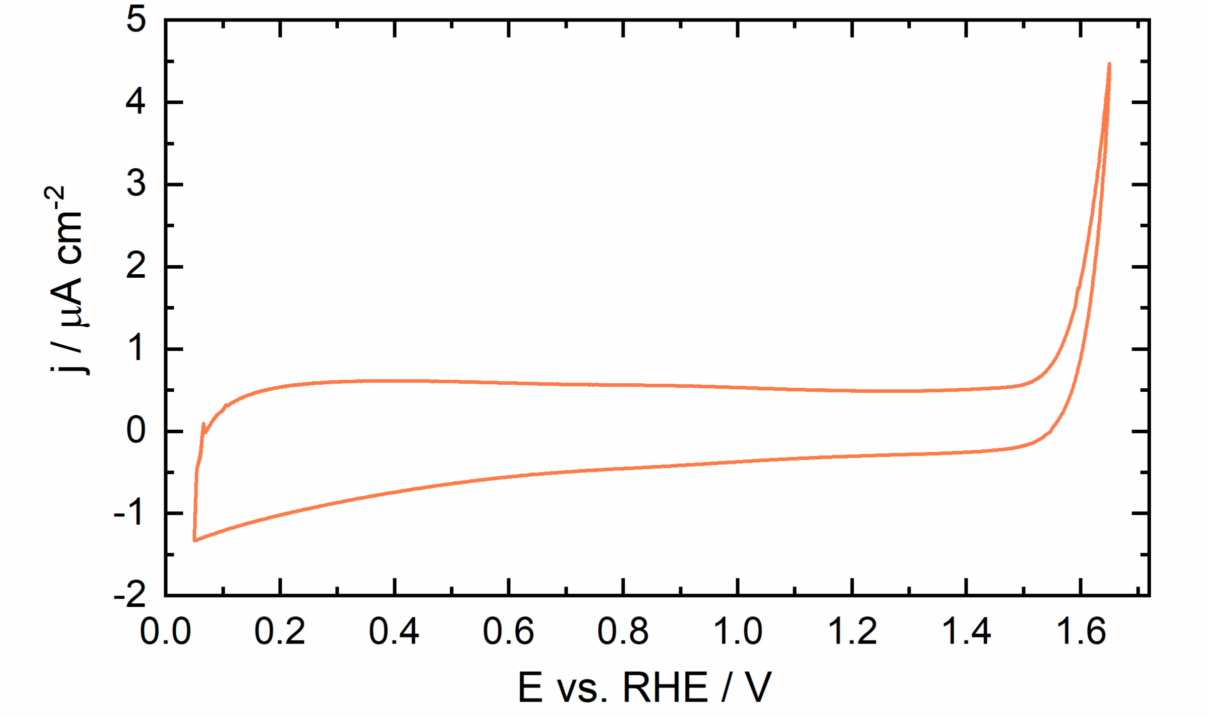


**Figure S37:** Last cycle of the electrochemical activation of $TiO_xN_y$–Ir catalyst showing no Ir peak between 0.6 and 1.1 V.

The second method can be used to give a rough estimation of the specific surface area of the entire $TiO_xN_y$–Ir catalyst, however, it does not result in the ECSA of Ir since its content is too low while the capacitive current of the support is too high.

Therefore, the modified double layer capacitance method is used herein. The modification is in the determination of the $C_{dl}$ for a smooth electrode instead of taking the value from the literature. The problem with taking the $C_{dl}$ value from the literature is that the specific capacitance depends on the electrode potential, surface structure and electrolyte concentration.[22] On the other hand, it is difficult to prepare completely smooth reference material. For this reason, the determined specific surface area is underestimated which was taken into account when reporting the specific surface area of $TiO_xN_y$–Ir catalyst. The specific capacitance was determined in the same way as the $C_{dl}$ for the studied catalyst. Chemically the same material as the catalyst was prepared in as smooth as possible morphology and measured. The reference material synthesis conditions and calculation results are shown in **Table S7**, whereas CV curves at different scan rates for $C_{dl}$ determination are shown in **Figure S38** for the $TiO_xN_y$–Ir catalyst and the reference material. The best estimation of the specific surface area of $TiO_xN_y$–Ir catalyst would be 20 ± 5 $cm^2$.

**Table S7:** Synthesis procedure, double-layer capacitance $C_{dl}$, and roughness factor $R_f$ for the $TiO_xN_y$–Ir catalyst and the corresponding smooth electrode prepared and measured to determine the specific surface area of $TiO_xN_y$–Ir catalyst.

| MATERIAL | SYNTHESIS PROCEDURE | $C_{dl}$ [μF $cm^{-2}$] | $R_f$ [μF $cm^{-2}$] | SPECIFIC SURFACE AREA OF $TiO_xN_y$–Ir catalyst [$cm^2$] |
|---|---|---|---|---|
| $TiO_xN_y$–Ir catalyst | Described in experimental section | 1303 | / | / |
| AO-$TiO_xN_y$–Ir | Anodization of Ti–Ir in $H_3PO_4$-based electrolyte without fluorine, followed by annealing in air at 450 °C for 1 h and $NH_3$ annealing at 700 °C for 15 min | 69 | 19.9 | 14.7 |

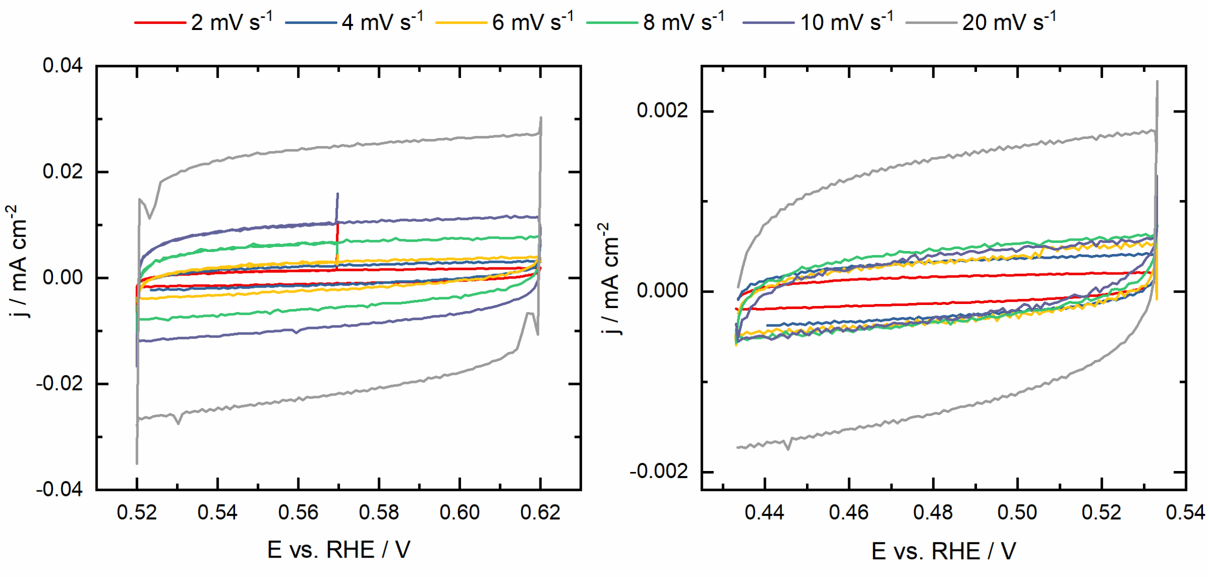


**Figure S38:** CV curves at different scan rates for $C_{dl}$ determination of a) $TiO_xN_y$–Ir catalyst and b) AO-$TiO_xN_y$–Ir reference material. AO stands for anodization of Ti–Ir, however, the anodization in this case was done at 60 V for 5 min in $H_3PO_4$-based electrolyte without fluorine so that a compact oxide was formed instead of nanoporous.

The double layer capacitance method is of significant importance since there is no available method for the determination of the specific surface area of the immobilized films described in the literature. However, the result is still an estimation and the $C_{dl}$ cannot be measured for nonconductive materials. Therefore, the described method can substitute BET method for conductive thin films for which BET surface area cannot be measured. It has to be noted that $C_{dl}$ is a quantitative indicator of the surface area that is accessible to electrolyte ions, whereas BET measures the specific surface area of materials in powder form by isothermal physical adsorption of gas molecules. The two methods are not equivalent; however, it is known from the literature that various particle sizes of $LaCoO_3$ trend well with their $C_{dl}$.[23]

## References


(1) Wang, C.; Guo, Y.; Lu, Y.; Liu, X. Thermodynamic Assessment of the Ti-Ir System. *J Phase Equilibria Diffus* **2014**, *35* (3), 269–275. https://doi.org/10.1007/S11669-014-0302-1/FIGURES/8.

(2) Kabekkodu. PDF-4+. ICCD, International Centre for Diffraction Data: Newton Square, PA, USA 2021.

(3) Marinko, Ž.; Suhadolnik, L.; Samardžija, Z.; Kovač, J.; Čeh, M. The Influence of a Surface Treatment of Metallic Titanium on the Photocatalytic Properties of $TiO_2$ Nanotubes Grown by Anodic Oxidation. *Catalysts* **2020**, 10 (7), 803. https://doi.org/10.3390/catal10070803.

(4) Albu, S. P.; Ghicov, A.; Macak, J. M.; Schmuki, P. 250 Mm Long Anodic TiO2 Nanotubes with Hexagonal Self-Ordering. *Physica Status Solidi - Rapid Research Letters* **2007**, *1* (2), R65–R67. https://doi.org/10.1002/PSSR.200600069.

(5) Albu, S. P.; Schmuki, P. Highly Defined and Ordered Top-Openings in TiO2 Nanotube Arrays. *physica status solidi (RRL) – Rapid Research Letters* **2010**, *4* (7), 151–153. https://doi.org/10.1002/PSSR.201004159.

(6) Ozkan, S.; Nguyen, N. T.; Mazare, A.; Cerri, I.; Schmuki, P. Controlled Spacing of Self-Organized Anodic TiO2 Nanotubes. *Electrochem commun* **2016**, *69*, 76–79. https://doi.org/10.1016/J.ELECOM.2016.06.004.

(7) Watzele, S.; Hauenstein, P.; Liang, Y.; Xue, S.; Fichtner, J.; Garlyyev, B.; Scieszka, D.; Claudel, F.; Maillard, F.; Bandarenka, A. S. Determination of Electroactive Surface Area of Ni-, Co-, Fe-, and Ir-Based Oxide Electrocatalysts. *ACS Catal* **2019**, *9* (10), 9222–9230. https://doi.org/10.1021/ACSCATAL.9B02006/SUPPL_FILE/CS9B02006_SI_001.PDF.

(8) Moriau, L.; Koderman Podboršek, G.; Surca, A. K.; Semsari Parpari, S.; Šala, M.; Petek, U.; Bele, M.; Jovanovič, P.; Genorio, B.; Hodnik, N. Enhancing Iridium Nanoparticles' Oxygen Evolution Reaction Activity and Stability by Adjusting the Coverage of Titanium Oxynitride Flakes on Reduced Graphene Oxide Nanoribbons' Support. *Adv Mater Interfaces* **2021**, *8* (17), 2100900. https://doi.org/10.1002/admi.202100900.

(9) Alia, S. M.; Rasimick, B.; Ngo, C.; Neyerlin, K. C.; Kocha, S. S.; Pylypenko, S.; Xu, H.; Pivovar, B. S. Activity and Durability of Iridium Nanoparticles in the Oxygen Evolution Reaction. *J Electrochem Soc* **2016**, *163* (11), F3105–F3112. https://doi.org/10.1149/2.0151611jes.

(10) Alia, S. M.; Anderson, G. C. Iridium Oxygen Evolution Activity and Durability Baselines in Rotating Disk Electrode Half-Cells. *J Electrochem Soc* **2019**, *166* (4), F282–F294. https://doi.org/10.1149/2.0731904jes.

(11) Alia, S. M.; Shulda, S.; Ngo, C.; Pylypenko, S.; Pivovar, B. S. Iridium-Based Nanowires as Highly Active, Oxygen Evolution Reaction Electrocatalysts. *ACS Catal* **2018**, *8* (3), 2111–2120. https://doi.org/10.1021/acscatal.7b03787.

(12) Reier, T.; Oezaslan, M.; Strasser, P. Electrocatalytic Oxygen Evolution Reaction (OER) on Ru, Ir, and Pt Catalysts: A Comparative Study of Nanoparticles and Bulk Materials. *ACS Catal* **2012**, *2* (8), 1765–1772. https://doi.org/10.1021/cs3003098.

(13) Jensen, A. W.; Sievers, G. W.; Jensen, K. D.; Quinson, J.; Arminio-Ravelo, J. A.; Brüser, V.; Arenz, M.; Escudero-Escribano, M. Self-Supported Nanostructured Iridium-Based Networks as Highly Active Electrocatalysts for Oxygen Evolution in Acidic Media. *J Mater Chem A Mater* **2020**, *8* (3), 1066–1071. https://doi.org/10.1039/c9ta12796h.

(14) Zhang, K.; Mai, W.; Li, J.; Wang, H.; Li, G.; Hu, W. Highly Scattered Ir Oxides on TiN as an Efficient Oxygen Evolution Reaction Electrocatalyst in Acidic Media. *J Mater Sci* **2020**, *55* (8), 3507–3520. https://doi.org/10.1007/s10853-019-04201-4.

(15) Oh, H. S.; Nong, H. N.; Reier, T.; Bergmann, A.; Gliech, M.; Ferreira De Araújo, J.; Willinger, E.; Schlögl, R.; Teschner, D.; Strasser, P. Electrochemical Catalyst-Support Effects and Their Stabilizing Role for IrOx Nanoparticle Catalysts during the Oxygen Evolution Reaction. *J Am Chem Soc* **2016**, *138* (38), 12552–12563. https://doi.org/10.1021/jacs.6b07199.

(16) Hartig-Weiss, A.; Miller, M.; Beyer, H.; Schmitt, A.; Siebel, A.; Freiberg, A. T. S.; Gasteiger, H. A.; El-Sayed, H. A. Iridium Oxide Catalyst Supported on Antimony-Doped Tin Oxide for High Oxygen Evolution Reaction Activity in Acidic Media. *ACS Appl Nano Mater* **2020**, *3* (3), 2185–2196. https://doi.org/10.1021/acsanm.9b02230.

(17) Xu, J.; Lian, Z.; Wei, B.; Li, Y.; Bondarchuk, O.; Zhang, N.; Yu, Z.; Araujo, A.; Amorim, I.; Wang, Z.; Li, B.; Liu, L. Strong Electronic Coupling between Ultrafine Iridium-Ruthenium Nanoclusters and Conductive, Acid-Stable Tellurium Nanoparticle Support for Efficient and Durable Oxygen Evolution in Acidic and Neutral Media. *ACS Catal* **2020**, *10* (6), 3571–3579. https://doi.org/10.1021/acscatal.9b05611.

(18) Lebedev, D.; Ezhov, R.; Heras-Domingo, J.; Comas-Vives, A.; Kaeffer, N.; Willinger, M.; Solans-Monfort, X.; Huang, X.; Pushkar, Y.; Copéret, C. Atomically Dispersed Iridium on Indium Tin Oxide Efficiently Catalyzes Water Oxidation. *ACS Cent Sci* **2020**, *6* (7), 1189–1198. https://doi.org/10.1021/acscentsci.0c00604.

(19) Lončar, A.; Escalera-López, D.; Ruiz-Zepeda, F.; Hrnjić, A.; Šala, M.; Jovanovič, P.; Bele, M.; Cherevko, S.; Hodnik, N. Sacrificial Cu Layer Mediated the Formation of an Active and Stable Supported Iridium Oxygen Evolution Reaction Electrocatalyst. *ACS Catal* **2021**, *11* (20), 12510–12519. https://doi.org/10.1021/ACSCATAL.1C02968/ASSET/IMAGES/LARGE/CS1C02968_0010.JPEG.

(20) Gao, S.; Lin, Y.; Jiao, X.; Sun, Y.; Luo, Q.; Zhang, W.; Li, D.; Yang, J.; Xie, Y. Partially Oxidized Atomic Cobalt Layers for Carbon Dioxide Electroreduction to Liquid Fuel. *Nature* **2016**, *529* (7584), 68–71. https://doi.org/10.1038/nature16455.

(21) Xi, P.; Huang, B.; Yin, J.; Jin, J.; Lu, M.; Zhang, H.; Peng, Y.; Yan, C. H. Iridium Single Atoms Coupling with Oxygen Vacancies Boosts Oxygen Evolution Reaction in Acid Media. *J Am Chem Soc* **2020**, *142* (43), 18378–18386. https://doi.org/10.1021/JACS.0C05050/SUPPL_FILE/JA0C05050_SI_001.PDF.

(22) Wei, C.; Rao, R. R.; Peng, J.; Huang, B.; Stephens, I. E. L.; Risch, M.; Xu, Z. J.; Shao-Horn, Y. Recommended Practices and Benchmark Activity for Hydrogen and Oxygen Electrocatalysis in Water Splitting and Fuel Cells. *Advanced Materials* **2019**, *31* (31), 1806296. https://doi.org/10.1002/ADMA.201806296.

(23) Wei, C.; Feng, Z.; Scherer, G. G.; Barber, J.; Shao-Horn, Y.; Xu, Z. J.; Wei, C.; Xu, Z. J.; Feng, Z.; Scherer, G. G.; Barber, J.; Shao-Horn, Y. Cations in Octahedral Sites: A Descriptor for Oxygen Electrocatalysis on Transition-Metal Spinels. *Advanced Materials* **2017**, *29* (23), 1606800. https://doi.org/10.1002/ADMA.201606800.
